# Integrative clinico-molecular analysis reveals actionable subtypes and biomarkers in lung adenocarcinoma

**DOI:** 10.1038/s41421-025-00863-4

**Published:** 2026-01-28

**Authors:** Jun Shang, He Jiang, Yueren Yan, Yue Zhao, Jingcheng Yang, Han Han, Hui Yuan, Leming Shi, Yuanting Zheng, Haiquan Chen

**Affiliations:** 1 Departments of Thoracic Surgery and State Key Laboratory of Genetic Engineering, Fudan University Shanghai Cancer Center, Shanghai, China; 2 State Key Laboratory of Experimental Hematology, National Clinical Research Center for Blood Diseases, Institute of Hematology & Blood Diseases Hospital, Tianjin Institutes of Health Science, Chinese Academy of Medical Sciences & Peking Union Medical College (CAMS&PUMC), Shanghai, China; 3 Institute of Thoracic Oncology, Fudan University, Shanghai, China; 4 Department of Oncology, Shanghai Medical College, Fudan University, Shanghai, China; 5 Center for Precision Cancer Medicine & Translational Research, Key Laboratory of Cancer Prevention and Therapy, National Clinical Research Center for Cancer, Tianjin’s Clinical Research Center for Cancer, Tianjin Medical University Cancer Institute & Hospital, Tianjin, China; 6 State Key Laboratory of Genetic Engineering, School of Life Sciences, Human Phenome Institute and Shanghai Cancer Center, Fudan University, Shanghai, China; 7 International Human Phenome Institutes (Shanghai), Shanghai, China

**Keywords:** Non-small-cell lung cancer, Targeted therapies

## Abstract

Deeper insights into omics in the clinical and tumor microenvironments of lung adenocarcinoma (LUAD) could reveal therapy-sensitive subtypes and novel treatments. From a cohort of 1008 samples from Chinese patients with LUAD with whole-genome and transcriptome sequencing data along with comprehensive longitudinal clinical and therapeutic information, we identified four prognostically distinct subtypes, namely, low proliferation and invasion (LPI), immune-desert (IMD), immune-enriched (IME), and high proliferation and invasion (HPI), based on the transcriptomic features linked to the radiological, pathological, and microenvironmental dimensions. Compared with chemotherapy, tyrosine kinase inhibitor (TKI) therapy demonstrated significantly superior efficacy for LPI and IMD, whereas no such difference was observed for HPI. VOPP1 and RRM2B amplification were closely associated with TKI resistance and sensitivity, respectively. VOPP1 knockdown restored sensitivity to TKI treatment, while RRM2B knockdown induced TKI resistance, and its overexpression restored sensitivity. Patients with RRM2B amplification had a 5-year survival rate of nearly 100%. Additionally, the IME subtype exhibited higher immune checkpoint activity and a higher frequency of DYNC2H1 mutation, with patients benefiting from immunotherapy. These findings provide critical insights into LUAD treatment optimization.

## Introduction

Lung adenocarcinoma (LUAD) has been detected and treated earlier as a result of the widespread adoption of low-dose CT screening, substantially increasing patients’ overall survival (OS) rate^1,2^. However, LUAD exhibits significant heterogeneity, which is reflected mainly in the different prognoses and treatment strategies for early-stage and mid-late-stage disease. Early-stage cases, such as pre/minimally invasive, lepidic, and pure ground-glass opacity (GGO) LUAD, are almost completely curable through surgery^3,4^, whereas advanced-stage LUAD with metastasis or recurrence is associated with a high mortality risk^5^, necessitating more effective treatment strategies. Molecular subtyping of LUAD promotes stratified treatment and becomes more effective in improving the patient's prognosis. Compared with traditional chemoradiotherapy, targeted therapy based on genomic subtyping has significantly improved OS^6,7^. Receptor tyrosine kinase inhibitors (TKIs), a type of targeted therapy that targets driver genes such as EGFR^8,9^, ALK^10^, ROS1^11^, and RET rearrangements^12^, have shown efficacy. However, high heterogeneity within subgroups challenges the single-gene driver model^13^, and thus, further distinguishing whether patients are sensitive to TKI therapy is difficult. Large-scale omics studies, focusing on quantitative omics (RNA expression^14^, protein expression^15,16^, and multiomics integration^14^), aim to address this heterogeneity. However, quantitative omics models to explain the heterogeneity and guide postoperative treatment of LUAD are still lacking^14–17^.

Molecular subtyping of LUAD, incorporating the radiological, pathological, and tumor microenvironment (TME) dimensions, can help us differentiate patients who respond from those who do not respond to TKI therapy, as the tumor itself (radiological and pathological stage) and the TME collectively determine the progression of LUAD and its response to treatment. GGO radiological features are closely associated with an indolent phenotype, and the pathological stage serves as a crucial determinant for the clinical prognostic classification. However, most omics cohort studies predominantly focus on the pathological stage and often overlook the radiological stage, typically including only solid tumors radiologically. With the increase in the popularity of low-dose CT screening, the prevalence of GGO-LUAD is increasing^18^. The prognosis, surgical approach, and molecular attributes of patients with GGO-LUAD significantly differ from those of patients with solid LUAD^4,19,20^. Hence, integrating GGO-LUAD into omics cohort studies and documenting detailed radiological characteristics facilitates a comprehensive analysis of LUAD heterogeneity. Furthermore, understanding the molecular features of GGO-LUAD aids in prognostic stratification, identifying subgroups with indolent and excellent prognoses. In addition, tumors can interact with surrounding cellular or noncellular components, triggering dramatic molecular changes in the TME to establish a highly structured ecosystem predominantly composed of immune components and stromal components^21^. Moreover, the TME profoundly influences tumor biology, clinical outcomes, and therapeutic responses^22^. The TME also has a significant influence on LUAD progression^23^. Understanding the characteristics of the TME may lead to the optimization of personalized targeted immunotherapies^24^.

Therefore, in this study, inertia and progression features from the tumor itself and its TME were extracted to divide LUAD samples into four subtypes. The LPI (low proliferation and invasion), IMD (immune-desert), IME (immune-enriched), and HPI (high proliferation and invasion) subtypes showed distinct radiological, pathological, and molecular characteristics, prognoses, and therapeutic efficacy, suggesting potential differences in treatment sensitivity among the four subtypes. Variations in therapeutic responses across subtypes have spurred the identification of targets relevant to targeted and immunotherapies. For instance, our study confirmed that a VOPP1 copy number amplification is associated with TKI resistance, whereas an RRM2B copy number amplification increases sensitivity to TKI therapy. Additionally, DYNC2H1 mutations are linked to an improved prognosis for lung adenocarcinoma patients receiving immunotherapy. Our findings demonstrated a holistic developmental process and possible new therapeutic approaches for LUAD.

## Results

### Strategy used to select radiological, pathological, and microenvironmental features for model construction

**Fig. 1:**
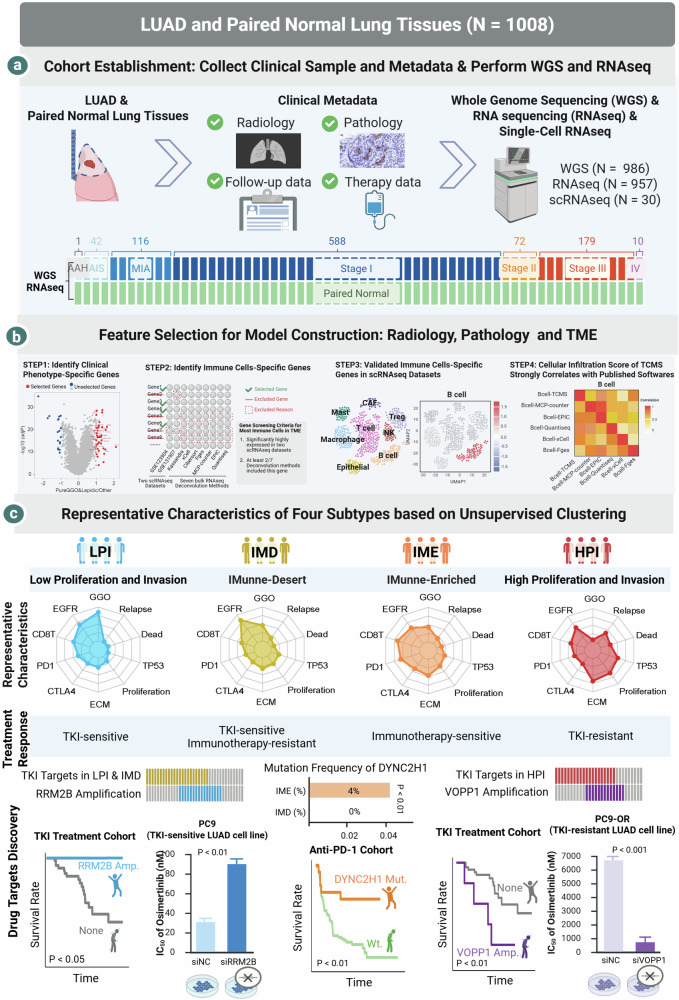
Workflow of molecular subtyping and therapeutic target validation. **a** LUAD patients with resectable tumors were enrolled, and clinical metadata were recorded carefully. Tumor and paired normal lung tissues were collected for whole-genome sequencing (WGS) and RNA sequencing (RNA-seq). **b** In terms of features related to clinical phenotype, genes that were highly expressed in pure/lepidic, solid/micropapillary, and metastatic samples were obtained. With respect to tumor microenvironment-related features, we established a deconvolution method named tumor characteristics and microenvironment scoring (TCMS). TCMS incorporated genes that were significantly highly expressed in two scRNAseq datasets and included after the use of at least two of the seven common deconvolution methods. The cellular infiltration score of TCMS was strongly correlated with that of common deconvolution methods. **c** Representative characteristics, the potential treatment response, and drug target discovery of the four subtypes (LPI, IMD, IME, and HPI) based on unsupervised clustering. LPI and IMD were sensitive to TKI treatment, whereas HPI was resistant. VOPP1 amplification was significantly comutated with TKI therapeutic targets in HPI and was associated with TKI resistance, indicating a poor prognosis for TKI-treated patients. RRM2B amplification significantly co-occurred with TKI therapeutic targets in LPI and IMD patients and was associated with a superior prognosis for TKI-treated patients. IME was potentially sensitive to immunotherapy, whereas IMD was resistant. DYNC2H1 is highly mutated in IME and is associated with a superior prognosis for patients in the immunotherapy cohort.

Here, we present a large-scale molecular characterization of 1008 tumor samples from Chinese patients with LUAD; a longitudinal study with detailed pathological, radiological, and histological information; and survival and recurrence information for more than 10 years. WGS and RNAseq data passed the quality control for 986 and 957 pairs of tumor–normal tissue samples, respectively^25^. Our systematic analysis of these data divided LUAD into four subtypes through comprehensive clinical and TME assessments, identifying key driver pathways and potential new drug targets (Fig. 1).

We quantified radiological, pathological, and TME features using transcriptome-based analyses by investigating the published literature and phenotype-specific genes to identify knowledge-based gene expression signatures representing various features. This method enabled us to develop a holistic approach that comprehensively described radiology, pathology, and the TME in a single model. First, we identified genes that were specifically highly expressed in GGO/lepidic (radiology/pathology), micropapillary/solid (pathology,) and metastatic samples (pathology) and used these genes to characterize radiological and pathological phenotypes that are crucial in clinical practice (Supplementary Fig. S1). Next, we screened genes specifically expressed in the cellular components of the TME by combining the published literature and an analysis of single-cell data (Supplementary Figs. S2 and S3). Finally, the selected TME marker genes were successfully validated in cells using single-cell data (Supplementary Figs. S4–S6). Ultimately, the TCMS (Tumor Characteristics and Microenvironment Score), encompassing 20 radiological, pathological, and TME features, was utilized to characterize the tumors from the macroscopic to microscopic level. We observed a high concordance of the TCMS with most published algorithms scoring TME components (Supplementary Fig. S7).

### Four subtypes identified through the unsupervised clustering of radiological, pathological and TME features

**Fig. 2:**
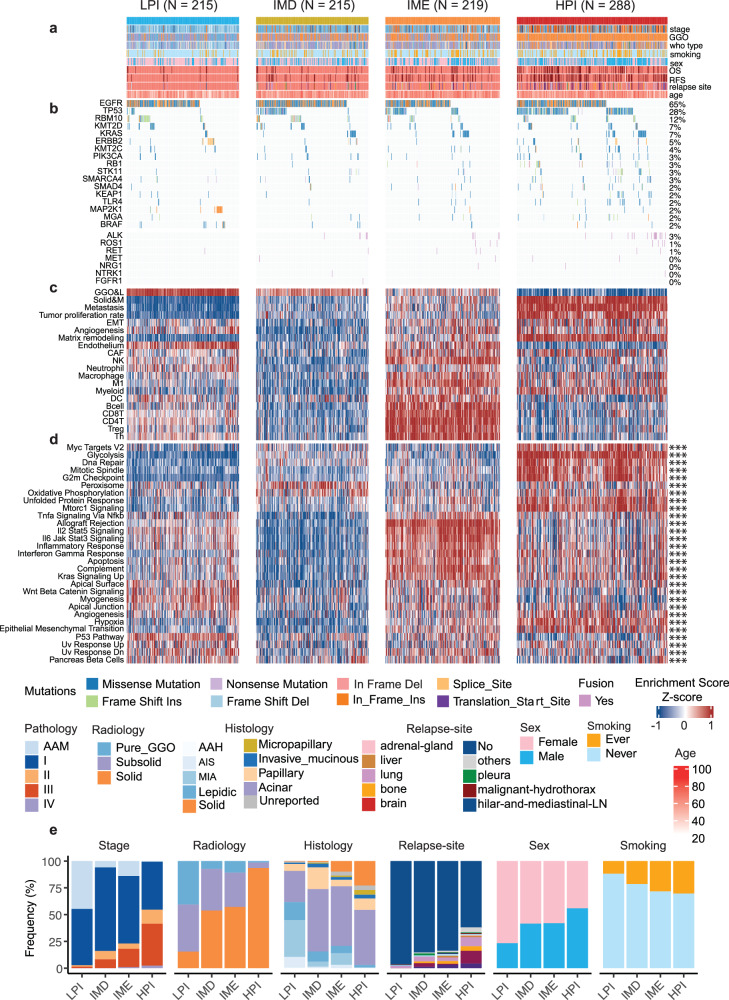
Classification of LUAD into four distinct subtypes based on radio-pathology and TME gene expression signatures. **a** A total of 937 LUAD samples grouped into four distinct subtypes were analyzed, and the heatmap illustrates the distribution of clinical phenotypes, providing a comprehensive view of various clinical characteristics. **b** The oncoplot visually depicts the distributions of genomic alterations and fusions, which were frequently observed in the 937 LUAD samples, across the four distinct subtypes. **c** Heatmap displaying the scores of 20 radio-pathology and tumor environment gene signatures, providing insights into the molecular characteristics of each subtype. **d** The hallmark activity scores in 937 LUAD samples were assessed from the gene expression data obtained through RNA-seq using single-sample gene set enrichment analysis (ssGSEA). **e** The bar plot illustrates the distribution of clinical phenotype frequencies across the four subtypes. ***FDR < 0.001.

By utilizing 20 features from radiology, pathology, and the TME, an unsupervised clustering analysis categorized LUAD into four prognostically distinct subtypes: LPI, IMD, IME, and HPI (Fig. 1c; Supplementary Fig. S8a–d and Table S1). LPI was defined as the highest proportion of patients with GGO nodules, a lepidic histology, the lowest number of tumors with metastasis, the lowest tumor cell proliferation rate, the lowest degree of matrix remodeling, the highest percentage of female never smokers, and the relatively high activity of immune cells in the TME (Fig. 2a; Supplementary Fig. S8d). The substantial overlap observed between the LPI and terminal respiratory unit (TRU) subtypes^14^ underscores the inert nature of the LPI subtype (Supplementary Figs. S9 and S10). The frequency of EGFR mutation in the IMD group was the highest among the four subtypes (Fig. 2b), indicating that it is the subtype most likely to benefit from TKI therapy. The overall activity of immune cells in the IME was the strongest among the four subtypes, and was particularly for CD8^+^ T cells, NK cells, and B cells, whose activity was significantly greater than that of the other three subtypes (Fig. 2c). These findings suggest that patients with the IME subtype may benefit from immunotherapy. HPI was defined as the highest proportion of patients with solid nodules on radiological images, solid and micropapillary nodules on pathological images, the highest risk of metastasis, the highest tumor cell proliferation rate, and the greatest degree of matrix remodeling (Fig. 2c; Supplementary Fig. S8d). Among the three published subtypes of LUAD^14^, HPI almost exclusively overlapped with the proximal proliferative (PP) and proximal inflammatory (PI) subtypes, indicating that the HPI subtype exhibited highly active tumor proliferation and infiltration (Supplementary Figs. S9 and S10). In other words, HPI emerged as the most malignant subtype, characterized by both clinical and molecular features and relatively weak immune cell activity. This finding indicated that it could represent the subtype associated with the fastest tumor progression, highest likelihood of recurrence, and greatest treatment challenge.

We examined the activities of signaling pathways to further elucidate the biology of the four subtypes (Fig. 2d). Mitotic spindles, the G2M checkpoint, and other pathways associated with cell proliferation were significantly upregulated in HPI. Glycolysis, which has been implicated in immune evasion mechanisms in many solid tumors^26^, was also upregulated in HPI. Immune-related pathways such as the interferon gamma response, inflammatory response, and apoptosis were upregulated in the IME subtype. Overall, pathway enrichment analysis revealed that HPI was the most malignant subtype and IME was the most immunoreactive subtype.

In terms of clinical phenotypes, the malignancy level progressively increased from LPI to HPI. LPI predominantly occurred in patients in the very early stage, with a high proportion of patients exhibiting GGO and lepidic phenotypes. It demonstrated a low recurrence rate and was predominantly observed in female nonsmoking patients (Fig. 2e). Conversely, HPI was characterized by a notably higher occurrence among patients with stage II–IV disease. It predominantly presents as a pure solid nodule on a radiological examination, with a minimal lepidic phenotype. Compared with LPI, HPI exhibited a markedly increased incidence of relapse and a higher proportion of male smokers. The distribution of clinical phenotypes in IMD and IME fell between LPI and HPI, showing a relatively even distribution (Fig. 2e).

### Prognosis and molecular characteristics of the four subtypes

**Fig. 3:**
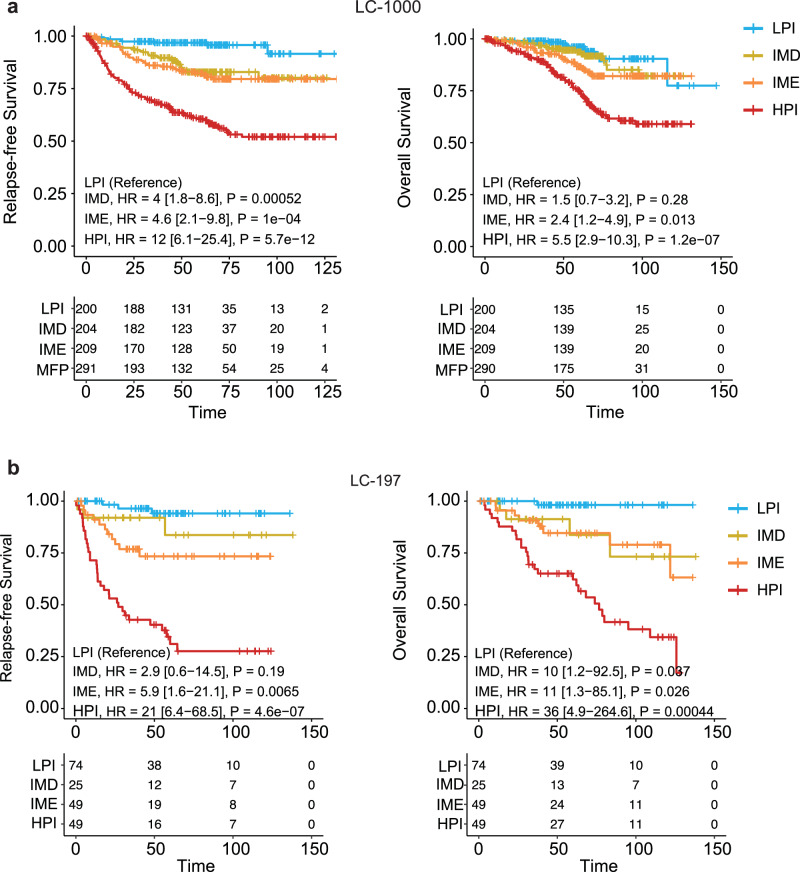
Survival outcomes associated with the four identified subtypes. **a**, **b** The overall survival (OS) and relapse-free survival (RFS) distributions of the four subtypes were analyzed in LUAD patients from the LC-1000 (**a**) and LC-197 (**b**) cohorts.

LPI, IMD, IME, and HPI exhibited significantly divergent prognoses. The prognosis of patients decreased gradually from LPI to HPI (Fig. 3). The prognostic value of the four subtypes was validated in the LC-197 cohort, which included 197 LUAD patients we previously enrolled (Fig. 3; Supplementary Fig. S11). Multivariate Cox analysis also demonstrated the prognostic predictive efficacy of the four subtypes (Supplementary Fig. S11b, c). The four subtypes classified based on unsupervised clustering were also confirmed in TCGA cohort (Supplementary Fig. S11d, e). The molecular profiles of the four subtypes provided further insights into the differences in prognosis and the characteristics of the TME among the subtypes. Gene expression-based hierarchical clustering and PCA revealed significant transcriptomic differences between LPI and HPI, with IMD and IME exhibiting intermediate profiles between LPI and HPI (Supplementary Figs. S12 and S13). The LPI gene expression profile was indicative of early-stage, GGO, and lepidic tumors, whereas the HPI profile corresponded to mid-late-stage and solid tumors (Supplementary Fig. S13a).

The transcriptomic and genomic characteristics of the four subtypes also differed. The differentially expressed genes (DEGs) among the four subtypes were functionally annotated using hallmark genes, providing further internal molecular characterization of each subtype. Cancer hallmarks related to angiogenesis, the epithelial-to-mesenchymal transition (EMT), glycolysis, hypoxia, and cell proliferation were upregulated in HPI, while immune-related hallmarks were upregulated in IME (Supplementary Fig. S13b). At the genomic level, HPI exhibited more active genomic variations, including point mutations and copy number variations, than LPI. Indeed, an increasing trend in the occurrence of genomic events was observed from LPI to HPI. However, we also noted some specific phenomena. For instance, the frequencies of MAP2K1 and ERBB2 mutations were highest in LPI, while the frequency of EGFR mutations was highest in IMD (Supplementary Fig. S13c, d). Variations in these genomic events in the RTK–RAS pathway might lead to differences in the response to targeted therapies among the subtypes (Supplementary Fig. S14). Notably, subtypes strongly influenced the patient prognosis. For example, patients with a GGO component, which is typically associated with a good prognosis, had a worse prognosis if assigned to the HPI subtype than those with pure solid tumors assigned to the LPI subtype did (Supplementary Figs. S15 and S16).

Furthermore, we extensively investigated mutational signatures of the four subtypes, including single-base substitution (SBS) (Supplementary Fig. S17a), double-base substitution (DBS) (Supplementary Fig. S17b), and insertion–deletion (ID) mutations (Supplementary Fig. S17c). The APOBEC signature (SBS2 and SBS13) steadily increased from LPI to HPI (Supplementary Fig. S17d, e), with a similar increase observed in the frequency of tobacco-linked signatures SBS4 and ID3 (Supplementary Fig. S17f). The presence of APOBEC mutational signatures, such as SBS2 and SBS13, or tobacco smoking signatures, such as SBS4 and ID3, was consistently correlated with a poor prognosis for patients with the LPI subtype (Supplementary Figs. S18 and S19). We subsequently surveyed the distribution of 24 CNV signatures among the four subtypes (Supplementary Fig. S20a, b). Among them, CN9 had a higher mutation frequency in all four subtypes. CN9 was characterized by predominantly small segment sizes, with very few segments exceeding 40 Mb on a diploid background (Supplementary Fig. S20c). Hence, it was designated as a focal loss of heterozygosity (LOH) signature. LOH plays a crucial role in the inactivation of tumor suppressor genes during cancer development^27^. Patients with IME and HPI subtypes carrying CN9 had a poorer prognosis than those without CN9 did (Supplementary Fig. S21), which is consistent with findings from previous pancancer studies^28^. Finally, we utilized a nonnegative matrix factorization (NMF)-based method to extract structural variant (SV) signatures, combining complex and simple SVs. SVs were further classified as clustered or nonclustered, leading to a total of 32 SV types (Supplementary Fig. S22). Using these SV types, we effectively decomposed eight SV signatures labeled SV32A–SV32H (Supplementary Figs. S23 and S24). Notably, patients with the SV32E signature in LPI and IME subtypes experienced longer OS (Supplementary Fig. S25).

### Patients with LPI and IMD were more likely to benefit from TKIs

**Fig. 4:**
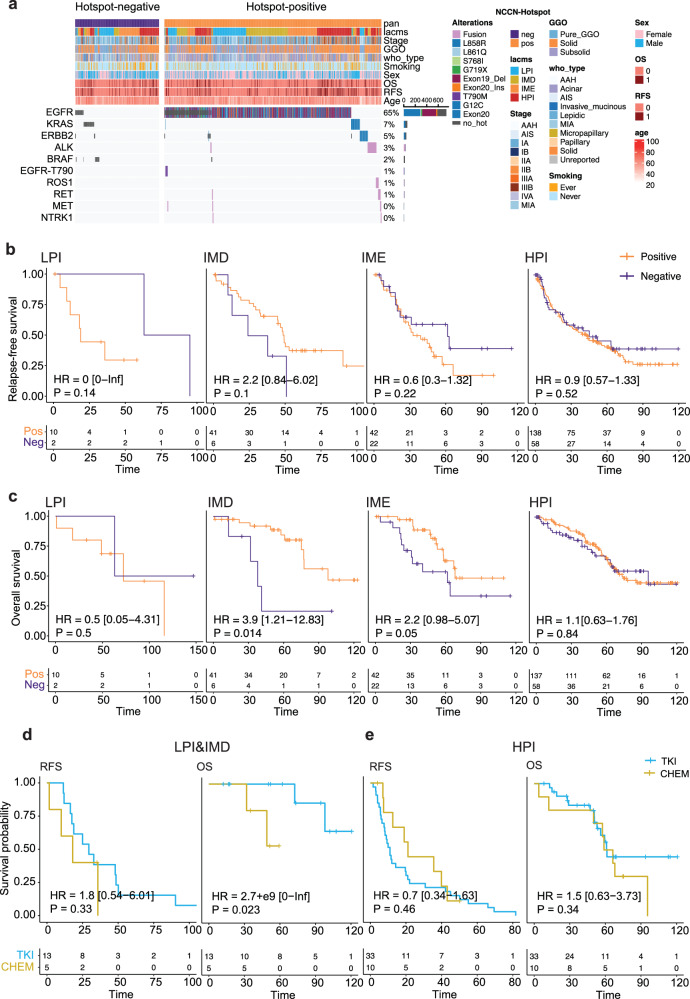
Survival outcomes associated with TKI therapy in the four identified subtypes. **a** Distribution of NCCN hotspots in the 937 LUAD samples from our cohort. **b**, **c** Relapse-free survival (**b**) and overall survival (**c**) of patients classified as positive and negative for NCCN hotspots in the LPI, IMD, IME, and HPI subtypes. **d**, **e** Comparisons of relapse-free survival and overall survival between patients with the LPI&IMD and HPI subtypes who received TKI therapy (**d**) and those who received chemotherapy (**e**).

Significant therapeutic responses are frequently observed when actionable alterations of tyrosine kinases are effectively inhibited, especially compared with patients who do not carry actionable mutations^29^. We stratified LUAD patients into hotspot-positive (*n* = 676) and hotspot-negative (*n* = 261) groups based on the presence or absence of actionable mutations outlined in the NCCN guidelines (v5.2022) (Fig. 4a). We compared the prognosis of patients in the hotspot-positive and hotspot-negative groups after excluding patients who, according to the NCCN guidelines, did not require treatment, namely, AAH, AIS, and MIA, and postoperative recurrence-free patients with pathological stage I disease, to ensure scientific rigor. The hotspot-positive group relatively more frequently consisted of nonsmoking female patients with IMD, acinar-predominant tumors (Supplementary Fig. S26a). In LPI, actionable mutations were correlated with a poor prognosis (Fig. 4b, c). These findings suggest that these mutations may have a stronger influence on disease progression, especially in the early stages. In the IMD and IME subtypes, compared with hotspot-negative patients, hotspot-positive patients had no difference in RFS but had significantly longer OS (Fig. 4b, c). This pattern was not observed in the HPI group. Moreover, in the hotspot-positive group, a notable increase in OS compared with RFS as observed in patients with the IMD and IME subtypes, whereas this difference was not observed in the hotspot-negative group (Supplementary Fig. S26b, c). These findings suggest that the IMD and IME subtypes, particularly the IMD subtype, may benefit from targeted TKI therapy.

To validate whether the IMD subtype benefited the most from TKI treatment, We compared the RFS and OS of patients with each of the four subtypes (LPI to HPI) in the LC-1000 and LC-197 cohorts who received TKI treatment or chemotherapy, both separately and combined. Our analysis revealed significantly longer OS with TKI treatment for patients with the IMD subtype than with chemotherapy (Supplementary Fig. S26d, e). Additionally, the 5-year survival rate for patients with the LPI subtype receiving TKI treatment was nearly 100% (Supplementary Fig. S26e). Compared with chemotherapy, patients with LPI and IMD clearly benefited significantly more from TKI treatment (Fig. 4d; Supplementary Table S2). In contrast, no significant difference in benefit was observed between TKI treatment and chemotherapy for patients with HPI (Fig. 4e). For patients with IME, a trend suggested that TKI treatment might be superior to chemotherapy, but the difference was not statistically significant (Supplementary Fig. S26d, e). The analysis of an external treatment cohort further confirmed that LPI and IMD patients benefit more from TKI therapy than from chemotherapy (Supplementary Fig. S27). Therefore, patients with the LPI and IMD subtypes, which are characterized by low proliferation and low immune activity, were more likely to benefit from TKI therapy than those with the HPI subtype, which exhibited a solid expression profile.

### Genomic and transcriptomic events associated with TKI resistance

**Fig. 5:**
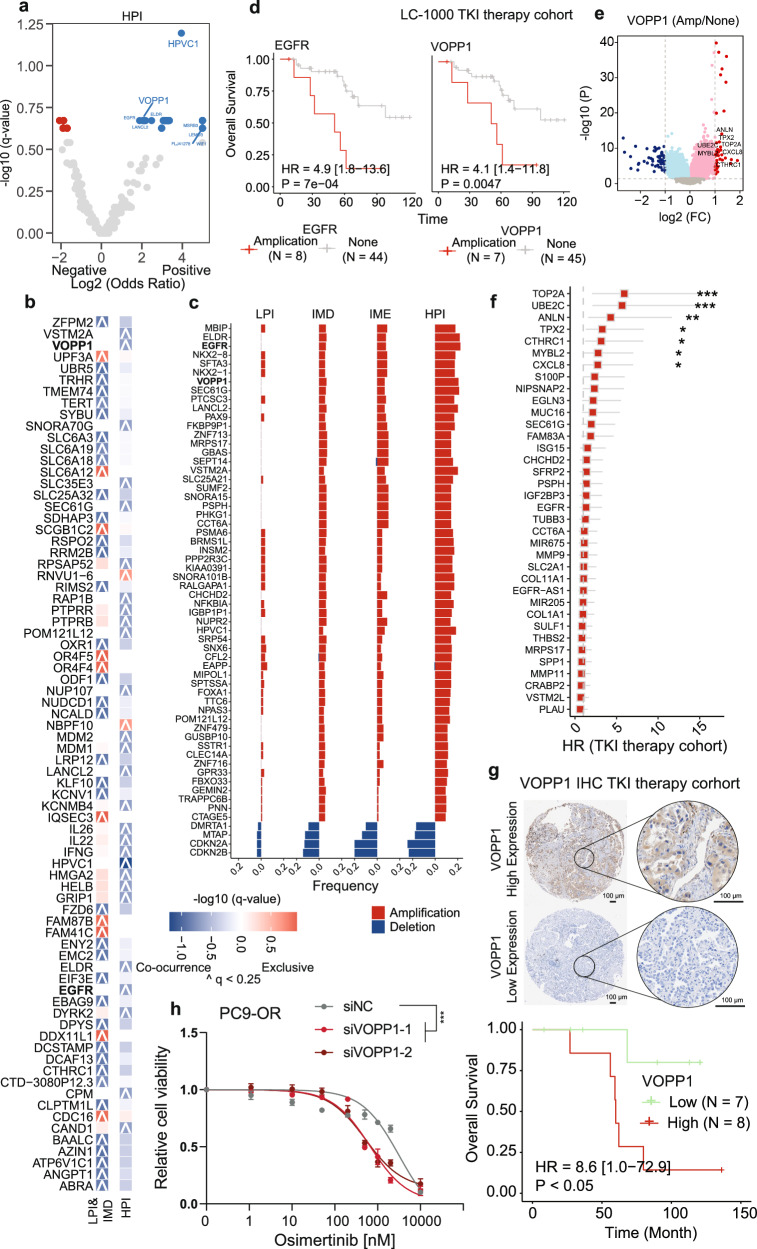
Molecular events associated with resistance to TKI therapy. **a** Co-occurrence and exclusive occurrence patterns of genes with copy number variants in hotspot-positive and hotspot-negative groups were analyzed separately for the HPI subtype. **b** Co-occurrence and exclusive occurrence of genes with copy number variants (CNVs) in NCCN hotspot-positive samples among the LPI&IMD and HPI subtypes. **c** Frequency distribution of variant occurrences among significantly different CNV genes across the four subtypes in NCCN hotspot-positive samples. **d** Overall survival was assessed for patients with EGFR and VOPP1 amplifications co-occurring with positive hotspots in the HPI subtype compared with those without genetic variants among individuals who underwent TKI therapy in the LC-1000 cohort. **e** Volcano plot displaying the distributions of *P* values and fold changes between the VOPP1 amplification group and the nonvariant group. **f** Genes whose expression was significantly upregulated with VOPP1 amplification among patients who received TKI therapy according to the univariable analysis. **g** Representative images of immunohistochemical staining for VOPP1 (top panel). Scale bars in both the main and magnified images correspond to 100 μm. (Bottom panel) Comparison of overall survival between patients with high VOPP1 expression (above the median) and low VOPP1 expression (below the median) in the TKI-treated cohort. **h** Dose–response curves of PC9-OR cells treated with various concentrations of osimertinib for 72 h after control treatment (siNC) and VOPP1 silencing (siVOPP1). ***P < 0.001.

We hypothesize that the differences in sensitivity to TKI treatment among the four subtypes, particularly between LPI/IMD and HPI, may result from concurrent genomic variations at the locus targeted by TKI treatment, which either inhibit or enhance the response to TKI therapy. Copy number variants, notably amplifications of EGFR^30^, ERBB2^31^, and MET^32,33^, have consistently been linked to TKI resistance. Consequently, we compared the co-occurrence of copy number alterations (CNAs) with TKI targets between the TKI-sensitive subtypes LPI and IMD and the TKI-resistant subtype HPI. We identified CNAs that significantly co-occurred or were mutually exclusive with actionable mutations in the LPI and IMD and HPI subtypes (Fig. 5a). EGFR, VOPP1 and HPVC1 amplifications, which occurred at chromosome 7p11.2, resulted in significant co-occurrence of hotspot mutations in the CNA gene within the TKI-resistant HPI subtype (Fig. 5a, b), whose frequency significantly increased from LPI to IMD, IME and HPI (Fig. 5c). EGFR, VOPP1, and HPVC1 amplification were able to stratify the risk for individuals who underwent TKI therapy in the LC-1000 cohort. Patients with EGFR, VOPP1, and HPVC1 amplification had worse prognoses, especially OS, than those without genetic variants (Fig. 5d; Supplementary Fig. S28a). Consistent with the findings of previous studies^30^, the amplification of EGFR, which is located on chromosome 7p11.2, remained a contributing factor to resistance to TKI therapy in our cohort. Considering the comutation patterns resembling EGFR amplification and the absence of reported associations with TKI resistance in the literature, HPVC1, despite exhibiting the most significant comutation, was excluded from the final exploration of TKI resistance because of its classification as a noncoding RNA and its low expression levels (Supplementary Fig. S28b). Consequently, VOPP1 was selected for further investigation. The amplification of VOPP1 significantly upregulated the expression of 36 genes (Fig. 5e), among which 19.4% (7/36) were associated with a poor prognosis in the TKI-treated cohort (Fig. 5f; Supplementary Fig. S28c). We further validated the association between high VOPP1 expression and a poor prognosis for patients receiving TKI therapy by retrospectively collecting FFPE samples from TKI-treated patients and performing immunohistochemical staining for VOPP1. The results confirmed that patients with high VOPP1 expression experienced significantly worse survival outcomes following TKI treatment than those with low VOPP1 expression (Fig. 5g). These findings suggest that VOPP1 amplification may contribute to increased resistance to TKI therapy. We knocked down VOPP1 in TKI-resistant PC9-OR cells to validate our hypothesis. Consistent with our expectations, dose escalation experiments with osimertinib showed that the half-maximal inhibitory concentration (IC_50_) was significantly lower in VOPP1-knockdown cells than in control cells, indicating increased sensitivity to treatment (Fig. 5h).

### Genomic and transcriptomic events associated with TKI sensitivity

**Fig. 6:**
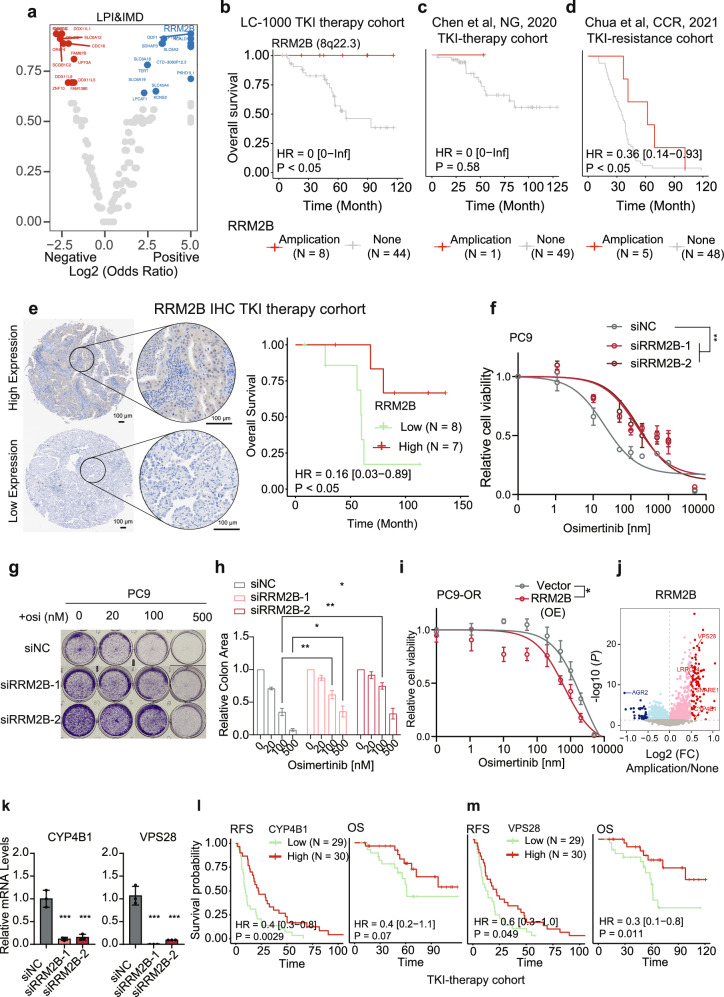
Molecular events associated with the response to TKI therapy. **a** Co-occurrence and exclusive occurrence patterns of genes with copy number variants in hotspot-positive and hotspot-negative groups were analyzed separately for the LPI and IMD subtypes. **b** Survival outcomes, including OS, were compared between patients in whom RRM2B amplification co-occurred with positive hotspots in the LPI and IMD subtypes and those without genetic variants among TKI-treated individuals in the LC-1000 cohort. **c**, **d** The OS of patients with RRM2B CNA was compared to that of the nonvariant group in the external TKI therapy (**c**) and TKI-resistant (**d**) cohorts. **e** (Left panel) Representative images of immunohistochemical staining for RRM2B. Scale bars in both the main and magnified images correspond to 100 μm. (Right panel) Comparison of overall survival between patients with high RRM2B expression (above the median) and low RRM2B expression (below the median) in the TKI therapy cohort. **f** Dose–response curves of PC9 cells treated with various concentrations of osimertinib for 72 h under negative control (siNC) and RRM2B-silenced (siRRM2B) conditions. **g**, **h** Images of representative wells (**g**) and quantification (**h**) of colony formation assays of PC9 cells treated with increasing concentrations of osimertinib under negative control (siNC) and RRM2B-silenced (siRRM2B) conditions. **i** Dose–response curves of PC9-OR cells treated with various concentrations of osimertinib for 72 h under control (vector) and RRM2B-overexpressing (RRM2B) conditions. **j** Volcano plot displaying the distributions of *P* values and fold changes between the RRM2B amplification and the nonvariant groups. **k** Relative mRNA expression levels of RRM2B-regulated genes, including CYP4B1 and VPS28, in PC9 cells under control and RRM2B-silenced conditions. **l**, **m** Comparison of OS and RFS between the high- and low-expression groups based on the median values of CYP4B1 (**l**) and VPS28 (**m**) in TKI-treated patients. *P < 0.05, **P < 0.01, and ***P < 0.001.

We also compared the co-occurrence of copy number alterations (CNAs) with TKI targets between the TKI-sensitive LPI and IMD subtypes. Conversely, RRM2B, located on chromosome 8q22.3, was found to have significant comutations among the hotspot mutations in the LPI and IMD subtypes (Fig. 6a). RRM2B exhibits a clonal copy number amplification (Supplementary Fig. S29). In TKI-treated patients from the LC-1000 cohort, individuals with RRM2B amplification had a favorable prognosis compared with those without genetic variations (Fig. 6b). Crucially, the significant observation that patients with RRM2B amplification were more likely to benefit from TKI therapy was validated in a TKI therapy cohort and a stage IV TKI-resistant cohort (Fig. 6c, d). These findings suggest that RRM2B amplification may contribute to increasing TKI treatment efficacy and reversing TKI treatment resistance. We further validated that high RRM2B expression was associated with an improved prognosis for patients receiving TKI therapy by retrospectively collecting FFPE samples from TKI-treated patients and performed immunohistochemical staining for RRM2B. The results confirmed that patients with high RRM2B expression experienced significantly better survival outcomes following TKI treatment than those with low RRM2B expression (Fig. 6e). The excellent prognosis of TKI-treated patients with RRM2B copy number amplification prompted us to investigate RRM2B. We silenced RRM2B in the PC9 cell line, which harbors an EGFR-sensitizing mutation, using two independent siRNAs to evaluate whether RRM2B affects the sensitivity to TKI treatment (Supplementary Fig. S28d). Dose escalation experiments with osimertinib revealed a significant increase in the half-maximal inhibitory concentration (IC_50_) in RRM2B-knockdown cells compared with that in control cells, indicating reduced sensitivity (Fig. 6f). Consistently, the results of the colony formation assays indicated that RRM2B-knockdown cells exhibited an enhanced colony-forming ability following osimertinib treatment (Fig. 6g, h). In contrast, RRM2B-overexpressing cells exhibited a significant decrease in the IC_50_, indicating an increased sensitivity to osimertinib (Fig. 6i; Supplementary Fig. S28e). These findings suggest that RRM2B increases osimertinib sensitivity, highlighting the potential role of RRM2B amplification in promoting a favorable therapeutic response. In RRM2B-knockdown PC9 cell lines, genes such as CYP4B1, VPS28, LRRC14, and TSNARE1 were downregulated, whereas these genes were upregulated in samples with RRM2B amplification (Fig. 6j, k; Supplementary Fig. S28f). Their expression levels in the TKI-treated cohort generally correlated with sensitivity to TKI treatment (Fig. 6l, m; Supplementary Fig. S28g, h).

We proposed that the amplification of RRM2B and VOPP1 could impact the effectiveness of TKI therapy through the modulation of downstream gene expression. Consequently, we assessed genes whose expression significantly differed between the RRM2B and VOPP1 amplification groups and the wild-type group (Supplementary Fig. S30a, b). Regarding the mechanism by which TKI sensitivity is related to gene copy numbers, we hypothesized that genes whose expression is upregulated by RRM2B copy number amplification may be linked to TKI sensitivity, whereas genes whose expression is downregulated may be linked to TKI resistance. The reverse applies to VOPP1. Therefore, we identified genes upregulated by RRM2B amplification and downregulated by VOPP1 amplification to identify genes associated with TKI sensitivity and, conversely, genes downregulated by RRM2B amplification and upregulated by VOPP1 amplification to identify those linked to TKI resistance. This analysis revealed that eight genes (AGR2, CXCL1, S100A9, MMP1, CP, CA9, XAGE1B, and MUC5B) were associated with TKI resistance, and two genes (CYP4B1 and PALM3) were associated with TKI sensitivity (Supplementary Fig. S30c, d). Notably, the expression of CYP4B1 and AGR2 changed most significantly when VOPP1 and RRM2B were amplified, respectively. In contrast to CYP4B1, AGR2 was associated with resistance to TKI therapy (Supplementary Fig. S30e).

We directly compared differentially expressed genes between hotspot-positive (potential TKI treatment benefit group) and hotspot-negative groups to further confirm the ability of RRM2B and VOPP1 to discriminate between TKI-sensitive and TKI-resistant patients. This analysis revealed seven DEGs (Supplementary Fig. S30f). Interestingly, these seven genes were significantly correlated with RRM2B or VOPP1 copy number variations (Supplementary Fig. S30g, h). CRABP2 and MUC21, genes that were highly expressed in the positive group and whose expression decreased with a VOPP1 CNA or increased with an RRM2B CNA, were strongly associated with a TKI treatment benefit. For example, elevated expression of SCGB3A1, PIGR, NASPSA, and C16orf89 was significantly associated with prolonged OS and RFS (Supplementary Fig. S30i, j). Conversely, genes whose expression was low in the positive group and whose expression increased with increasing VOPP1 copy number amplification or decreased with decreasing RRM2B copy number amplification were strongly associated with TKI resistance. For instance, elevated S100P expression was significantly associated with shorter OS and RFS (Supplementary Fig. S30i, j). These findings further indicated that RRM2B amplification may promote TKI sensitivity, whereas VOPP1 amplification may lead to TKI resistance. We further elucidated the regulatory mechanisms of VOPP1 and RRM2B in potential TKI resistance and sensitization by analyzing the genes regulated by VOPP1 and RRM2B expression in our cohort (Supplementary Fig. S31a) and comprehensively characterized the changes in gene expression induced by knockdown or overexpression in cell lines (Supplementary Fig. S31b). By integrating data from both cohorts, we identified 76 potential TKI resistance-related genes that may be regulated by VOPP1 or RRM2B, 16/76 (21.1%) of which were negatively correlated with the prognosis of patients in the TKI therapy cohort (Supplementary Fig. S31c, d). Additionally, 49 potential TKI sensitivity-related genes were identified, with 4/49 (8.2%) exhibiting a positive correlation with the prognosis of patients in the TKI therapy cohort (Supplementary Fig. S31e, f). The pathway enrichment analysis revealed that siVOPP1 treatment reduced the activity of the TKI resistance-associated PI3K–AKT–MTOR and WNT pathways, whereas RRM2B overexpression decreased the activity of the EMT and WNT pathways, which are associated with TKI resistance (Supplementary Fig. S31g, h). These findings suggest that VOPP1 may promote TKI resistance by potentially regulating the PI3K–AKT–MTOR and WNT pathways, whereas RRM2B may increase TKI sensitivity by reducing the activity of the EMT and WNT pathways. In addition to the genes regulated by VOPP1 and RRM2B, we systematically investigated the expression of noncoding RNAs in relation to TKI therapy and identified potential TKI-associated noncoding RNA genes through a prognostic correlation analysis (Supplementary Fig. S32).

### Patients with IME are more likely to benefit from immunotherapy

**Fig. 7:**
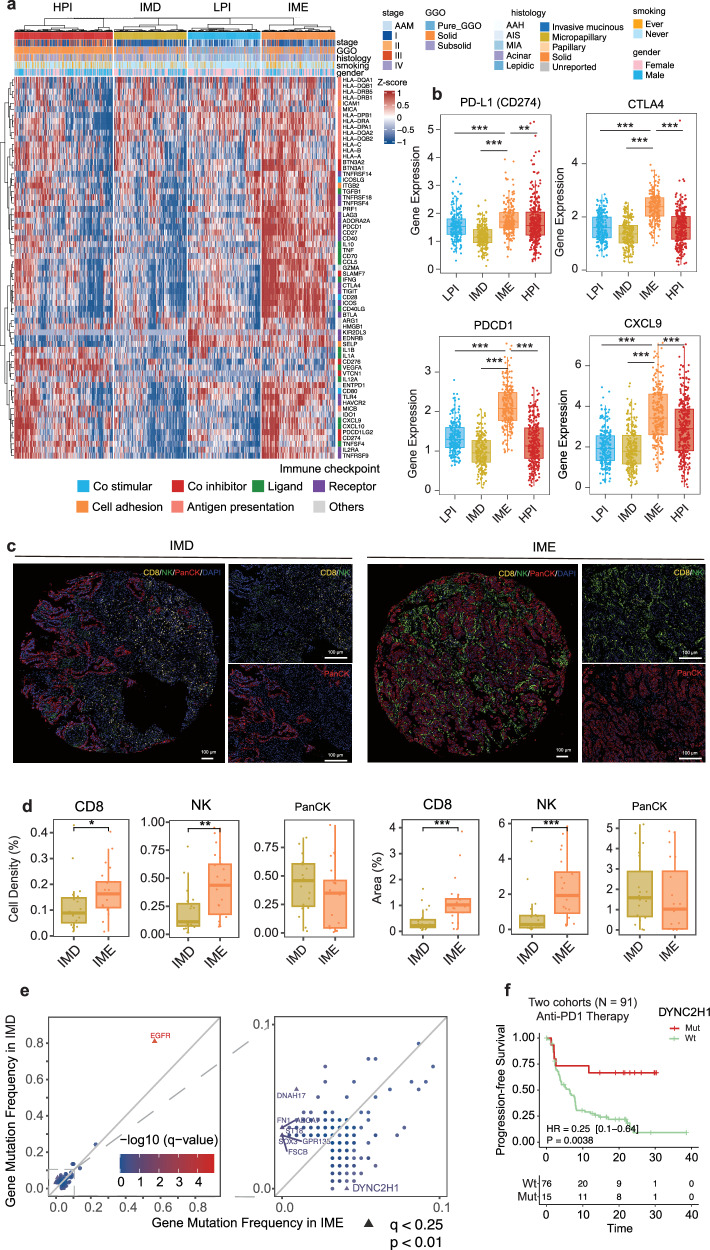
Distinct immunological profiles of the four subtypes. **a** Heatmap depicting the distribution of immune checkpoint genes among the four subtypes in the LC-1000 cohort. **b** Boxplots illustrating the expression levels of immune checkpoint genes, such as PD-L1, CTLA4, PDCD1 and CXCL9, across the four subtypes in the LC-1000 cohort. **c** Representative images of multiplex immunofluorescence staining for CD8^+^NK^+^PanCK^+^ cells in the IMD (left panel) and IME (right panel) subtypes. CD8^+^ T, NK and tumor cells were detected using CD8, CD16 and PanCK staining. DAPI was used to identify nucleated cells. Scale bars in both the main and magnified images correspond to 100 μm. **d** Boxplot illustrating the comparison of CD8^+^, NK and PanCK cell density (%) and area (%) between the IMD and IME subtypes. **e** Comparison and distribution of gene mutation frequencies between IMD (characterized by the coldest immune cell profile) and IME (characterized by the hottest immune cell profile). **f** Progression-free survival was analyzed for mutant and wild-type DYNC2H1, whose frequency was significantly higher in IME than in IMD, among the 91 patients who received immunotherapy. *P < 0.05, **P < 0.01, and ***P < 0.001.

The robust immune cell activity observed in the IME subtype indicated that patients in this subgroup may benefit from immunotherapy. Thus, we conducted a comprehensive assessment of immunomodulators based on previously identified inhibitory receptor genes and their corresponding ligands^34^. This analysis indicated that IME patients with higher T-cell infiltration also had higher expression levels of immune checkpoint genes (Fig. 7a). PD-L1, CTLA4, PDCD1, and CXCL9 have been shown to be strongly associated with the immunotherapeutic response^35,36^. As anticipated, we found that the expression of these four genes in the IME subtype was significantly higher than that in the other three subtypes (Fig. 7b). We collected transcriptomic and proteomic data from CPTAC to determine whether the expression of these inhibitory receptor proteins is consistent with their mRNA expression. First, utilizing the gene expression patterns for the previously mentioned 20 features, we stratified the CPTAC cohort into four subtypes: LPI, IMD, IME, and HPI (Supplementary Fig. S33a). We subsequently examined the overall expression levels of immune checkpoint genes (Supplementary Fig. S33b) and proteins (Supplementary Fig. S33c) in the CPTAC cohort. Notably, PD-L1 (CD274) and CXCL9 exhibited similar gene (Supplementary Fig. S34a) and protein (Supplementary Fig. S34b) expression patterns, with a significant upregulation observed in IME among the four subtypes in the CPTAC cohort. Immunofluorescence staining showed a lower abundance of tumor-killing CD8⁺ T cells and NK cells in the IMD subtype than in the IME subtype (Fig. 7c, d). Interestingly, in addition to their reduced numbers, these immune cells in the IMD tumors were located farther from the tumor cells (Fig. 7c; Supplementary Fig. S34c), suggesting impaired immune cell infiltration and limited access to the tumor core. This spatial exclusion may prevent cytotoxic cells from effectively targeting tumor cells, potentially contributing to the poorer response to immunotherapy observed in IMD patients than in those with the IME subtype (Supplementary Fig. S34d).

In contrast to IME, IMD had the lowest immune cell activity and the lowest immune checkpoint protein expression levels across all four subtypes. The most pronounced difference in immunotherapy response-related molecular events was observed between the IME and IMD subtypes. Hence, the molecular differences between the IME and IMD subtypes may also be correlated with the immunotherapy response. Furthermore, our analysis revealed that DYNC2H1 mutations were significantly more frequent in IME than in IMD (Fig. 7e). Consequently, we speculated that the presence of DYNC2H1 mutations might indicate a potential benefit from immunotherapy. We obtained gene mutation and prognostic data from two published immunotherapy cohorts with LUAD to substantiate our hypothesis. Encouragingly, patients with DYNC2H1 mutations experienced significantly longer progression-free survival (PFS) than patients expressing wild-type DYNC2H1 in the immunotherapy cohort, validating our hypothesis (Fig. 7f; Supplementary Fig. S34e). These results further reinforced the hypothesis that patients with IME may indeed derive benefits from immunotherapy.

## Discussion

A comprehensive understanding of LUAD biology at the systems level is essential for achieving precise molecular subtyping and predicting the treatment response. Here, we present a large-scale clinical, genomic, and transcriptomic characterization of 1008 tumor samples from Chinese patients with LUAD. The detailed radiological, pathological, and omics data allowed us to track LUAD development and identify four subtypes with distinct molecular characteristics, prognose,s and treatment outcomes. Based on these four subtypes, we identified key driver pathways and potentially novel therapeutic targets. The large number of LUAD patients and comprehensive prospective longitudinal clinical and therapeutic data from this cohort distinguish it from other published cohorts analyzed in LUAD omics studies^14,15^.

Numerous studies have focused on the molecular subtyping of LUAD and have investigated variations in single genes^8–12^, gene expression^14,37^, protein expression^15,16^, and copy number^38^. Both our previous research and other studies revealed distinct molecular characteristics for clinical phenotypes such as GGO, lepidic adenocarcinoma, adenocarcinoma in situ/minimally invasive adenocarcinoma (AIS/MIA), solid tumors, and metastases^19^. Additionally, the TME is widely recognized as closely associated with tumor progression and has significant potential to inform clinical treatment decisions^22^. However, studies that integrate both clinical (macroscopic) and TME (microscopic) features into a single model for comprehensive molecular subtyping are lacking. In this study, key radiological characteristics, such as ground-glass opacity (indicating inertia) identified using radiology, the lepidic subtype (also reflecting inertia), and the solid and micropapillary subtypes (indicating progression), as well as recurrent or metastatic features (also reflecting progression), were identified by pathology. In the TME dimension, tumor, stromal, and immune cell characteristics were extracted. Altogether, we chose 20 clinical and TME features and developed signatures based on transcriptomic data intended for molecular subtyping.

By performing unsupervised clustering of the 20 clinical and TME features, we classified LUAD samples into four subtypes, i.e., LPI, IMD, IME, and HPI. Patients with the LPI subtype had the most favorable outcomes in terms of OS and RFS, likely because of their less aggressive tumor characteristics, notably the predominantly GGO appearance and slower growth, as observed pathologically with a lower cell density and the maintenance of a lepidic state within GGO nodules^19,39^. The EGFR mutation frequency in the IMD group was the highest among the four subtypes. The increase in EGFR mutation was associated with a lymphocyte-depleted phenotype that was particularly characterized by the absence of CD8^+^ T-cell infiltration in tumors^40,41^. Studies have shown that compared with tumors lacking CD8^+^ T-cell infiltration, tumors that have infiltrated and contain CD8^+^ T cells exhibit superior responses to immune checkpoint inhibitor (ICI) therapy^42^. Hence, the overexpression of mutant EGFR may account for the immune-desert phenotype observed in IMD and the ineffectiveness of ICI therapy^34,43^. The IME subtype exhibited increased immune activity and tumor-killing ability, possibly because it had the lowest prevalence of EGFR mutations among the four subtypes (Fig. 2b; Supplementary Fig. S14). Interestingly, most patients with IME were diagnosed with the PI subtype, with increased immune cell infiltration described in TCGA cohort, while IMD corresponded to the EGFR signaling overactive subtype TRU and immune-cold subtype PP^14,44^ (Supplementary Fig. S10). The HPI subtype demonstrated the highest risk of recurrence and metastasis, accompanied by the most malignant clinical phenotype and swiftest tumor progression, including cell proliferation, matrix remodeling, and CAFs. Cell proliferation-related pathways were also significantly upregulated in the HPI group (Fig. 2d). Notably, our four-subtype classification was validated in the LC-197 and TCGA cohorts, confirming its robustness and applicability across different datasets (Fig. 3; Supplementary Fig. S11).

Based on molecular characteristics of the four subtypes, our comprehensive analysis identified potentially more suitable treatment strategies for LUAD subtypes, offering a roadmap for future investigations. First, patients categorized as having the LPI subtype typically have a promising prognosis. Even among the minority who experienced relapse, their OS remained excellent, often with effective treatment using TKIs or chemotherapy (Fig. 4d). With the exception of specific genomic events such as EGFR mutations, the IMD subtype displayed minimal activity in terms of tumor proliferation, metabolism, and immune-related biological processes. Tumor cells with these characteristics seem to be particularly responsive to targeted therapy (Fig. 4d). Compared with the other subtypes, the IME subtype displayed active immune cell infiltration, particularly by CD8^+^ T cells, along with notably elevated expression levels of immune checkpoint markers such as PD-L1, CTLA4, and CXCL9. These characteristics indicated the potential benefit of immunotherapy for patients classified under this subtype (Fig. 7). Finally, compared with patients with IMD, patients with DYNC2H1 mutation, whose expression was significantly upregulated in IME, were more likely to benefit from ICIs, which is consistent with the findings of a previous study^45^. This line of evidence further supported the hypothesis of potential benefits of immunotherapy for IME. In contrast, the HPI subtype, which is known for increased proliferation and metabolism, presents obstacles to therapeutic effectiveness. We speculated that this result may be attributed to complex clonal evolution in advanced LUAD, where clones predominantly governed by other driver genes replace those dominated by actionable mutations. Consequently, targeting actionable mutations becomes challenging, potentially explaining the limited efficacy of TKI therapy against the HPI subtype (Fig. 4d)^46^. With respect to immunotherapy, perhaps the glycolysis pathway (Fig. 2d), which has been proven to aid in immune escape in various tumors^26^, made HPI potentially insensitive. Overall, LPI and IMD were suitable for targeted therapy, while IME may benefit from immunotherapy because of the high DYNC2H1 mutation frequency, but HPI was relatively insensitive to current therapies.

Our study revealed that VOPP1, located on chromosome 7p11.2, was linked to treatment resistance (Fig. 5d). Notably, both VOPP1 and EGFR are located on chromosome 7p11.2, with an EGFR CNA previously reported to be associated with TKI treatment resistance^47^. Although case reports have indicated the potential co-occurrence of VOPP1 and EGFR fusion genes with T790M^48^, the relationship between VOPP1 amplification and TKI resistance has not yet been thoroughly investigated. Several genes, including SCGB3A1, PIGR, NASPA, C16orf89, and S100P, are strongly linked to TKI therapeutic targets (Supplementary Fig. S30g). Previous studies also revealed that S100P is highly expressed in the TKI-resistant cell line PC-9/ER compared with that in the normal cell line PC-9^49^. In addition, the S100P mRNA level correlated with the activation status of the PI3K/AKT pathway^50^, which is a classic pathway involved in promoting therapeutic resistance in various cancers^51^. These findings suggest that VOPP1 may induce resistance to TKI treatment in tumor cells by upregulating S100P expression, representing one potential mechanism. These molecular events and potential mechanisms will contribute to identifying a more refined subset of individuals who may benefit from TKIs and offer a theoretical foundation for addressing resistance to TKI treatment.

Although virtually none of the available therapies proved effective at treating HPI patients, individuals receiving TKIs with an amplification of the RRM2B gene, which is located on chromosome 8q22.3, experienced a survival of more than 10 years (Fig. 6b). Furthermore, in stage IV TKI-resistant patients with distant metastases, those with RRM2B amplification had a remarkable OS of three years (Fig. 6d). In contrast, the three-year OS of patients with advanced LUAD treated with osimertinib was approximately 50%^52^, underscoring the potential significance of RRM2B amplification in patients with advanced-stage tumors with TKI resistance. RRM2B can increase cellular responses to decitabine, and decitabine can reverse gefitinib resistance in LUAD^53^. Furthermore, our results provided insights into the downstream effects of RRM2B amplification, which may lead to the upregulation of CYP4B1 and VPS28 expression and the downregulation of AGR2 expression (Fig. 6k; Supplementary Fig. S30d). AGR2 is also a pro-oncogenic protein that stimulates the expression of the EGFR ligand amphiregulin and regulates p53 signaling, suggesting its potential relationship with RRM2B, which encodes the p53 ligand p53R2^54^. Previous studies have linked increased expression of AGR2 to TKI-induced drug resistance through a decrease in tumor cell apoptosis^55^ in various human cancer cell lines, such as lung^56^, esophageal^57^, liver^55^, prostate^58^, breast^59^, and pancreatic^60^ cancer. Moreover, AGR2 knockdown can resensitize TKI-resistant cells to TKI therapy^61^. AGR2 switched cancer cells from the TKI-sensitive type to the TKI-resistant type, probably by modulating endoplasmic reticulum homeostasis^55^. Therefore, the amplification of RRM2B might increase the sensitivity of tumor cells to TKIs through the downregulation of AGR2 expression.

In summary, we conducted a comprehensive analysis of the omics and clinical data from LUAD patients, categorizing them into four subtypes characterized by distinct molecular profiles and clinical implications. Patients classified with the LPI subtype generally present with tumors at very early stages and exhibit a highly favorable prognosis. IMD signifies a cold tumor with the highest frequency of EGFR mutations, and the LPI and IMD subtypes significantly benefit from TKI treatment. The IME subtype represents hot tumors and may benefit from immunotherapy, as supported by the high expression of PD-L1 and CTLA4, as well as increased immune cell infiltration. However, patients with the HPI subtype may not benefit from current therapies, yet amplifications in RRM2B and high expression of CYP4B1 may serve as potential sensitizers to TKI therapy. Thus, even TKI-resistant patients may benefit from TKI therapy if these targets are present. Overall, the clinical treatment recommendations and molecular features of the four subtypes can complement traditional clinical features, aiding in determining patient outcomes and facilitating personalized therapies based on individual risk profiles.

## Materials and Methods

### Lung adenocarcinoma cohort

The LC-1000 cohort, consisting of 1008 LUAD cases, originates from the main paper^25^. For a comprehensive understanding of the clinical characteristics within the cohort, please refer to the main paper. Comprehensive clinical information, including pathology, radiology, and prognosis, was obtained for patients in the LUAD cohort. In this investigation, molecular features were extensively explored using pathological and radiological information from the LC-1000 cohort. Molecular subtyping models were subsequently constructed to elucidate the underlying heterogeneity within LUAD. The validation cohort consisted of LC-197 LUAD samples from our previously published studies^62^, providing comprehensive pathological, imaging, and prognostic information to validate the results of molecular subtyping.

### The datasets utilized in this study were obtained from published sources

The two single-cell datasets, GSE123904^63^ and GSE131907^64^, were sourced from two published studies. Count matrices for cell and gene expression were obtained directly from the GEO database for both datasets. The RNA-seq gene expression matrices for TCGA^15^ and CPTAC^15^ LUAD cohorts, along with their associated clinical information, were acquired from the Genomic Data Commons (GDC) database^65^. Additionally, the proteomic data from CPTAC were downloaded directly from the Clinical Proteomic Tumor Analysis Consortium (CPTAC) database. During the processing of RNA-seq data, a single gene symbol was found to correspond to multiple Ensemble IDs. We addressed this issue by opting for the Ensemble ID associated with the highest signal strength to represent the expression level of the respective gene.

The RNA-seq and CNV datasets related to TKI treatment resistance were acquired from the study by Chua et al.^66^. We retrieved the RNA-seq and CNA matrices, along with the corresponding clinical information, to reanalyze the data. The genomic data related to immunotherapy for LUAD for this study were sourced from two studies, Rizvi et al.^67^ and Hellmann^68^. Gene mutations, along with their corresponding clinical information, were extracted from the cBioPortal database^69,70^. These datasets were subsequently reanalyzed to validate and reinforce the conclusions drawn in this study.

### Whole-genome and transcriptome data

The whole-genome and transcriptome data utilized in this study originated primarily from the main paper. The in-depth analysis of FASTA data for whole-genome and whole-transcriptome sequencing is extensively described in the main paper. This study directly employed the mutation matrix, copy number variant segment matrix, structural variation (SV), gene expression, and fusion gene matrices from the main paper for subsequent analyses.

### Analysis of single-cell datasets

Single-cell gene and cell filtering were performed using the Scanpy (v1.9.6) package^71^. The analysis utilized counts per gene per cell as the input. The filtering process included the following steps: (1) genes were retained if they were expressed in at least 3 cells, and cells were retained if they expressed at least 200 genes; (2) samples with more than 5% counts for mitochondrial genes were filtered; and (3) n_genes_by_counts < 2500 were filtered.

For the screen of cell type-specific genes, GSE123904^63^ included 8 LUAD samples with 15,430 cells and 19,150 genes, whereas GSE131907^64^ included 11 LAUD samples with 29,749 cells and 24,321 genes. Utilizing LEIDEN combined with UMAP clustering with a RESOLUTION = 0.3, all LUAD samples from GSE123904 and GSE131907 were categorized into 12 and 15 subpopulations, respectively. Each cellular subpopulation was compared with the remaining subpopulations, and genes specifically expressed in each cellular subpopulation were obtained by setting a threshold of log_2_FC ≥ 1 and a *P* value < 0.05. The marker genes employed for cellular annotation in LUAD are based on established references from published literature^72,73^. T cells, CD3D, CD2; CD8^+^ T cells, CD8A, CD8B; CD4^+^ T cells, CD4, CD3E; regulatory T cells (Tregs), CTLA4, FOXP3; natural killer cells, NKG7, KLRD1; B cells, CD19, CD79A; plasma cells, MZB1, CD38; neutrophils, FCGR3B, CSF3R; macrophages, AIF1, CD68; mast cells, MS4A2, CPA3; cancer-associated fibroblasts (CAFs), COL1A2, CALD1; endothelial cells, VWF, IFI27; and epithelial cells, SFTPC, AGER.

### Filtering of radiological and pathological features and TME marker genes

A GGO/lepidic signature, representing the inert state of LUAD, was developed. T1-4N0M0 samples were chosen from the main paper, and RNA-seq analyses of differentially expressed genes were performed by comparing samples with GGO and lepidic components to other samples. A set of 22 genes with high GGO/lepidic-specific expression was obtained using the criteria of log2FC ≥ 1 and *P* value < 0.05. The GGO/lepidic signature was subsequently constructed based on these identified genes.

A solid/micropapillary signature, representing a cellular signature phenotype indicative of the relative malignancy of lung glands, was constructed. T1-4N0M0 patients were selected from the main paper. The DEGs in samples with a solid or micropapillary-dominant phenotype were compared with those in other samples. The solid/micropapillary-specific highly expressed genes were obtained by setting the criteria of log2FC ≥ 1 and adjusted *P* value < 0.05. A total of 32 genes were initially identified, of which 11 histone-related genes not reported to be related to tumor progression were excluded. The remaining 21 genes were used to construct the solid/micropapillary signature.

A metastasis-related signature was constructed, and a LUAD metastasis-related signature was constructed by considering differences in brain metastasis, bone metastasis, and lymph node metastasis and gene expression between samples from patients in whom metastasis occurred within 1 year and samples from recurrence-free patients (those with no recurrence for more than 5 years). These genes were not present in the solid/micropapillary signature. A total of 31 highly expressed genes associated with site-specific metastasis or very early metastasis of lung adenocarcinoma were obtained by setting the criteria of log2FC ≥ 1 and *P* < 0.05. The final metastatic signature was constructed based on these 31 metastasis-related genes.

The initial step in the construction of a tumor microenvironment signature involved incorporating the marker genes obtained with seven commonly used methods for calculating cell scores in the tumor microenvironment. These methods include Cibersort^74^, MCP-counter^75^, EPIC^76^, quanTIseq^77^, X-cell^78^, Fges^79^, and Kassandra^80^. The second step involved incorporating genes that were specifically expressed in all types of cells within the single-cell dataset. Marker genes used for CD8^+^ T cells, CD4^+^ T cells, B cells, Tregs, NK cells, macrophages, CAFs, endothelial cells, and neutrophils were selected based on their presence in at least 4 of 9 sources, which included 7 computational methods and 2 single-cell datasets. Due to the limited literature available for calculating T helper cells, M1 macrophages, and dendritic cells, genes that appeared in at least two publications were selected. As only one paper investigated myeloid cells, tumor proliferation rate, matrix remodeling, and angiogenesis, genes from that paper were selected. The marker genes for the epithelial–mesenchymal transition (EMT) signature were referenced from 84 EMT signature-related papers^81^. The final set of genes selected consisted of those that appeared in at least 1/5 of the literature. Integration of published methods and single-cell cohorts was performed to identify cell-type-specific genes expressed within the tumor microenvironment. A summary of all the genes is provided in Supplementary Table S3.

### Unsupervised consensus clustering by applying 20 radiological, pathological, and TME signatures

The determination of the complex molecular subtypes of LUAD was performed using a comprehensive approach that integrated pathological, radiological, and tumor microenvironmental features. The ssGSEA algorithm^82,83^, in combination with marker genes associated with radiological, pathological, and the tumor microenvironment (TME) features, was employed to compute scores for 20 signatures related to radio-pathology and the TME. Using the 20 features, molecular subtyping was performed using the unsupervised clustering method PAM. Unsupervised clustering was executed through ConsensusClusterPlus (v1.58.0)^84^ with the parameters reps = 1000, pItem = 0.8, and pFeature = 1. We assessed 2–10 different subtypes by setting maxK = 10 to identify the optimal number of subtypes. After 2–10 potential subtypes were evaluated, four molecular subtypes of LUAD were defined by integrating biological significance, clinical relevance, and the principles of maximizing intergroup differences while minimizing intragroup heterogeneity‌

### Definitions of positive and negative NCCN hotspots

NCCN hotspot positivity and negativity were identified using the NCCN (v5.2022). We characterized hotspot-positive patients as having mutations at any of the following locations (Supplementary Table S4): EGFR (exon 18 p.G719, exon 19 del, exon 20 insertion, exon 20 p.S768I, exon 21 p.L858R, p.L861Q), KRAS (p.G12C), BRAF (p.V600E), ERBB2 (exon 20). Patients with gene fusions involving any of the following were also classified as hotspot-positive: ALK, ROS1, MET, RET. NCCN positivity comprises therapeutic target groups, whereas negativity lacks therapeutic target groups.

### Identification of gene copy number variations and HLA-LOH

BAM files generated from whole-genome sequencing were processed using the ASCAT algorithm^85^ (encapsulated in ascatNgs^86^) to identify chromosomal CNVs, yielding per-sample, per-chromosome CNV segment files. These segment files served as inputs for GISTIC2 (v2.0.23)^87^ to determine CNVs at the gene level for each sample. GISTIC2 parameters were configured as follows: ta = 0.25, td = 0.25, qvt = 0.25, cap = 1.5, brlen = 0.5, conf = 0.95, armpeel = 1, broad = 1, and savegene = 1. Gene-level deletions and amplifications were defined as thresholds of −2 and 2, respectively. Subsequently, co-occurrence and mutual exclusivity analyses were performed to identify hotspot CNV genes with frequent comutations, while analyses of associations with the clinical phenotype and survival were employed to pinpoint prognostic CNV genes. Battenberg^88^ software was used to analyze the clonal or subclonal status of the CNV.

HLA loss of heterozygosity (LOH) was identified using POLYSOLVER (v1.0.0)^89^ in conjunction with the LOHHLA algorithm^90^. Specifically, POLYSOLVER was first employed to perform HLA typing for each sample, followed by implementation of the LOHHLA algorithm to assess whether HLA LOH occurred by evaluating discrepancies between HLA haplotypes.

### Somatic mutation, copy number variation, and structural variation signatures

The activity matrix for the SBS, DBS, and ID signatures was sourced from the primary main paper. In this paper, the SigProfilerAssignment^91^ assignment function was extensively used to directly allocate known cosmic (3.3) SBS, DBS, and ID signatures to each sample and mutation. In our study, we conducted a comprehensive reanalysis of the SBS, DBS, and ID signatures and explored their characteristics in diverse molecular subtypes of LUAD. Additionally, we examined the correlation between these signatures and the prognosis of patients with different molecular subtypes of LUAD.

Similar to the SNV analysis, CNV signatures were analyzed using SigProfilerAssignment^91^. CNV signatures were classified by combining heterozygous deletion status, total copy number status, and fragment length. The COSMIC (v3.0) CNV signature set includes 24 of these signatures. Using these COSMIC-based 24 CNV signatures, we decomposed the CNVs in each sample through the analyzer function in SigProfilerExtractor, generating frequencies and counts for each signature across all the samples.

Structural variations (SVs) encompass various types of genomic alterations, including large deletions, tandem duplications, inversions, and translocations. These SV types were further categorized by size into five ranges: 1–10 Kb, 10–100 Kb, 100 Kb–1 Mb, 1 Mb–10 Mb, and events larger than 10 Mb. Additionally, SVs were distinguished as clustered or nonclustered based on the distance between neighboring SVs, resulting in a total of 32 SV types. By utilizing the NMF function of sigProfilerExtractor^92^ (v1.1.21), the features of these 32 SV types across all the samples were analyzed. Through the evaluation of 1–30 signatures, the optimal signatures were determined based on mean sample cosine distance and average stability. The distribution and prognostic relevance of the structural variation (SV) signature were extensively examined across diverse molecular subtypes of lung adenocarcinoma.

### Differential gene expression and mutation analyses

The analysis of differentially expressed genes between the two groups was conducted using the limma^93^ package (v3.50.0). The criteria for identifying differentially expressed genes were set at *P* < 0.05 and |log2(fold change)| ≥ 1. Differences in genomic events between two or more groups were detected using Fisher’s exact test (*P* < 0.05). The differences in gene mutation frequency between the two groups were directly compared using the mafCompare function in the R package maftools (v2.10.05)^94^.

### ssGSEA and predictions of T-cell exclusion and the immunotherapy response

The hallmark gene sets (v7.5), which were defined to encompass information on biological functions, were sourced from the GSEA database (https://www.gsea-msigdb.org/gsea/index.jsp). The enrichment scores for each hallmark gene in every sample were computed using ssGSEA functions implemented in the R package GSVA (v1.42.0)^95^. Using the log-transformed FPKM matrix from the LC-1000 cohort as the input, T-cell exclusion and the immunotherapy response were predicted using the tumor immune dysfunction and exclusion (TIDE) algorithm on a web-based platform^96^.

### Survival analysis

The differences in survival between the two groups were assessed using the log-rank test, and the hazard ratio was calculated using the Cox proportional hazards regression model. In the LC-1000, LC-197, TCGA, and TKI-resistant cohorts, survival analyses focused primarily on comparing overall survival (OS) and relapse-free survival (RFS). In the immunotherapy cohorts described by Rizvi et al.^67^ and Hellmann et al.^68^, survival analyses compared progression-free survival (PFS) between the two groups. Survival and Cox analyses were performed utilizing the R packages “survival” (v3.2–13) and “survminer” (0.4.9). The assessment of both overall survival (OS) and relapse-free survival (RFS) involved the application of the Kaplan–Meier survival analysis, complemented by the log-rank test. Survival analyses were performed between the hotspot-positive and hotspot-negative groups, excluding patients in the AIS/MIA stage and stage I without relapse who did not receive adjuvant therapy postoperatively.

### Cell culture and treatment

The PC9 cell line (RRID: CVCL_B260), sourced from Nanjing Cobioer Biotechnology Co., Ltd., was cultured in RPMI 1640 medium (Invitrogen) supplemented with 10% fetal bovine serum (FBS). The cells were maintained at 37 °C in a humidified atmosphere with 5% CO₂. Routine screening for mycoplasma contamination was conducted using PCR with specific primers, and cell line authentication was performed by the supplier through STR profiling.

PC9 cells with acquired resistance to osimertinib were generated as previously described^97^. Briefly, PC9 cells were treated with increasing doses in a stepwise manner until the cells tolerated osimertinib. PC9 cell lines with acquired resistance to osimertinib were referred to as PC9-OR cells.

Small interfering RNAs (siRNAs) were transfected into cells at a final concentration of 20 nmol/L in 6-well plates using Lipofectamine RNAiMAX (Thermo Fisher Scientific) according to the manufacturer’s guidelines to knock out the *VOPP1* and *RRM2B* genes. The cells were harvested 48 h after transfection for RNA analysis and phenotypic assays. The siRNA sequences are listed in Supplementary Table S5.

Lentiviral plasmids expressing FLAG-tagged human RRM2B (NM_015713) were cloned and inserted into the pL-CMV-ccdB-puro vector (Tsingke, Cat. No. PDS271) as previously described.

### Cell viability assay

Cell viability was assessed using a Cell Counting Kit-8 (CCK-8; Meilunbio). Tumor cells were plated in 96-well plates at a density of 3000 cells/100 μL per well in RPMI 1640 medium supplemented with 10% FBS. Compounds were added the following day, and after a 72-h incubation, 10% (volume/volume) CCK-8 solution was added to each well. After a 1-hour incubation, the absorbance was measured at 450 nm using a BioTek microplate reader. Relative cell viability was calculated by normalizing the absorbance of treated wells to that of vehicle-treated controls.

### Colony formation assay

Tumor cells were seeded in 12-well plates at a density of 5000 cells per well. On the second day, the cells were treated with the respective compounds, and the medium was changed every three days to include fresh compounds. After 10–12 days of treatment, the colonies were fixed with 4% formaldehyde and stained with 0.25% crystal violet. Images of the colonies were captured with a digital camera, and their areas were quantified using ImageJ software (NIH; RRID: SCR_003070). The relative colony numbers were normalized to those of the vehicle-treated group.

### Quantitative Real-Time PCR

Total RNA was extracted using TRI reagent (Sigma) according to the manufacturer’s instructions. Reverse transcription (RT) was performed with 1 μg of RNA using the Goldenstar RT6 cDNA Synthesis Kit Ver. 2 (TsingKe Biological Technology), which includes a genomic DNA removal reagent. Quantitative PCR (qPCR) was conducted using AceQ qPCR SYBR Green Master Mix (Vazyme) on an ABI Step One Plus system (Applied Biosystems) in accordance with the manufacturer’s protocol. The specificity of amplification was confirmed through agarose gel electrophoresis and a melting curve analysis. Each sample was tested in triplicate. The ΔΔCt method was employed to quantify the qPCR data. The sequences of the primers are provided in Supplementary Table S5.

### RNA isolation, library preparation, and sequencing of cell lines

Total RNA was isolated with VeZol reagent (Vazyme). After trypsin digestion, 5 × 10⁶ cells from four cell lines, namely, the siVOPP1, paired control, RRM2B-OE and paired control lines, were collected and lysed with 1 mL of VeZol reagent for 5 min at room temperature. Afterward, 200 μL of chloroform was added, followed by vigorous shaking for 15 s and an incubation for 5 min. After centrifugation at 12,000× *g* for 15 min at 4 °C, the aqueous phase (∼500 μL) was transferred to a new RNase-free tube. RNA was precipitated with an equal volume of isopropanol, incubated for 10 min, and centrifuged at 12,000× *g* for 10 min at 4 °C. The pellet was subsequently washed with 1 mL of 75% ethanol (prepared with RNase-free ddH₂O), centrifuged again, and air-dried briefly. The RNA was dissolved in 20 μL of RNase-free ddH₂O. Quality was assessed using a NanoDrop (Thermo Fisher Scientific, USA) and Agilent 2100 Bioanalyzer (Agilent Technologies, Santa Clara, CA, USA). Transcriptome libraries were prepared using the VAHTS Universal V6 RNA-seq Library Prep Kit according to the manufacturer’s instructions. Libraries were pooled based on the effective concentration and then phosphorylated and circularized. DNA nanoballs were generated via loop amplification and sequenced using a DNBSEQ-T7 flow cell. Gene expression was quantified using the HISAT2 and featureCounts pipeline. The sequences were aligned to the reference genome using HISAT2 (v2.21), and the reads mapped to each gene were counted using featureCounts (v2.0.6). The fragments per kilobase million (FPKM) values for each gene were then calculated based on gene length and the number of reads mapped to the corresponding gene. Biological and functional analyses were subsequently conducted based on the FPKM values.

### Immunohistochemical staining

LUAD FFPE sections were heated at 65 °C for 2 h, deparaffinized twice with xylene for 15 min each, and rehydrated through a graded ethanol series (100%, 85%, and 75%) and distilled H_2_O_2_ for 5 min each. Antigen retrieval was conducted in citrate buffer using microwave irradiation: medium power for 8 min until boiling, 8 min standing at sub-boiling temperature, and 7 min at medium-low power, avoiding evaporation. After natural cooling, the sections were washed three times with PBS. Endogenous peroxidase activity was blocked with 3% hydrogen peroxide for 25 min at room temperature in the dark. Nonspecific binding was blocked with 3% BSA for 30 min. Primary antibodies against RRM2B (Proteintech, Cat# 18005-1-AP, 1:100) and VOPP1 (Proteintech, Cat# 12611-1-AP, 1:100) were applied and incubated overnight at 4 °C. After washing with PBS, the sections were incubated with HRP-labeled secondary antibody for 50 min. DAB development was monitored microscopically. Counterstaining was performed with hematoxylin (DAKO, Cat# K5007) for 3 min, followed by differentiation and bluing. Sections were dehydrated through graded alcohol solutions (75% 5 min, 85% 5 min, 100% I 5 min, 100% II 5 min), cleared using xylene, and mounted with neutral balsam (DAKO). The stained sections were scanned with KF-PRO-400 and analyzed with ImageJ software (v1.54p).

### Multiplex immunofluorescence staining

The LUAD formalin-fixed paraffin-embedded sections were first deparaffinized in xylene and then sequentially rehydrated in 100% (twice), 95% (twice), and 70% alcohol, followed by heat-induced antigen retrieval with Tris-EDTA buffer (pH 9.0) and blocking of nonspecific binding. After the blocking buffer was removed, the sections were incubated at 37 °C for 1 h with primary antibodies, including rabbit anti-human CD8 (Abcam, Cat# ab189926, 1:400), rabbit anti-human CD16 (HUABIO, Cat# HA721149, 1:1000), and mouse anti-human PanCK (ZSBIO, Cat# zM-0069, 1:500). The sections were then washed with PBS 3 times and incubated with 100 μL of HRP-labeled secondary antibodies (JACKSON, Cat# 111-035-003, 1:400) for 30 min at 37 °C. Signal amplification was performed using 100 μL of tyramide signal amplification working solution (Runnerbio), and the samples were incubated at room temperature for 10 min. This process was repeated until the sections were stained with all the antibodies of interest. The nuclei were then stained with DAPI. The stained sections were scanned with PANNORAMIC SCAN II and analyzed using ImageJ software (v1.54p).

### Statistical analysis

The analyses included various statistical tests, such as the Wilcoxon test, Fisher’s test, ANNOVAR, and Pearson’s correlation analysis, which were executed in R (v4.1.2). Co-occurrence and mutually exclusive molecular events were identified using the maftools somaticInteractions function, and the significance of pairwise events was determined using Fisher’s test. Principal component analysis was performed with the R package stats (v4.1.2). Heatmaps were generated using the R package ComplexHeatmap (v2.15.4)^98^, while scatter plots, box plots, and histograms were created with the R packages ggplot2 (v3.4.0) and ggpubr (v0.4.0). Venn diagrams were constructed using the R package VennDiagram (v1.7.0).

## Supplementary information

Supplementary Information

## Data Availability

The raw FASTA data derived from whole-genome sequencing (WGS) and RNA-seq have been archived in the National Omics Data Encyclopedia (NODE) under accession number OEP002580 and in the Genome Sequence Archive (GSA) with DNA accession number HRA002624 and RNA accession number HRA002983. The genomic and transcriptomic matrix data, as well as the clinical information used in the analysis, are available through Zenodo (https://zenodo.org/records/15481097).
