# Supplementary material for: Integrative clinico-molecular analysis reveals actionable subtypes and biomarkers in lung adenocarcinoma
Source: Cell Discov. 2026 Jan 28;12:8. doi: 10.1038/s41421-025-00863-4 (PMC12852120; doi:10.1038/s41421-025-00863-4)
Supplement: Supplementary file 1 — Supplementary Information [file 41421_2025_863_MOESM1_ESM.pdf]

Integrative Clinico-Molecular Analysis Reveals Actionable Subtypes and Biomarkers in Lung Adenocarcinoma

Table S1. Clinical phenotype distribution in four subtypes.

		LPI (n = 219)	IMD (n = 219)	IME (n = 222)	HPI (n = 297)	P
Samples		219	219	222	297	
Patients		200	204	209	292	
RNA-seq		219	219	222	297	
WGS		215	215	219	288	
Sex	Female	168	128	129	131	< 0.001
	Male	51	91	93	166	
Age	60-	129	111	82	131	< 0.001
	60+	90	108	140	166	
Smoking	Ever	26	47	63	90	< 0.001
	Never	193	172	159	207	
Stage	AAH /AIS	23	3	7	1	< 0.001
	MIA	75	10	24	1	
	I	115	171	140	133	
	II	3	17	11	39	
	III	3	18	38	116	
	IV	0	0	2	7	
Radiology	Pure GGO	89	16	24	4	< 0.001
	Mixed GGO	96	85	71	15	
	Solid	34	118	127	278	
Histology	AAH/AIS/MIA	98	13	31	2	
	Lepidic	37	21	15	7	
	Papillary	14	44	13	31	
	Acinar	64	128	124	153	
	Micropapillary	0	2	3	13	
	Solid	1	2	22	68	
	Invasive mucinous	3	8	6	11	
	unknown	2	1	8	12	
Survival Status	Alive	208	199	192	211	< 0.001
	Dead	11	20	30	84	
Relapse Status	Yes	211	185	181	181	< 0.001
	No	8	34	41	115	

Table S2. Summary of treatment and follow-up for LPI&IMD and HPI subtypes

	LPI&IMD (Chemotherapy)	LPI&IMD (TKI)	HPI (Chemotherapy)	HPI (TKI)
sample sizes	5	13	10	33
follow-up duration (M)	[31.8 - 59.2]	[12.1 – 120.5]	[3.9 – 95.6]	[8.2 – 120.6]
Overall Status				
Dead	2	2	8	13
Alive	3	11	2	20
Relapse Status				
Yes	4	12	8	33
No	1	1	2	0

Table S3. 20 features and corresponding genes.

Feature	Genes
GGO&L	*KLF15, PLA2G4F, PTGDS, SUSD2, CACNA2D2, CYP4Z2P, ZBTB16, CYP4B1, ACKR1, ZNF385B, PEBP4, DUOX1, LHFPL3-AS2, HSD17B6, MS4A15, WIF1, GGTLC1, AQP4, SFTPC, SFTPB, PGC, SFTPD*
Solid&M	*BIRC5, UBE2C, TPX2, MYBL2, MKI67, CENPF, TOP2A, ANLN, PLOD2, POSTN, S100A9, SULF1, CYP24A1, MMP12, COL11A1, NME1-NME2, DSP, MMP9, MUC16, MMP1, IGHGP*
Metastasis	*TUBB3, VCAN, EGLN3, FAM83A, PLAU, SLC2A1, AHNAK2, MMP11, GREM1, SPP1, PLXNB3, MIR4260, FNDC1, THBS2, COL5A2, CTHRC1, COL1A1, B3GNT6, GJB2, LRRC15, MIR675, COL3A1, IGF2, XAGE1B, TFF3, CRABP2, CST1, CA9, TFPI2, MT2P1, SERPINE1*
Matrix remodeling	*CA9, MMP9, MMP2, MMP1, MMP3, MMP12, MMP7, MMP11, PLOD2, ADAMTS4, ADAMTS5, LOX*
Tumor proliferation	*MKI67, ESCO2, CETN3, CDK2, CCND1, CCNE1, AURKA, AURKB, E2F1, MYBL2, BUB1, PLK1, CCNB1, MCM2, MCM6*
Angiogenesis	*VEGFA, VEGFB, VEGFC, PDGFC, CXCL8, CXCR2, FLT1, PGF, CXCL5, KDR, ANGPT1, ANGPT2, TEK, VWF, CDH5*
EMT	*VIM, CDH1, CDH2, FN12, ZEB1, ZEB2, MMP2, SNAI1, SNAI2, SPARC, TWIST1, CCN2, EPCAM, CDH11, CLDN4, ESRP1, TGFB1*
Endothelium	*CDH5, CLEC14A, ECSCR, EMCN, ESAM, FLT1, KDR, MMRN2, PTPRB, ROBO4, TEK, TIE1, VWF, ACVRL1, MMRN1, MYCT1, CLDN5, SOX18*
CAF	*ACTA2, CD248, COL1A1, COL1A2, COL5A1, COL6A1, COL6A2, COL6A3, FAP, FBLN1, LUM, PDGFRB, COL3A1, TAGLN, DPT, ISLR, COL14A*
CD8T	*CD8A, CD8B, EOMES, GZMA, GZMB, GZMK, PRF1, TBX21, CD3D, CD3G, CD7, CRTAM, GZMH, THEMIS*
CD4T	*ANKRD55, CCR4, CD2, CD28, CD3D, CD3E, CD40LG, CD5, CD6, ITK, LEF1, TRAT1, UBASH3A, BCL11B, IL7R, RCAN3*
B cell	*CD79A, CD79B, MS4A1, BLK, CD19, CD22, CR2, FCRL5, PAX5, STAP1, TNFRSF13B, TNFRSF13C, BANK1, CD37, CXCR5, FCRL2, HLA-DOB, IGKC, POU2AF1, SPIB, VPREB3, ADAM28, CD72, GPR18, LY86, P2RX5, PNOC, RALGPS2, SP140, TCL1A*
Macrophage	*CSF1R, C1QA, C1QC, CCR1, CD14, CD163, CLEC5A, CMKLR1, CYBB, MS4A4A, MSR1, SIGLEC1, TREM2, FPR3, LILRB4, VSIG4, HAMP, MS4A6A*
Treg	*FOXP3, CTLA4, IKZF2, IKZF4, CCR4, IL2RA, LTB, CD5, LAIR2, ICOS*
NK	*NKG7, CD160, EOMES, GNLY, GZMB, GZMH, IFNG, KLRF1, NCR1, NCR3, CCL4, CD247, CTSW, FASLG, IL2RB, KLRD1, PLEKHF1, PRF1, S1PR5, TBX21*
Neutrophil	*CXCR1, CXCR2, FCGR3B, PGLYRP1, FPR2, MMP25, VNN3, CEACAM3*
Myeloid	*IL10, CYBB, IDO1, ARG1, PTGS2, IL4I1, IL6*
DC	*ALOX15, CCL17, CCL22, CD1A, CD1B, CD1C, CD1E, CD80, CD86, ETV3, FCER2, FPR3, HLA-DQA1, IL12B, MAP3K13, SLAMF8, SLCO5A1, TREM2*
M1	*CMKLR1, IL12B, SOCS3, TNF, C3AR1, CSF1R, CYBB, IL10, LILRB4, MMP19, SIGLEC1, TNFAIP6, TNIP3, ADAMDEC1, CCL19, CCL8, CXCL9, PTGIR*
Th	*CD40LG, IFNG, IL21, CD2, LCK, IL5, IL13*

Table S4. NCCN hotsopts.

Type	Events
EGFR	Exon 18 p.G719, Exon 19 del, Exon 20 Insertion, Exon 20 p.S768I, Exon 21 p.L858R, p.L861Q
KRAS	p.G12C
BRAF	p.V600E
ERBB2	Exon 20
Fusion	ALK, ROS1, MET, RET

Table S5. Primer and oligonucleotide sequences.

**PCR primers**		
**Primer**	**Primer Sequence**	**Note**
RRM2B-F	AGAGGCTCGCTGTTTCTATGG	qPCR
RRM2B-R	GCAAGGCCCAATCTGCTTTTT	qPCR
LRRC14-F	ATGCACACGCTTGTGTTCTTG	qPCR
LRRC14-R	GTGTACCAACTCGCGCAGTA	qPCR
VPS28-F	CCTGGTCCAATACAAAGCTGC	qPCR
VPS28-R	TCCATGACCGTGATGAAGAGC	qPCR
TSNARE-F	CCCCTAGAGTGCGCTAGATGT	qPCR
TSNARE-R	GCCCTTGGGACAATAGGCG	qPCR
CYP4B1-F	CCCACTCTGGTTCGGACAG	qPCR
CYP4B1-R	ATACACATCAGGGGCCTTAGG	qPCR
**siRNA oligos**		
siRRM2B-1	CTGCTATATTCTGGCTAAATT	siRNA
siRRM2B-2	CTTGCCTGATGTTCCAATATT	siRNA
siVOPP1-1	UGGUGUAGGACACAUUGAAGG	siRNA
siVOPP1-2	AAGGAACCAGAAGUACCACAG	siRNA

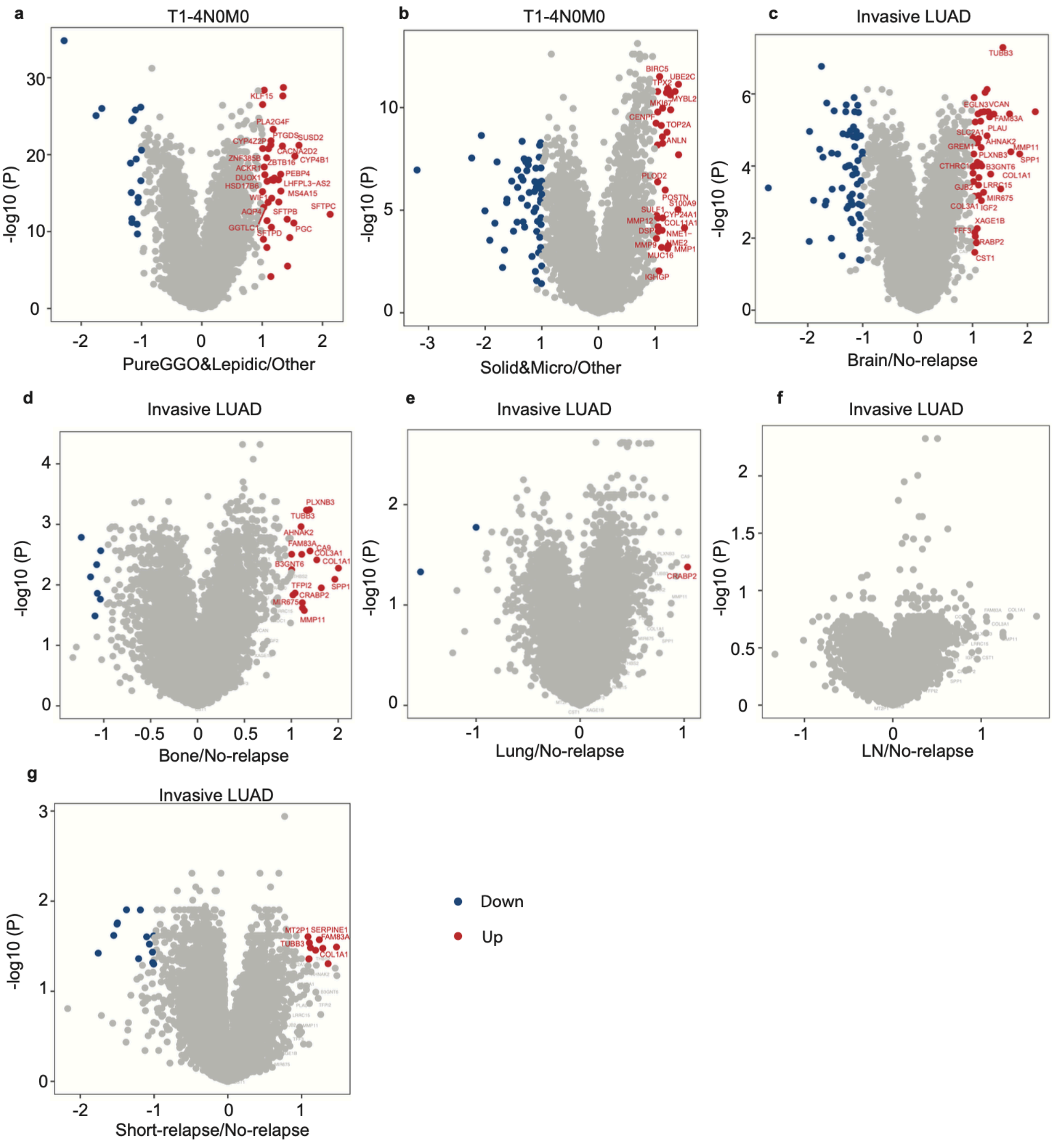

Fig. S1. Radio-pathological specific gene expression marker filter. (**a**) Volcano plot illustrating genes with specific high expression in T1N0M0 lung adenocarcinoma samples of pure ground-glass opacity (GGO) and lepidic subtype. (**b**) Volcano plot illustrating genes with specific high expression in T1N0M0 lung adenocarcinoma samples of solid and micropapillary subtype. Volcano plot depicting genes with specific high expression in invasive lung adenocarcinoma samples associated with various relapse sites and short relapse, including brain (**c**), bone (**d**), lung (**e**), lymph nodes (LN) (**f**), and short-relapse (**g**).

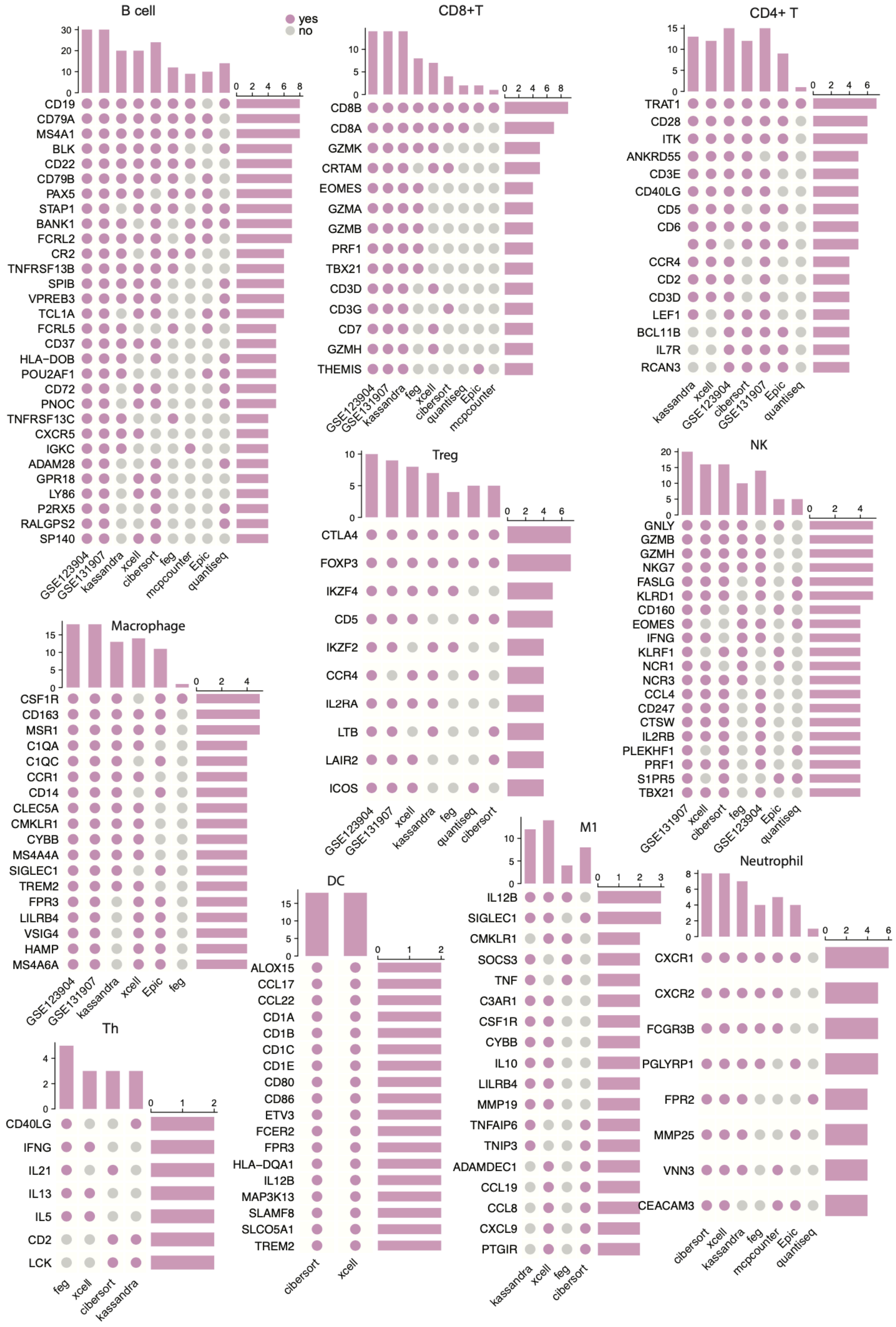

Fig. S2. Filter for tumor microenvironment-specific gene expression markers. Details of each immune cell marker gene obtained from single-cell datasets and published literature typically include information about genes that are specifically associated with different immune cell types.

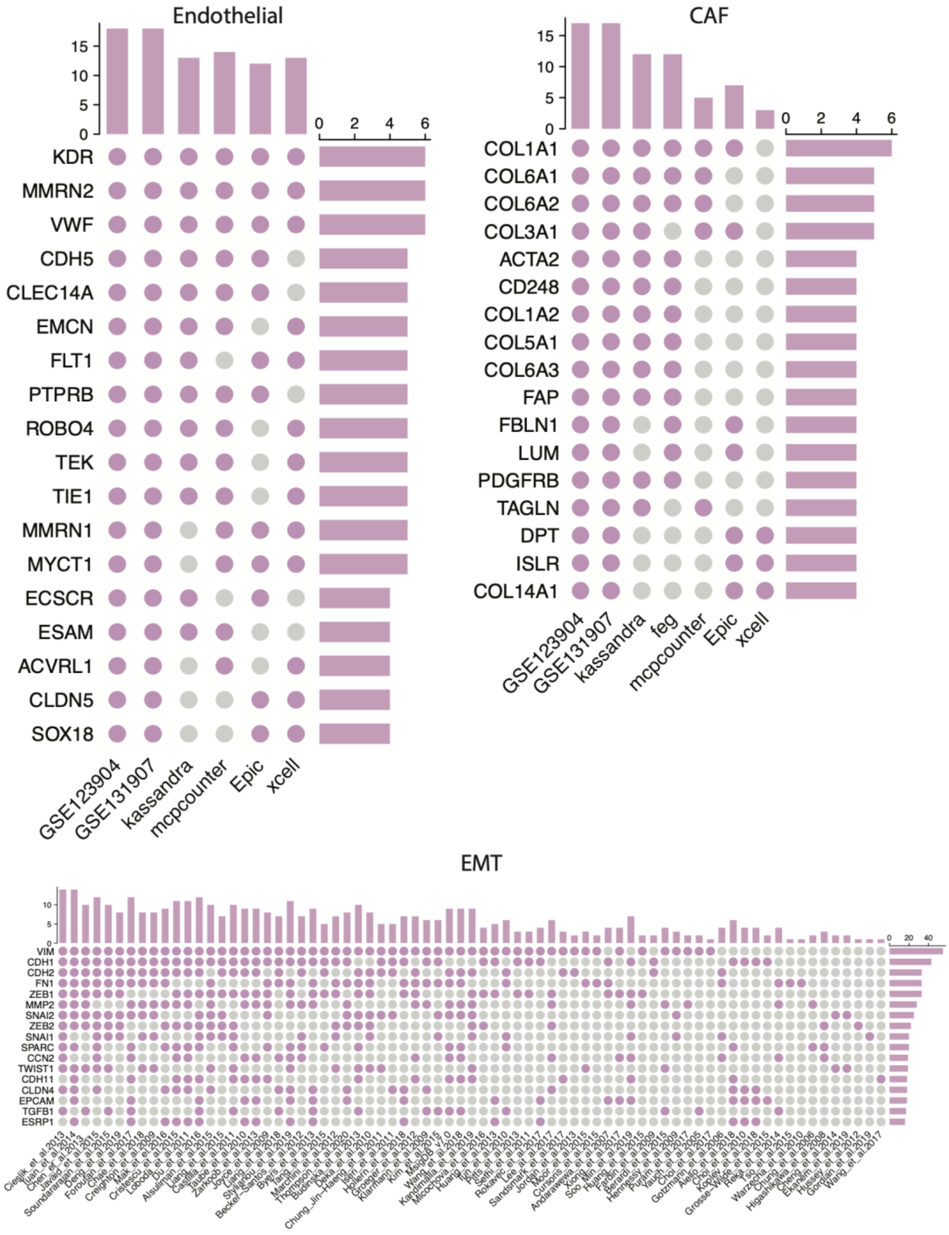

Fig. S3. Filter for tumor microenvironment-specific gene expression markers. The distribution of marker genes for endothelial cells, Cancer-Associated Fibroblasts (CAF), and Epithelial-Mesenchymal Transition (EMT) from single-cell datasets and published literature.

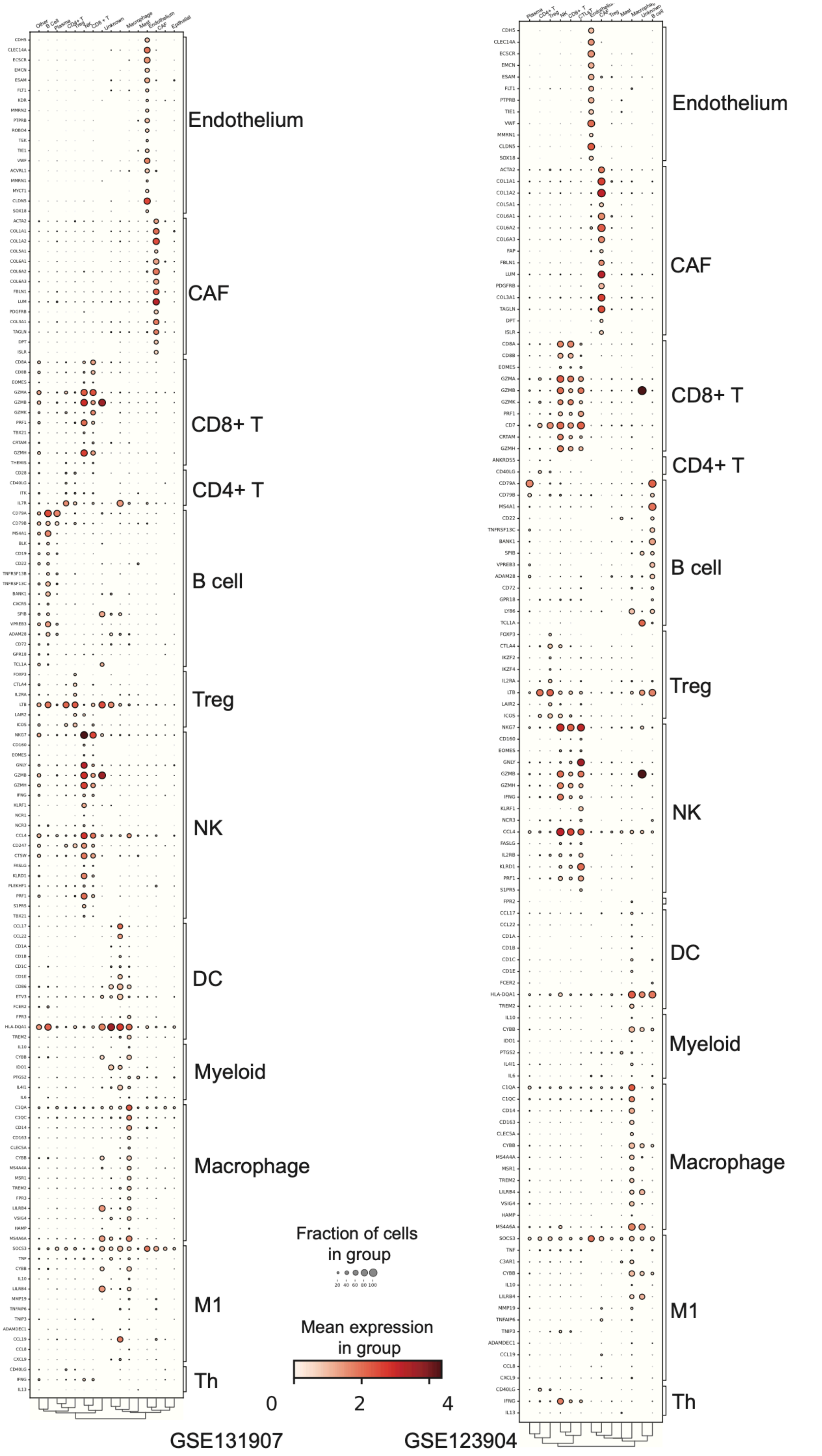
Endothelium
CAF
CD8+ T
CD4+ T
B cell
Treg
NK
DC
Myeloid
Macrophage
M1
Th
Fraction of cells in group
20 40 60 80 100
Mean expression in group
0
2
4
GSE131907
GSE123904

Fig. S4. Overview of cell marker genes in single cell datasets. A dot plot illustrating the mean expression of marker genes for 12 major cell types in Lung Adenocarcinoma (LUAD) provides a visual representation of the gene expression levels across different cell types

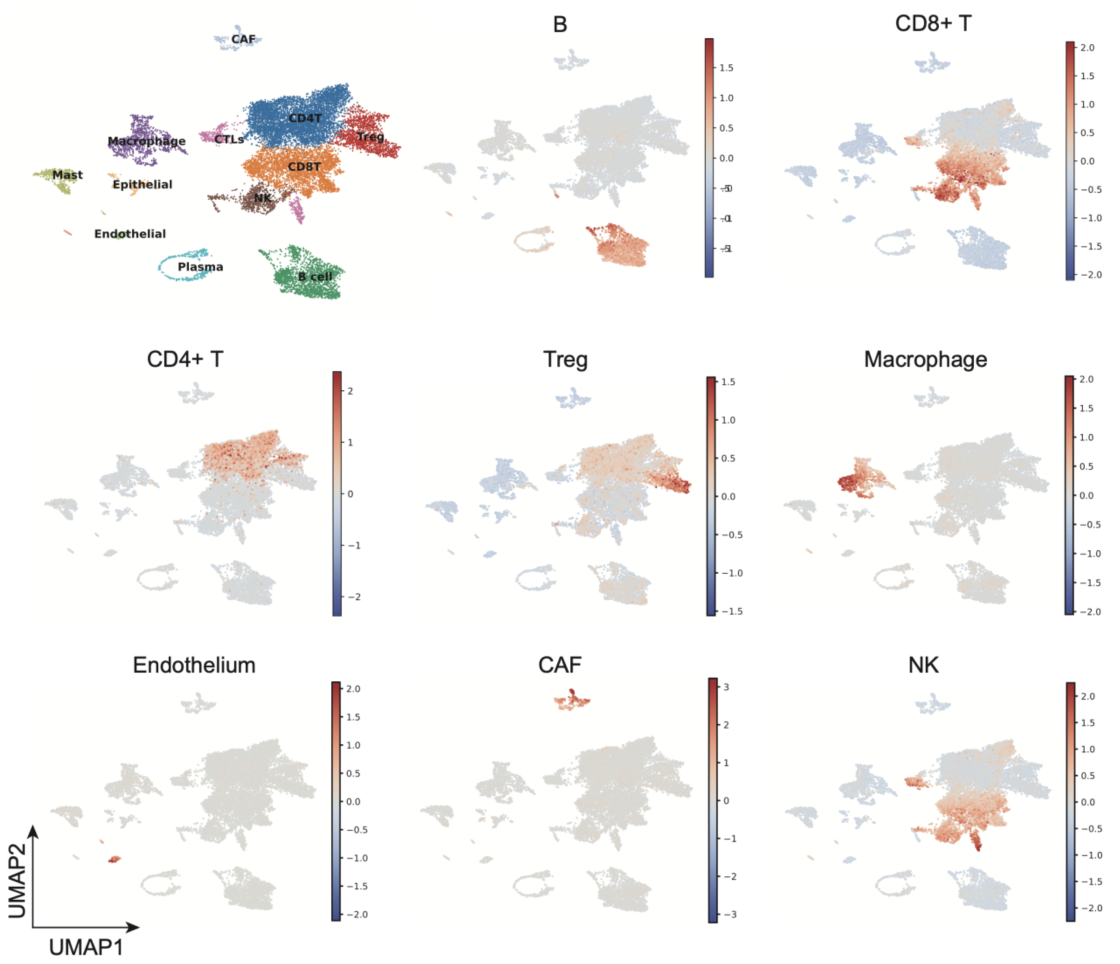

Fig. S5. UMAP projections of 21,795 Lung Adenocarcinoma (LUAD) cells from the GSE123904 dataset were visualized, and the cells were colored based on scores calculated from the expression of cell marker genes for B cells, CD8+ T cells, CD4+ T cells, T regulatory (Treg) cells, Macrophages, Endothelial cells, Cancer-Associated Fibroblasts (CAF), and Natural Killer (NK) cells.

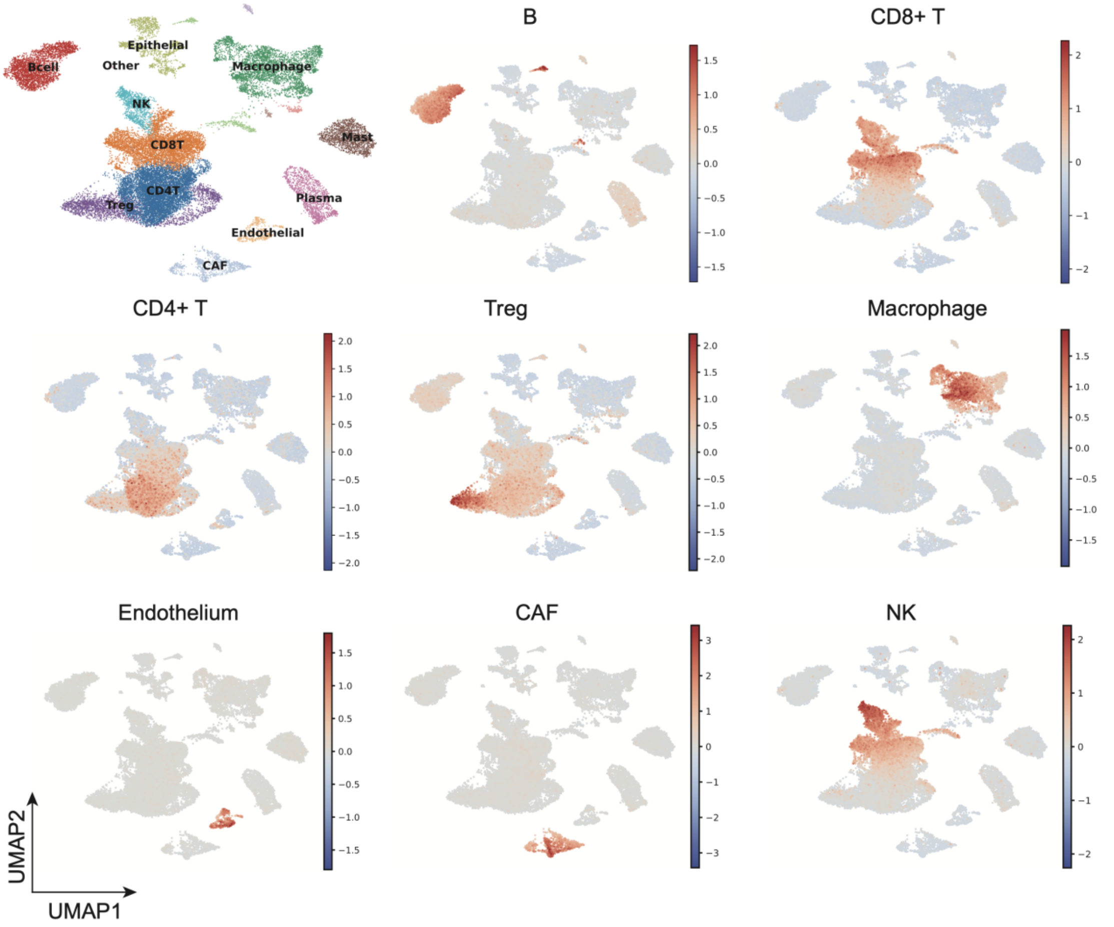

Fig. S6. UMAP projections of 29,749 Lung Adenocarcinoma (LUAD) cells from the GSE131907 dataset were visualized, and the cells were colored based on scores calculated from the expression of cell marker genes for B cells, CD8+ T cells, CD4+ T cells, T regulatory (Treg) cells, Macrophages, Endothelial cells, Cancer-Associated Fibroblasts (CAF), and Natural Killer (NK) cells.

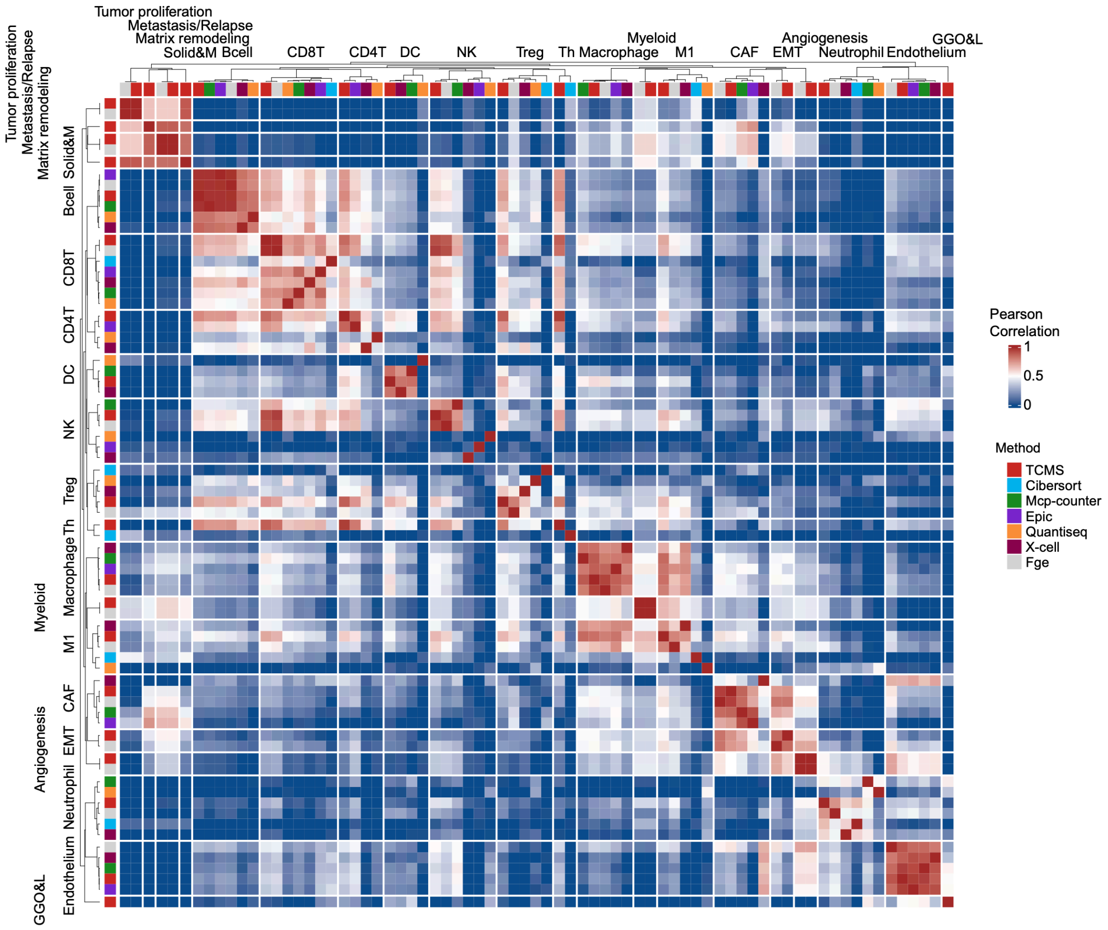

Fig. S7. Pearson correlation coefficients were computed to assess the association between TCMS and cell scores derived from six published methods across 957 Lung Adenocarcinoma (LUAD) samples from our cohort.

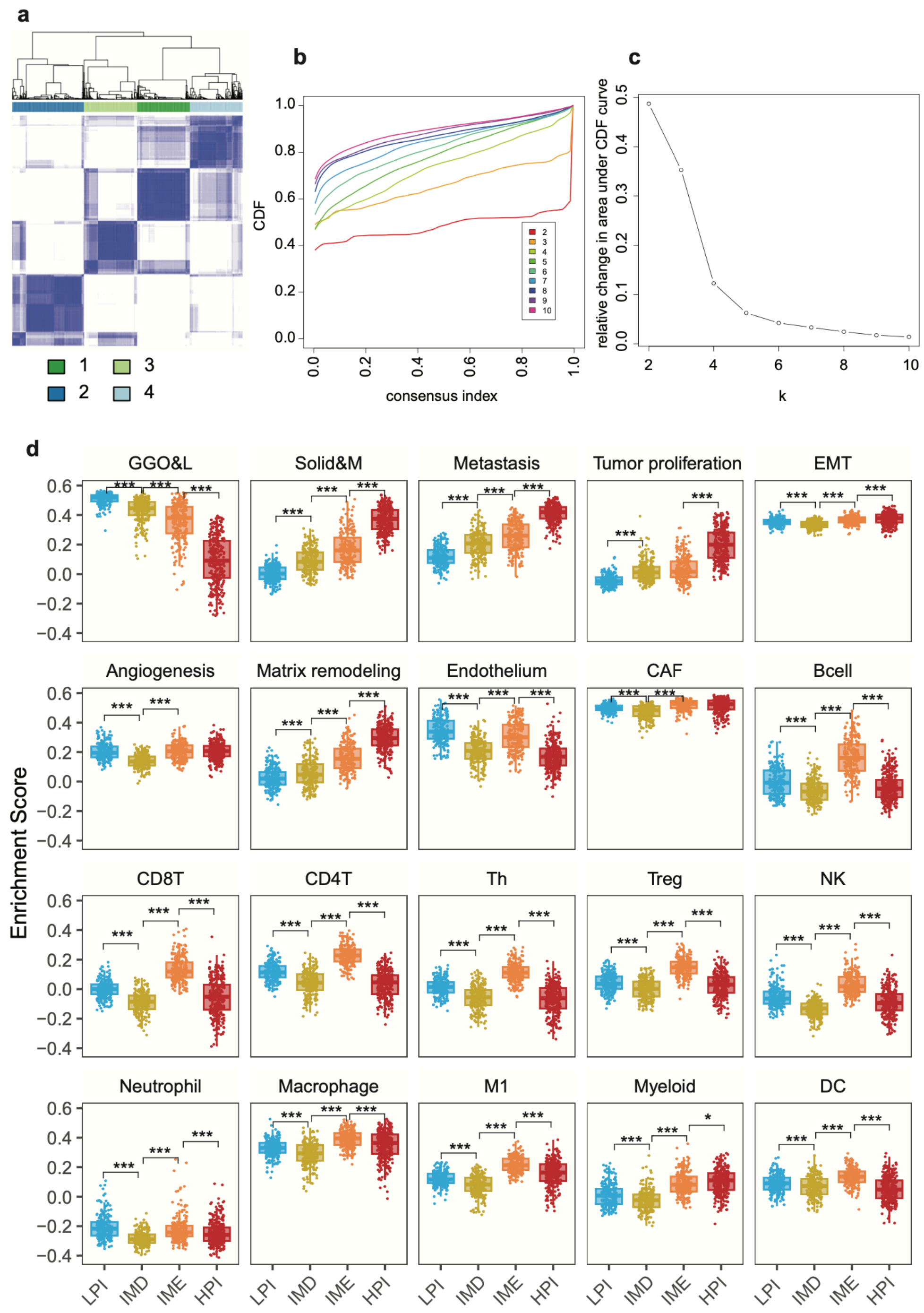

Fig. S8. Identification of four subtypes and the corresponding distribution of 20 radio-pathology and tumor microenvironment (TME) gene signature scores. (**a**) The consensus matrix for unsupervised clustering at 4 clusters provides insight into the level of agreement within each cluster and the distinctions between clusters. The consensus cumulative distribution function (CDF) (**b**) and the relative change in the area under the CDF curve (**c**) during the unsupervised clustering process. (**d**) Distribution comparison of 20 radio-pathology and tumor microenvironment (TME) gene signature scores among the four subtypes. Statistical analyses were performed for pairwise comparisons between LPI vs IMD, IMD vs IME, and IME vs HPI. * FDR < 0.05, *** FDR < 0.001.

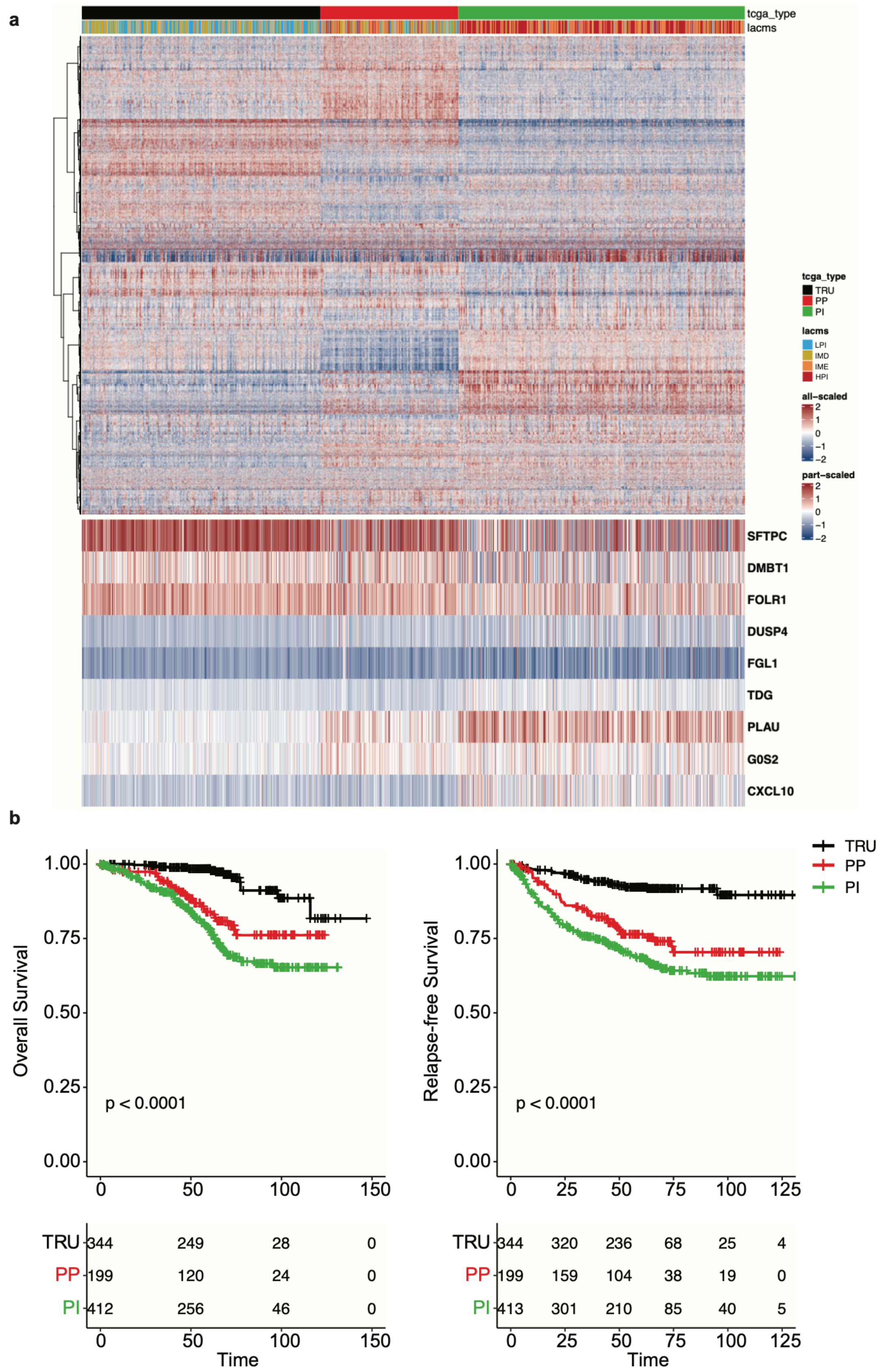

Fig. S9. Constructed three subtypes of TCGA based on gene expression using RNA-seq data from our cohort comprising 957 LUAD samples (**a**) and identified significant differences in survival outcomes (**b**).

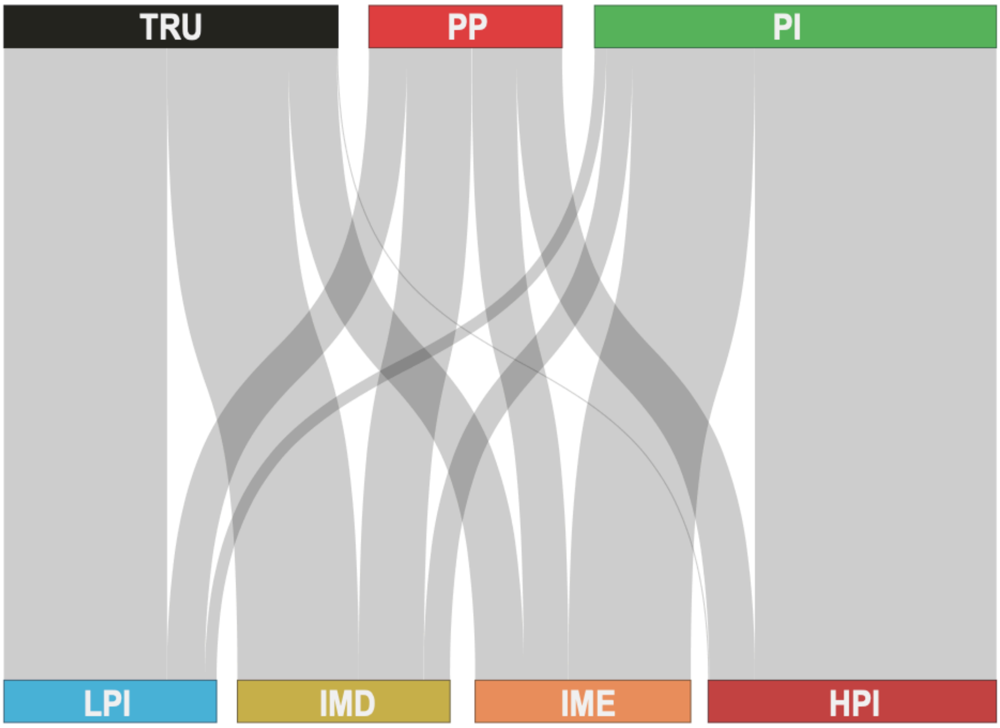

Fig. S10. The Sankey plot visualizes the flow of TCGA TRA, PP, and PI subtypes into LPI, IMD, IME, and HPI subtypes across 957 samples.

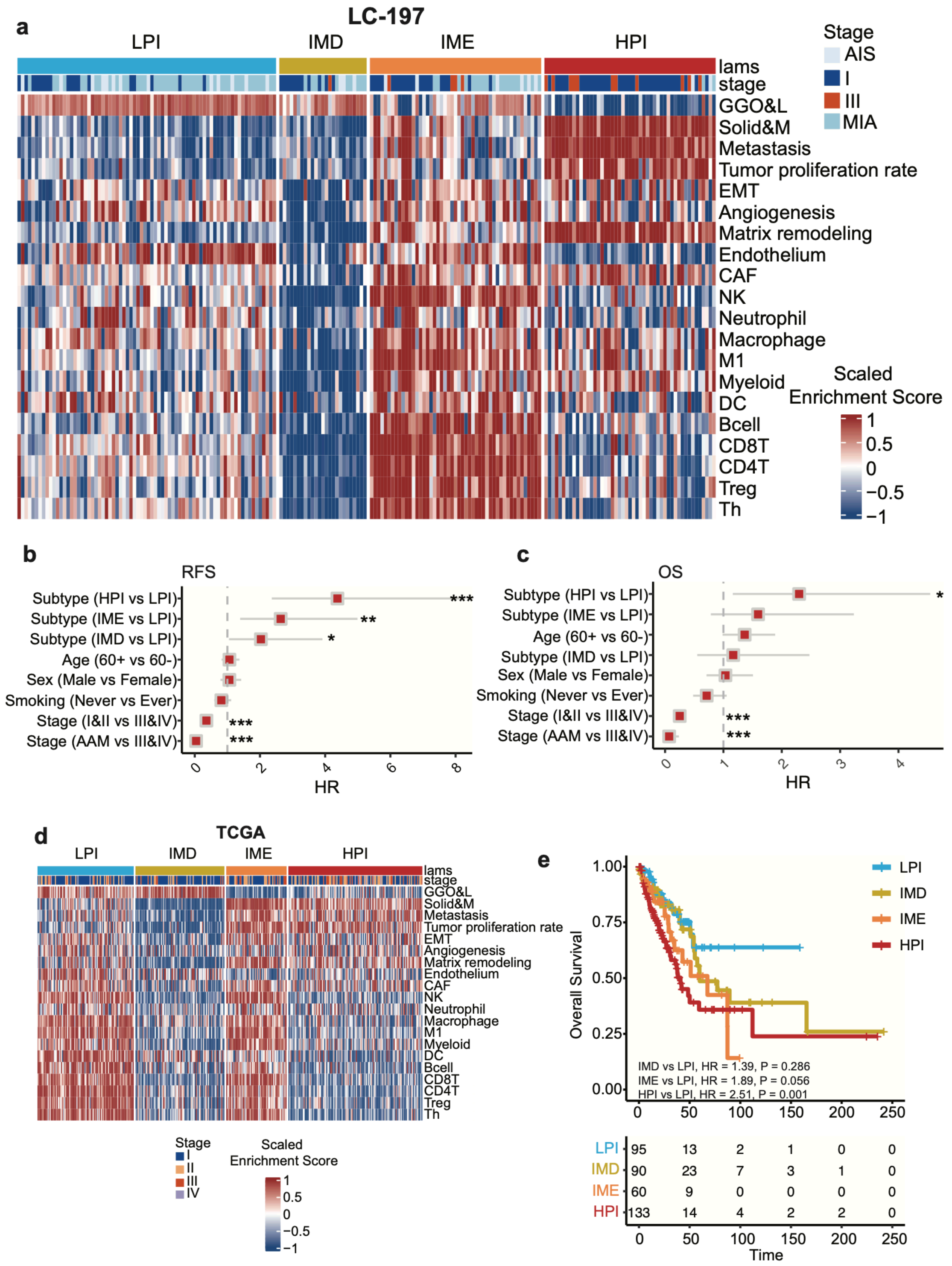

Fig. S11. Identification of four distinct subtypes in the published LUAD cohort based on radio-pathology and tumor microenvironment (TME) gene expression signatures. (**a**) Heatmap displaying the scores of 20 radio-pathology TME gene expression signatures among the four subtypes in LC-197. The multivariate Cox analysis of four subtypes in RFS (**b**) and OS (**c**). Heatmap displaying the scores of 20 radio-pathology TME gene expression signatures among the four subtypes in TCGA (**d**) cohorts. (**e**) Overall survival distribution of the four subtypes in the TCGA cohort.

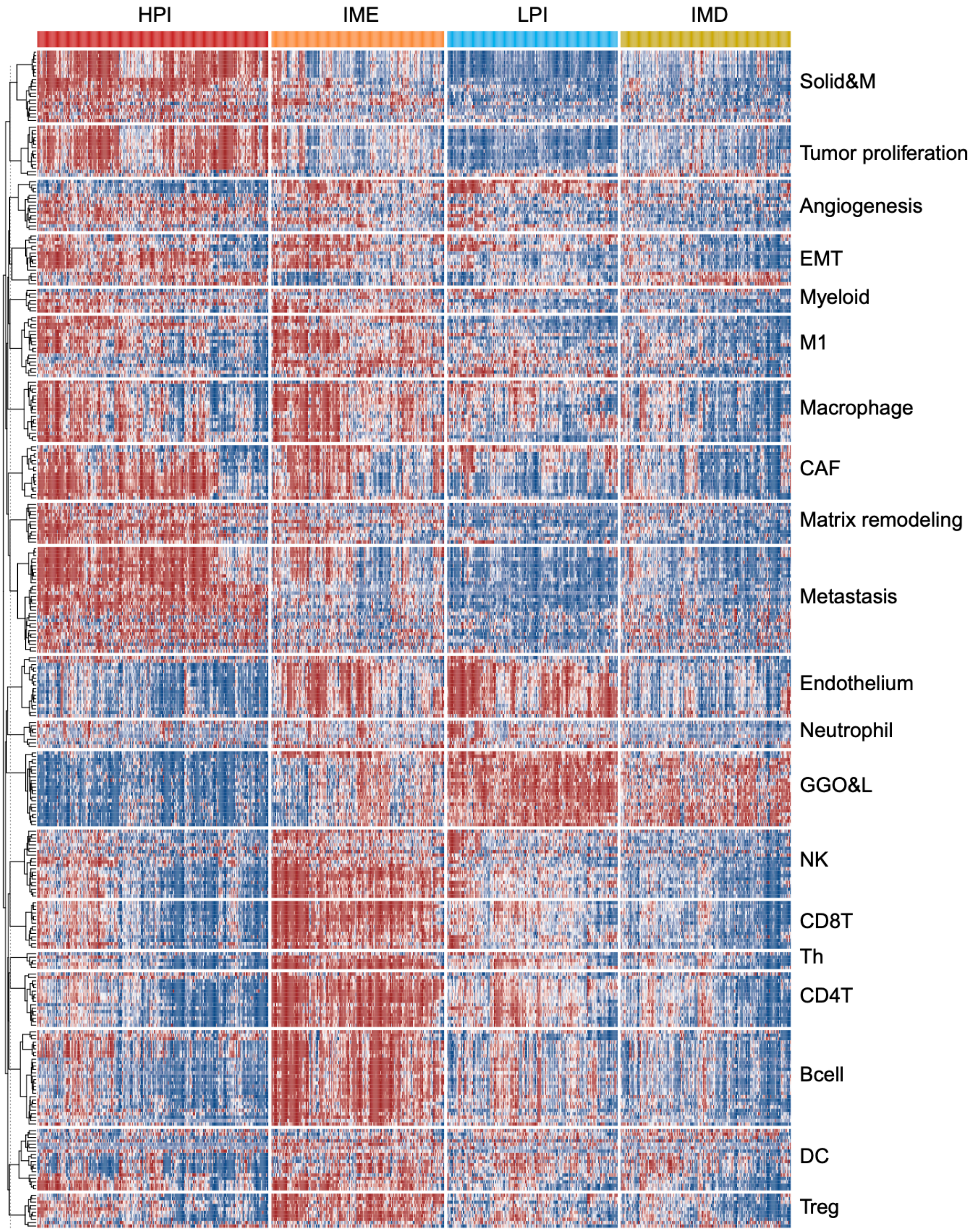

Fig. S12. The heatmap displaying the expression distribution of genes associated with 20 features across four subtypes.

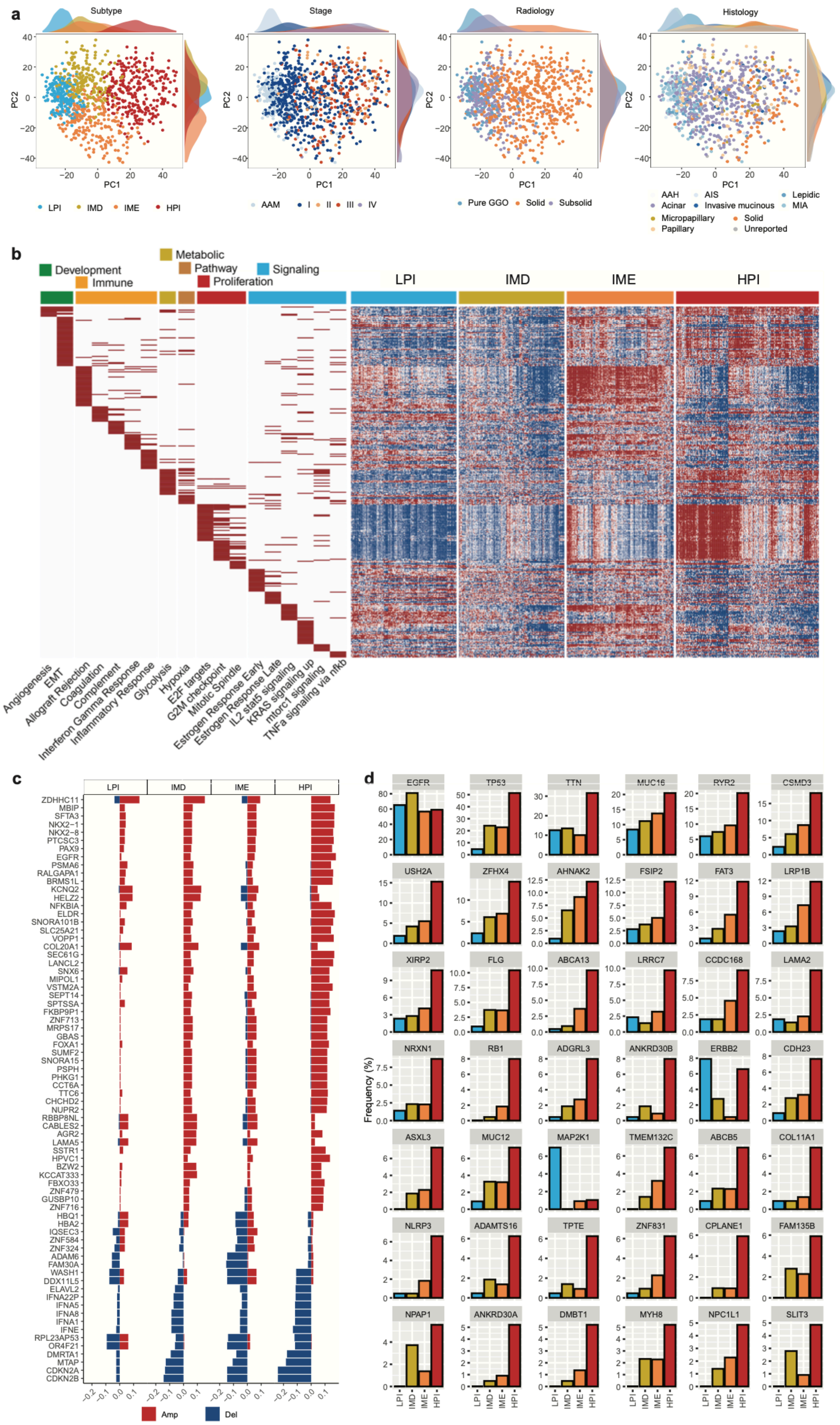
a
Subtype
Stage
Radiology
Histology
PC1
PC2
LPI
IMD
IME
HPI
AAM
I
II
III
IV
Pure GGO
Solid
Subsolid
AAH
AIS
Lepidic
Acinar
Invasive mucinous
MIA
Micropapillary
Solid
Papillary
Unreported
b
Development
Immune
Metabolic
Pathway
Proliferation
Signaling
LPI
IMD
IME
HPI
Angiogenesis
EMT
Allograft Rejection
Coagulation
Complement
Interferon Gamma Response
Inflammatory Response
Glycolysis
Hypoxia
E2F targets
G2M checkpoint
Mitotic Spindle
Estrogen Response Early
Estrogen Response Late
IL2 stat5 signaling
KRAS signaling up
mtorc1 signaling
TNFa signaling via nfkb
c
LPI
IMD
IME
HPI
ZDHHC11
MBIP
SFTA3
NKX2−1
NKX2−8
PTCSC3
PAX9
EGFR
PSMA6
RALGAPA1
BRMS1L
KCNQ2
HELZ2
NFKBIA
ELDR
SNORA101B
SLC25A21
VOPP1
COL20A1
SEC61G
LANCL2
SNX6
MIPOL1
VSTM2A
SEPT14
SPTSSA
FKBP9P1
ZNF713
MRPS17
GBAS
FOXA1
SUMF2
SNORA15
PSPH
PHKG1
CCT6A
TTC6
CHCHD2
NUPR2
RBBP8NL
CABLES2
AGR2
LAMA5
SSTR1
HPVC1
BZW2
KCCAT333
FBXO33
ZNF479
GUSBP10
ZNF716
HBQ1
HBA2
IQSEC3
ZNF584
ZNF324
ADAM6
FAM30A
WASH1
DDX11L5
ELAVL2
IFNA22P
IFNA5
IFNA8
IFNA1
IFNE
RPL23AP53
OR4F21
DMRTA1
MTAP
CDKN2A
CDKN2B
Amp
Del
d
Frequency (%)
EGFR
TP53
TTN
MUC16
RYR2
CSMD3
USH2A
ZFHX4
AHNAK2
FSIP2
FAT3
LRP1B
XIRP2
FLG
ABCA13
LRRC7
CCDC168
LAMA2
NRXN1
RB1
ADGRL3
ANKRD30B
ERBB2
CDH23
ASXL3
MUC12
MAP2K1
TMEM132C
ABCB5
COL11A1
NLRP3
ADAMTS16
TPTE
ZNF831
CPLANE1
FAM135B
NPAP1
ANKRD30A
DMBT1
MYH8
NPC1L1
SLIT3
LPI
IMD
IME
HPI

Fig. S13. Comparisons of genomic and transcriptomic events among the four subtypes. (**a**) PCA projections of 957 samples with RNA-seq data, color-coded based on the four subtypes, Pathology stage, Radiology, and Histology classification. (**b**) Heatmap displaying the expression of Differentially Expressed Genes (DEGs) in comparisons (IMD vs LPI, IME vs IMD, HPI vs IME, and HPI vs LPI) along with associated hallmarks of cancer. (**c**) Frequency distribution of significantly differentially expressed copy number genes across the four subtypes. (**d**) Frequency distribution of significantly differentially mutated genes across the four subtypes.

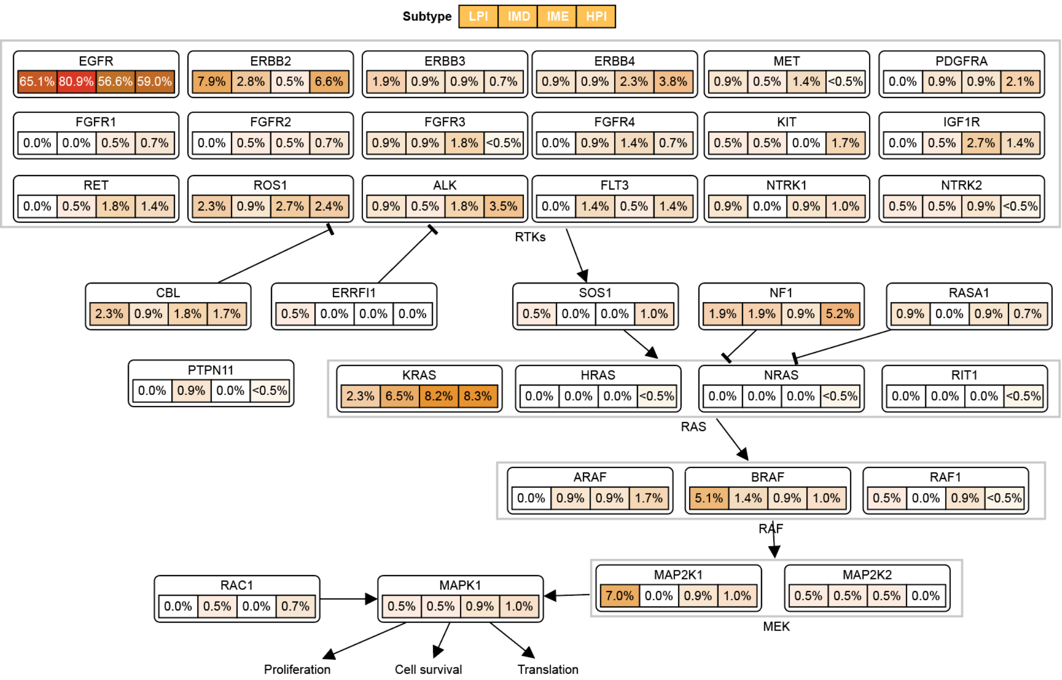

Fig. S14. The mutation frequency of genes from the RTK-RAS pathway was assessed among the four subtypes.

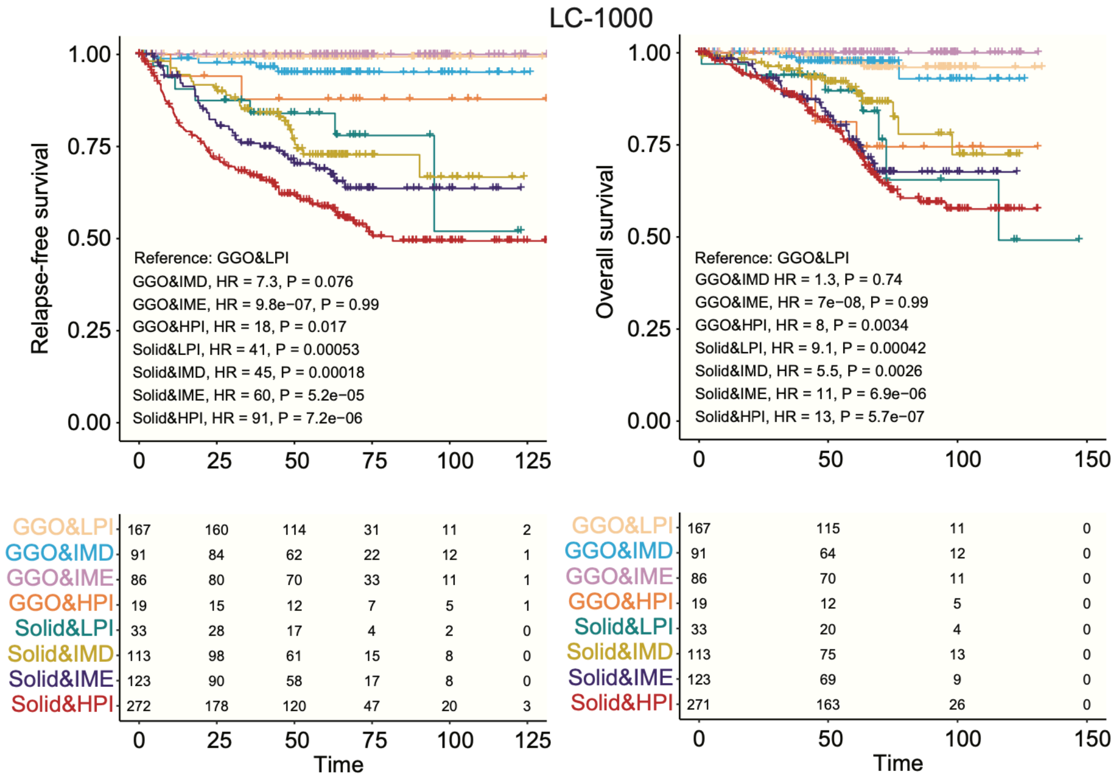

Fig. S15. The comparison of OS and RFS among nine groups classified based on radiology and the four subtypes in LC-1000 cohort

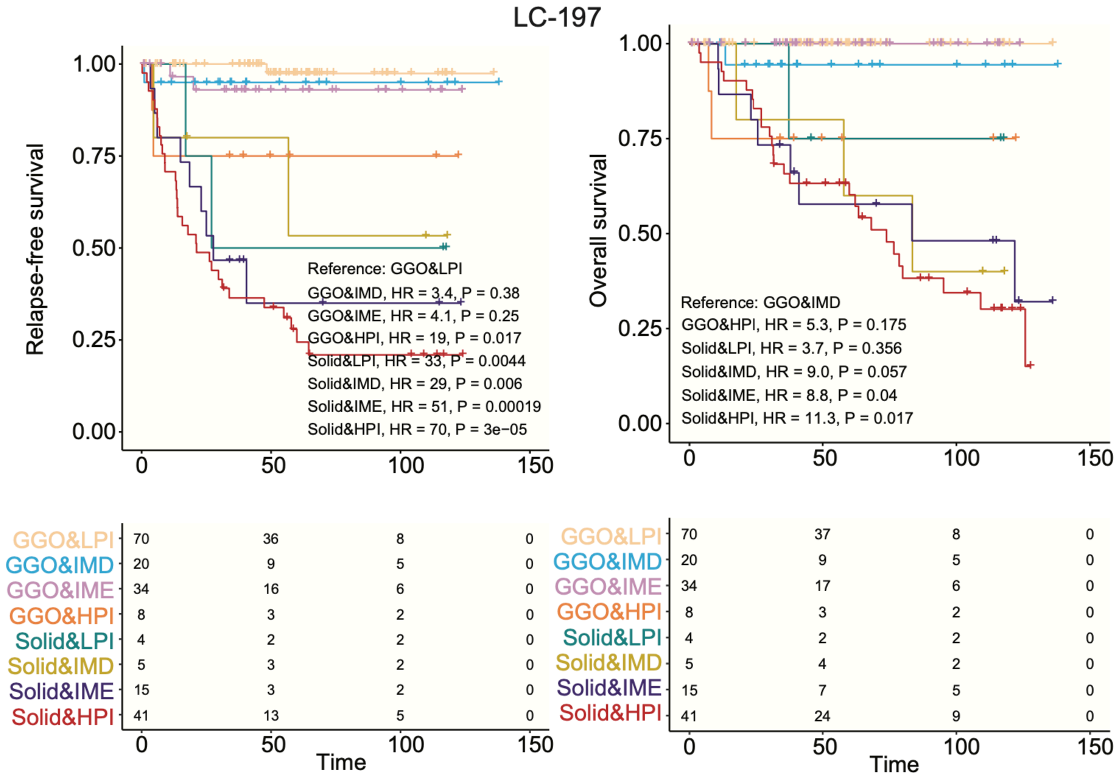

Fig. S16. The comparison of OS and RFS among nine groups classified based on radiology and the four subtypes in LC-197 cohort

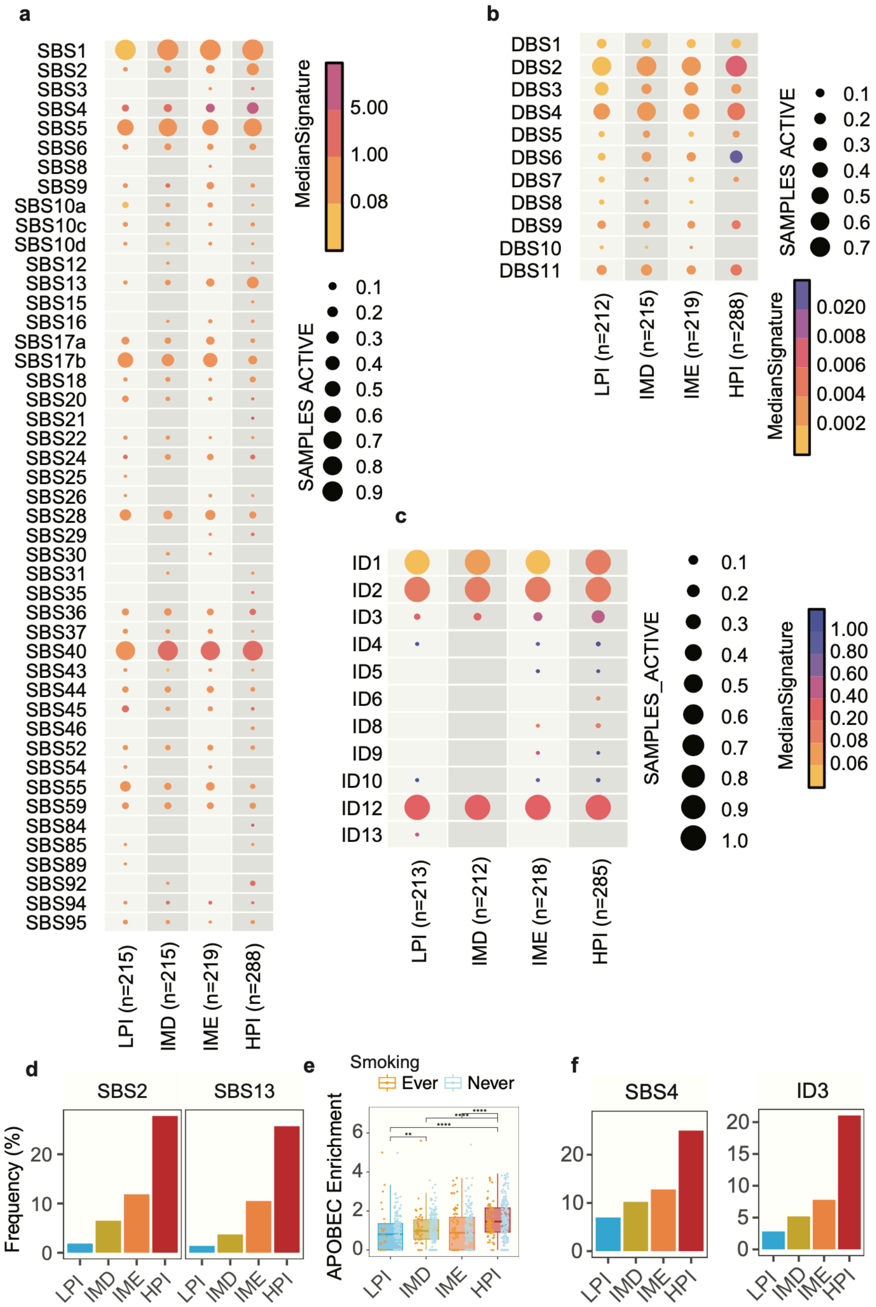

Fig. S17. The distribution of Single Base Substitution (SBS), Double Base Substitution (DBS), and Insertion-Deletion (ID) mutation signatures within the four subtypes. The count of mutations associated with distinct mutational signatures, namely SBS (**a**), DBS (**b**), and ID (**c**), in LUAD across four subtypes. The size of the circle indicates the percentage of tumors expressing signature activity within each subtype, while the color reflects the median number of mutations per megabase associated with a specific signature in the respective subtype. (**d**) Bar plot depicting the frequency of SBS2 and SBS13 (APOBEC-related signature) within the four subtypes. (**e**) Box plot illustrating the APOBEC Enrichment score distribution among the four subtypes. (**f**) Bar plot depicting the frequency of SBS4 and ID3 (Smoking-related signature) within the four subtypes.

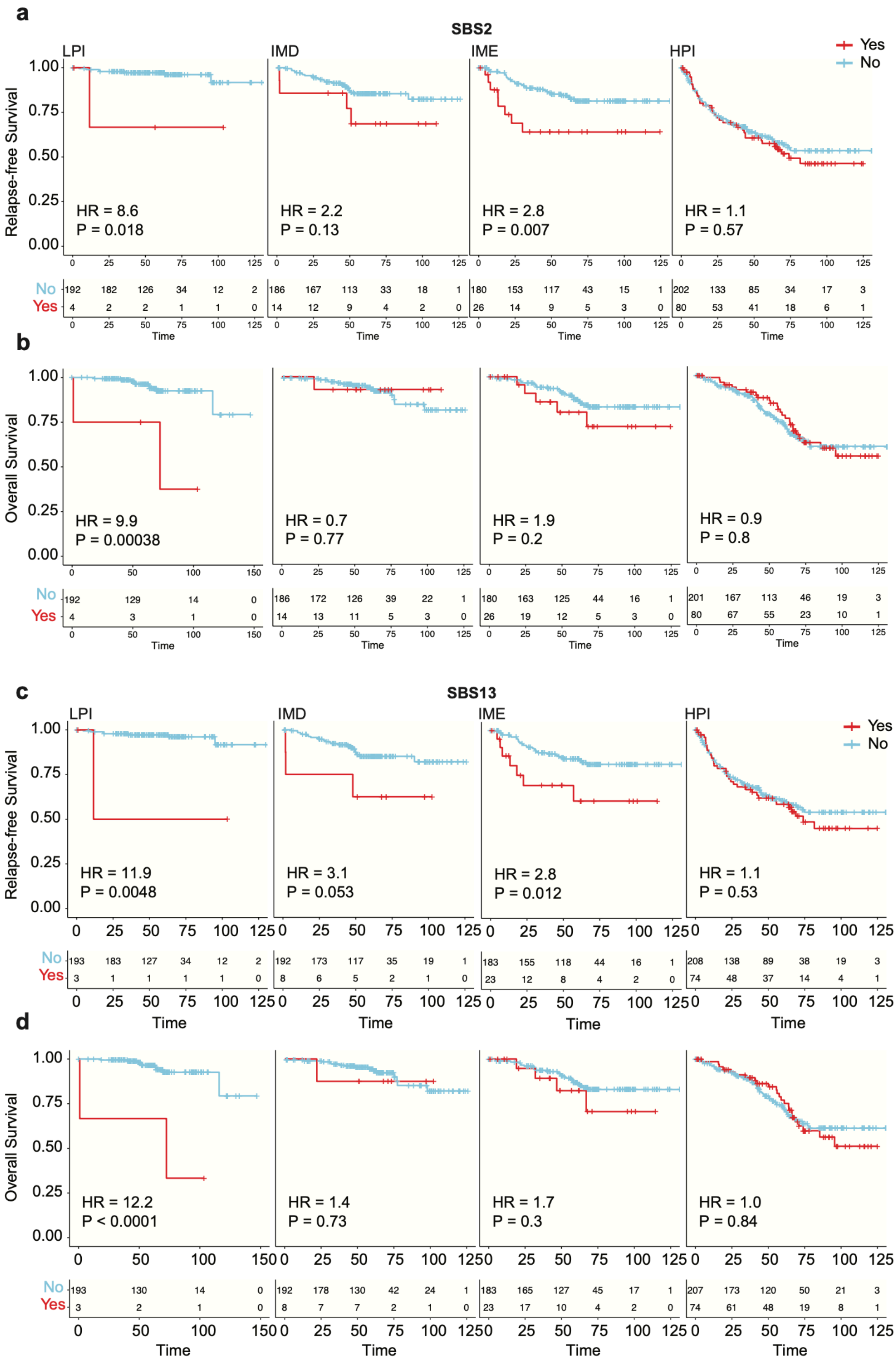

Fig. S18. Survival analysis of APOBEC signature within the four subtypes. Distribution of OS and RFS between samples with activated SBS2 (**a** and **b**) or SBS13 (**c** and **d**) signatures and those without activation.

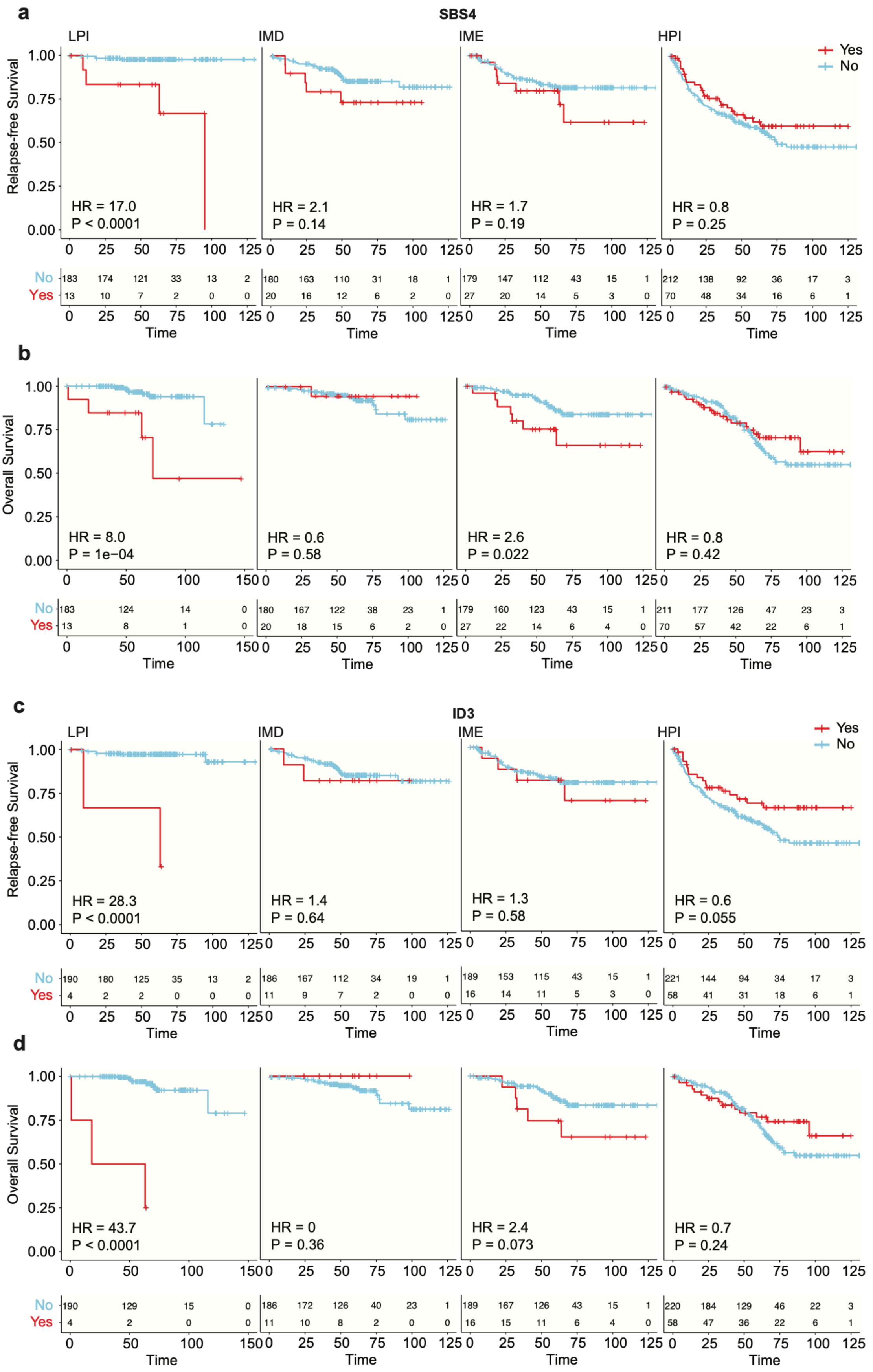

Fig. S19. Survival analysis of smoking signature within the four subtypes. Distribution of OS and RFS between samples with activated SBS4 (**a** and **b**) or

ID3 (**c** and **d**) signatures and those without activation.

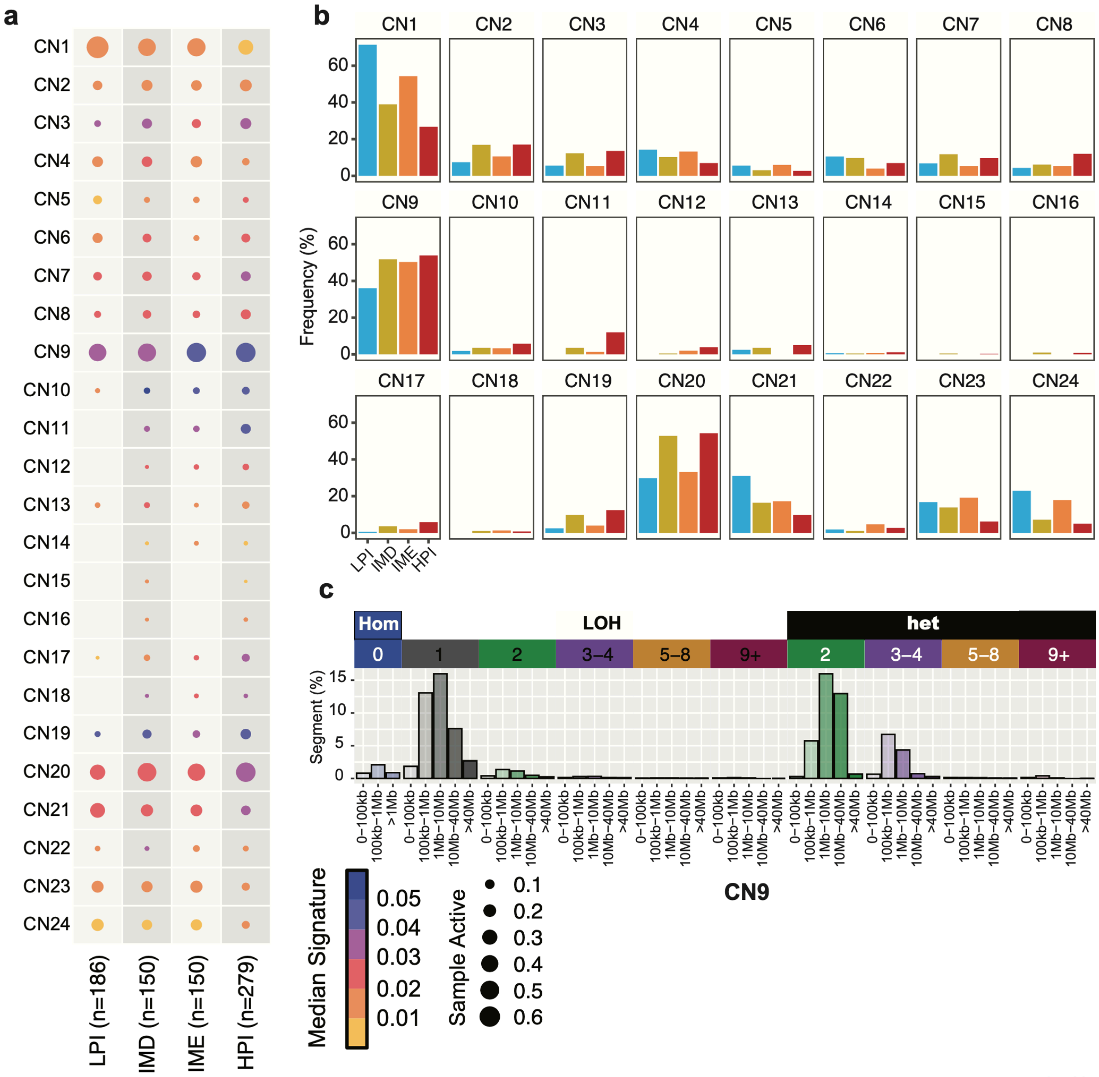

Fig. S20. The distribution of CNV signatures within the four subtypes. (**a**) The count of mutations associated with mutational signatures across four subtypes. The size of the circle indicates the percentage of tumors expressing signature activity within each subtype, while the color reflects the median number of mutations per megabase associated with a specific signature in the respective subtype. (**b**) Bar plot depicting the frequency of CNV signatures within the four subtypes. (**c**) Decomposition plot illustrates the heterozygosity (Het) status, total copy number (0–9+), and segment sizes associated with the CN9 signature.

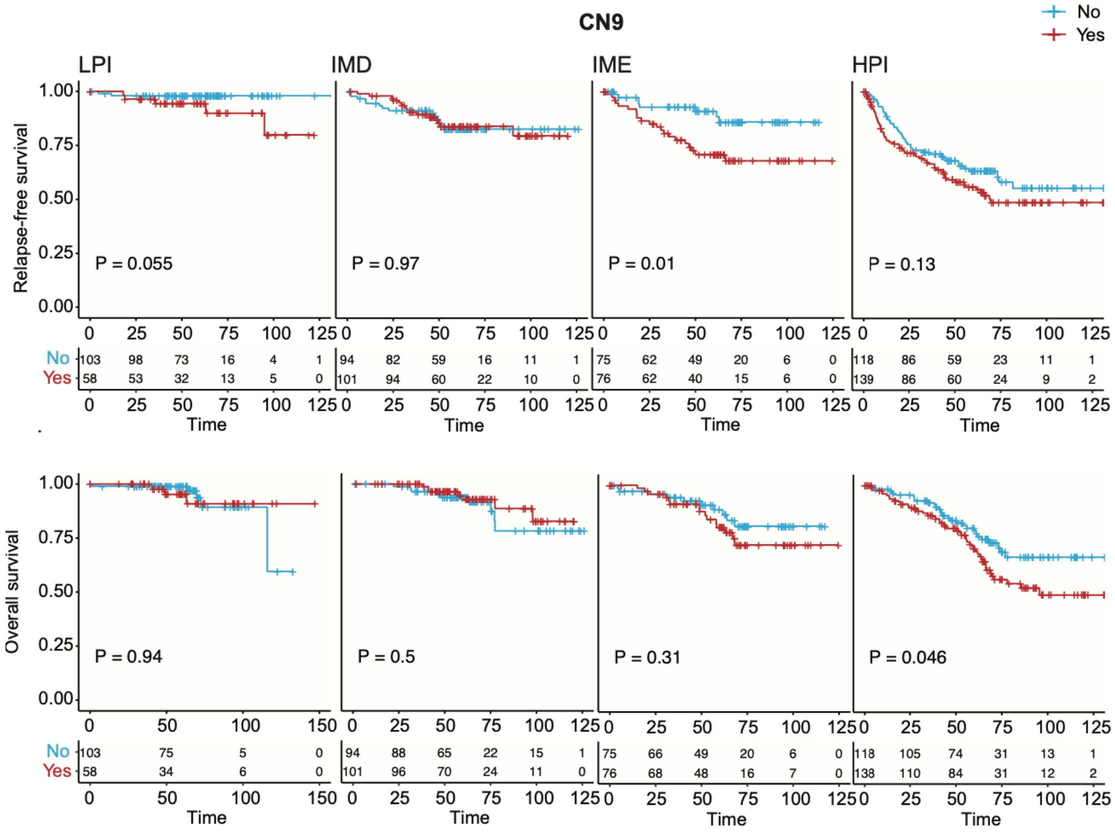

Fig. S21. Survival analysis of CN9 signature within the four subtypes. Distribution of OS and RFS between samples with activated CN9 signature and those without activation.

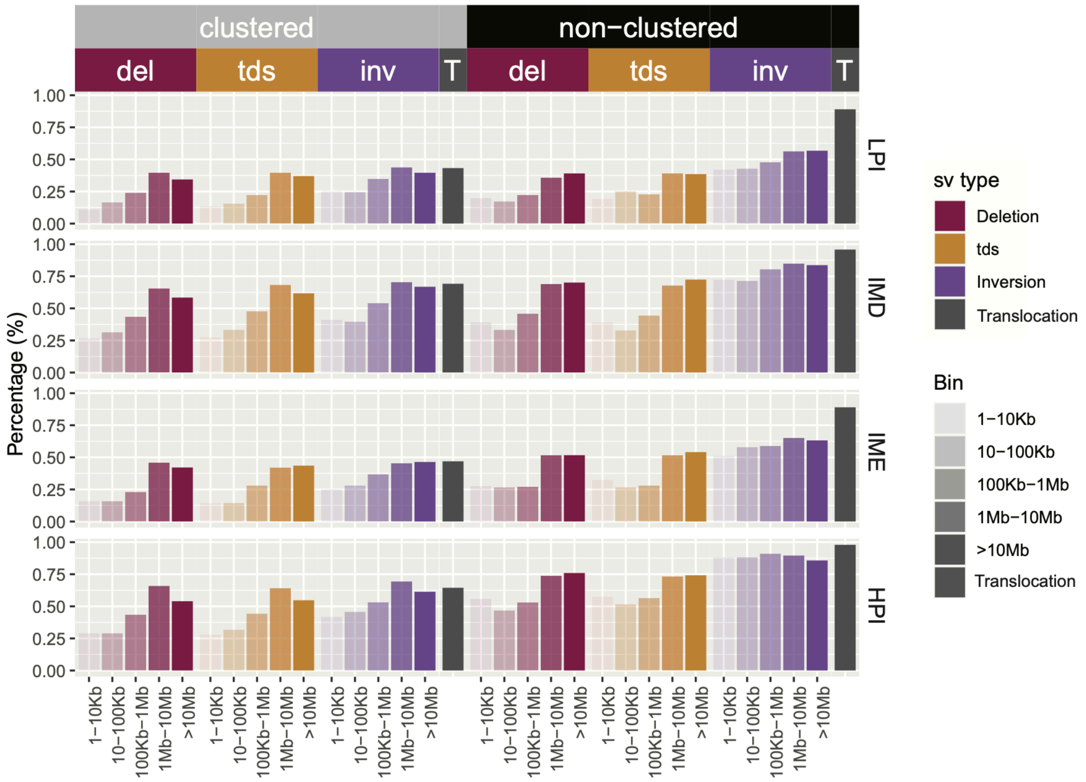

Fig. S22. The percentages of 32 structural variation (SV) features within the four subtypes. These features were annotated with SV type, size bin, and clustered/non-clustered status, making them suitable for subsequent signature decomposition.

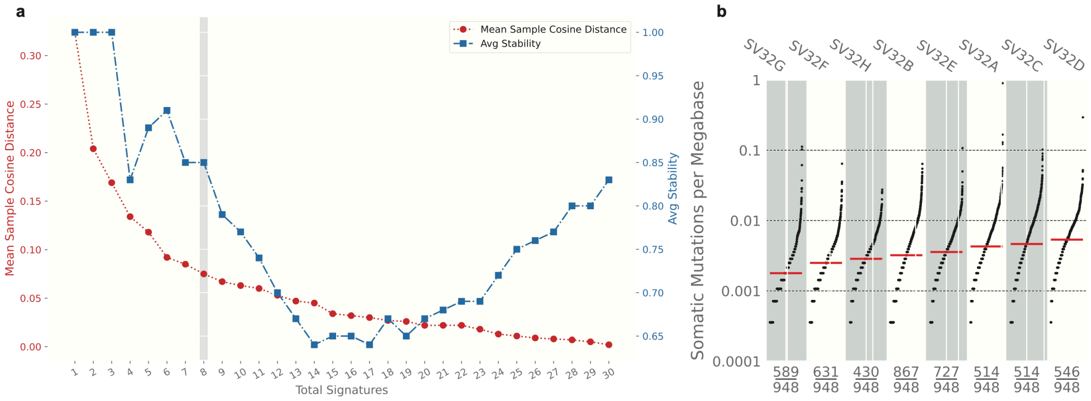

Fig. S23. Generation of SV signature. (**a**) The mean sample cosine distance and average stability show variations across the range of 1 to 30 structural SV signatures. The vertical gray bar indicates the optimal number of SV signatures selected based on cosine distance and average stability. (**b**) Distribution of mutations per megabase in the eight structural variation (SV) signatures.

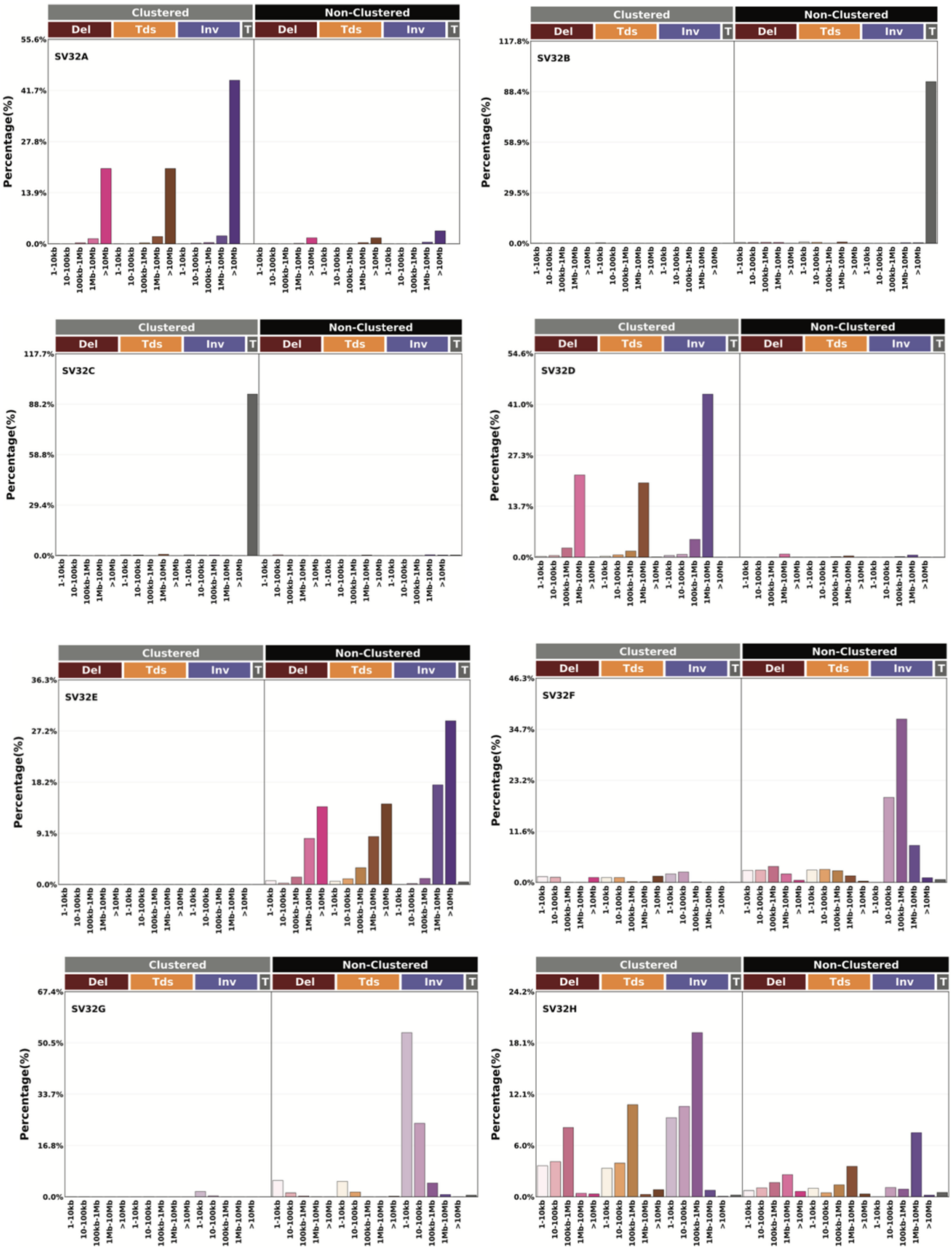

Fig. S24. A decomposition plot depicts the clustered status, SV type, and segment sizes associated with the eight SV signatures.

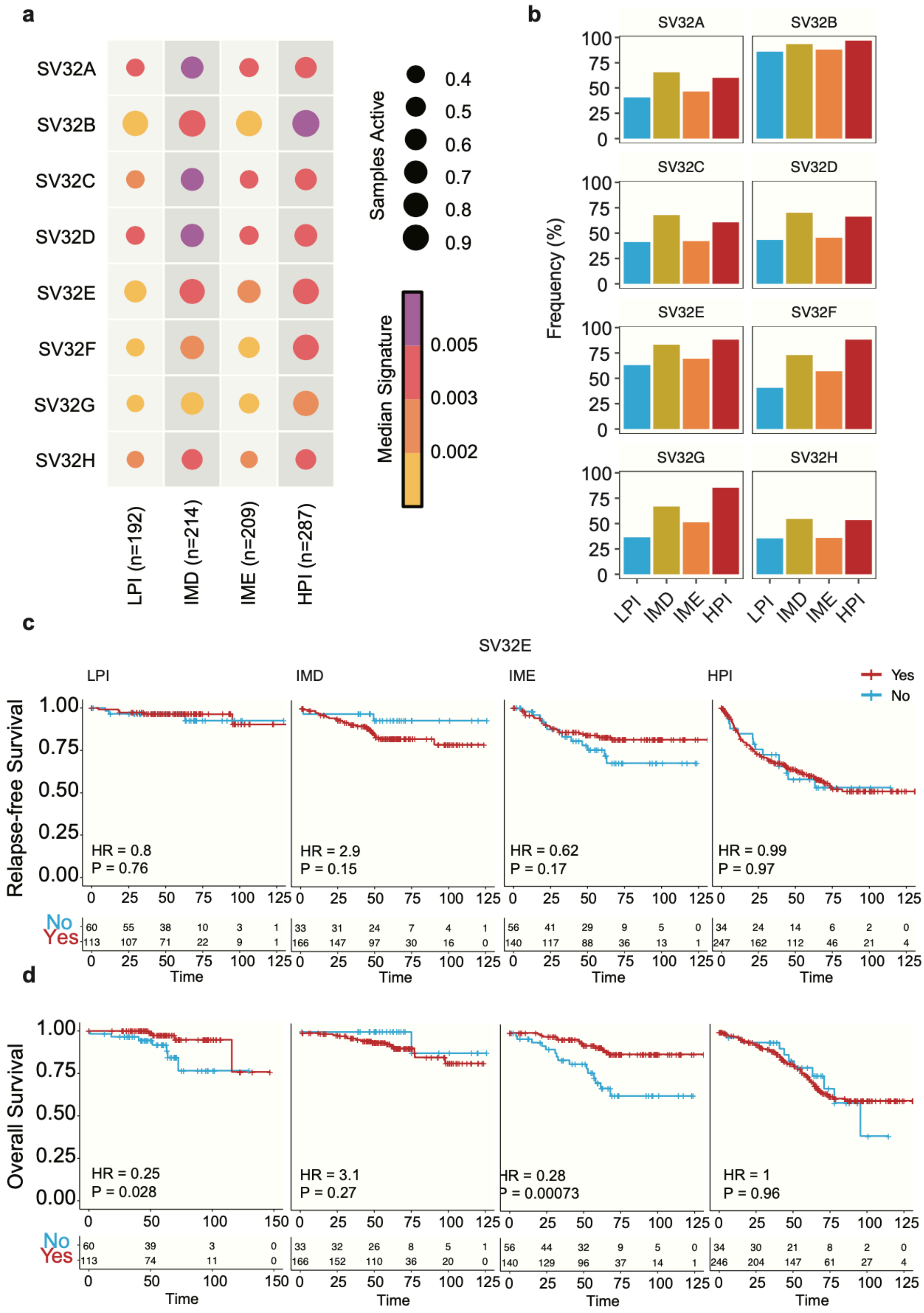

Fig. S25. The distribution and survival analysis of SV signatures within the four subtypes. (**a**) The count of mutations associated with mutational signatures across four subtypes. The size of the circle indicates the percentage of tumors expressing signature activity within each subtype, while the color reflects the median number of mutations per megabase associated with a specific signature in the respective subtype. (**b**) Bar plot depicting the frequency of SV signatures within the four subtypes. Distribution of OS (**c**) and RFS (**d**) between samples with activated SV32E signature and those without activation.

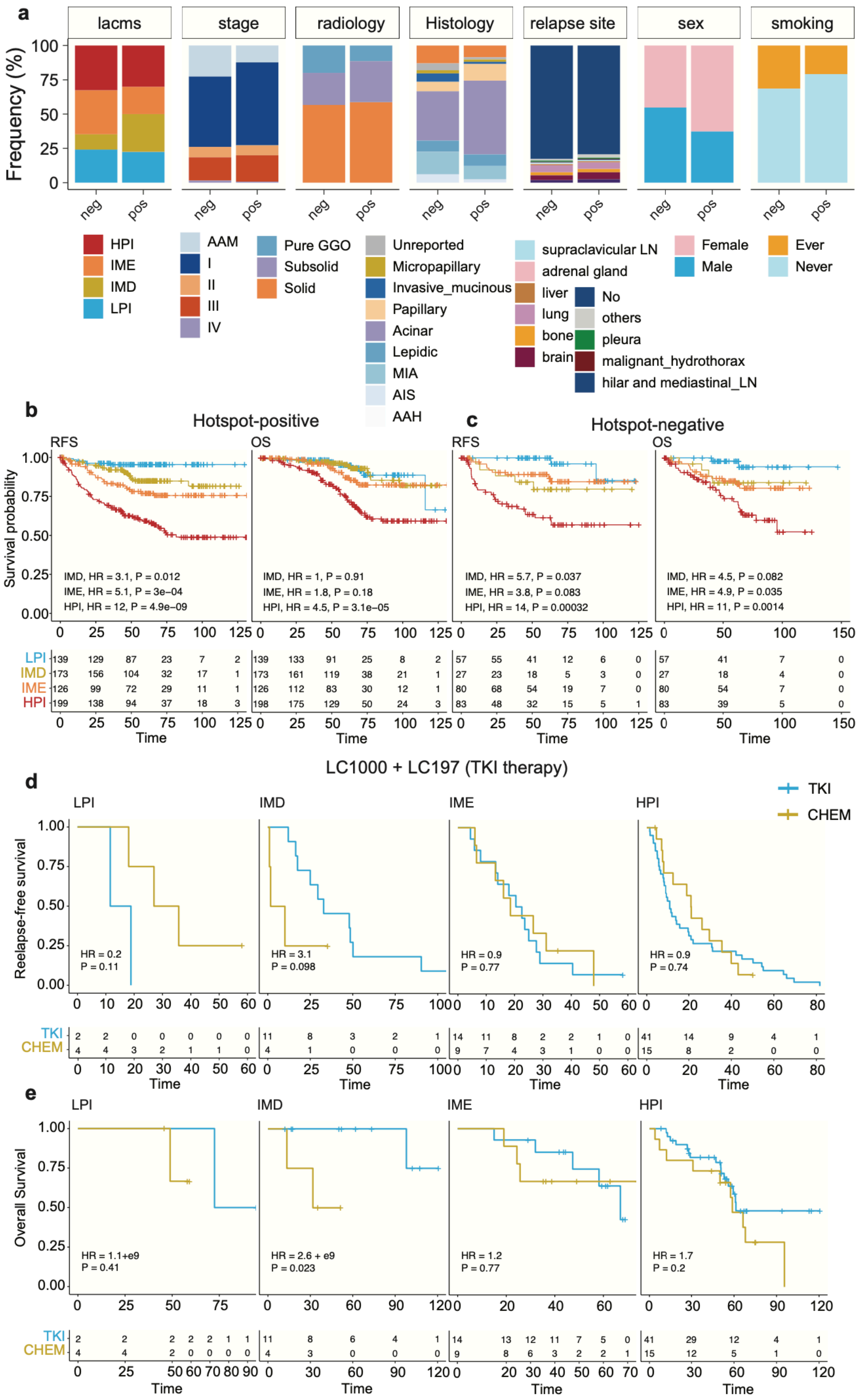
a
lacms
stage
radiology
Histology
relapse site
sex
smoking
Frequency (%)
HPI
IME
IMD
LPI
AAM
I
II
III
IV
Pure GGO
Subsolid
Solid
Unreported
Micropapillary
Invasive_mucinous
Papillary
Acinar
Lepidic
MIA
AIS
AAH
supraclavicular LN
adrenal gland
liver
lung
bone
brain
No
others
pleura
malignant_hydrothorax
hilar and mediastinal_LN
Female
Male
Ever
Never
b
Hotspot-positive
c
Hotspot-negative
RFS
OS
Survival probability
IMD, HR = 3.1, P = 0.012
IME, HR = 5.1, P = 3e−04
HPI, HR = 12, P = 4.9e−09
IMD, HR = 1, P = 0.91
IME, HR = 1.8, P = 0.18
HPI, HR = 4.5, P = 3.1e−05
IMD, HR = 5.7, P = 0.037
IME, HR = 3.8, P = 0.083
HPI, HR = 14, P = 0.00032
IMD, HR = 4.5, P = 0.082
IME, HR = 4.9, P = 0.035
HPI, HR = 11, P = 0.0014
Time
d
LC1000 + LC197 (TKI therapy)
TKI
CHEM
Reelapse-free survival
HR = 0.2
P = 0.11
HR = 3.1
P = 0.098
HR = 0.9
P = 0.77
HR = 0.9
P = 0.74
e
Overall Survival
HR = 1.1+e9
P = 0.41
HR = 2.6 + e9
P = 0.023
HR = 1.2
P = 0.77
HR = 1.7
P = 0.2

Fig. S26. Survival outcomes associated with TKI therapy in the four identified subtypes. (**a**) Distribution of the four subtypes and clinical phenotypes between the NCCN-positive and NCCN-negative groups. Relapse-free survival and overall survival of the four subtypes in the NCCN-positive (**b**) and NCCN-negative groups (**c**), respectively. Comparison of relapse-free survival (**d**) and overall survival (**e**) between patients receiving TKI therapy and chemotherapy in LPI, IMD, IME, and HPI subtypes from LC-1000 and LC-197 cohorts.

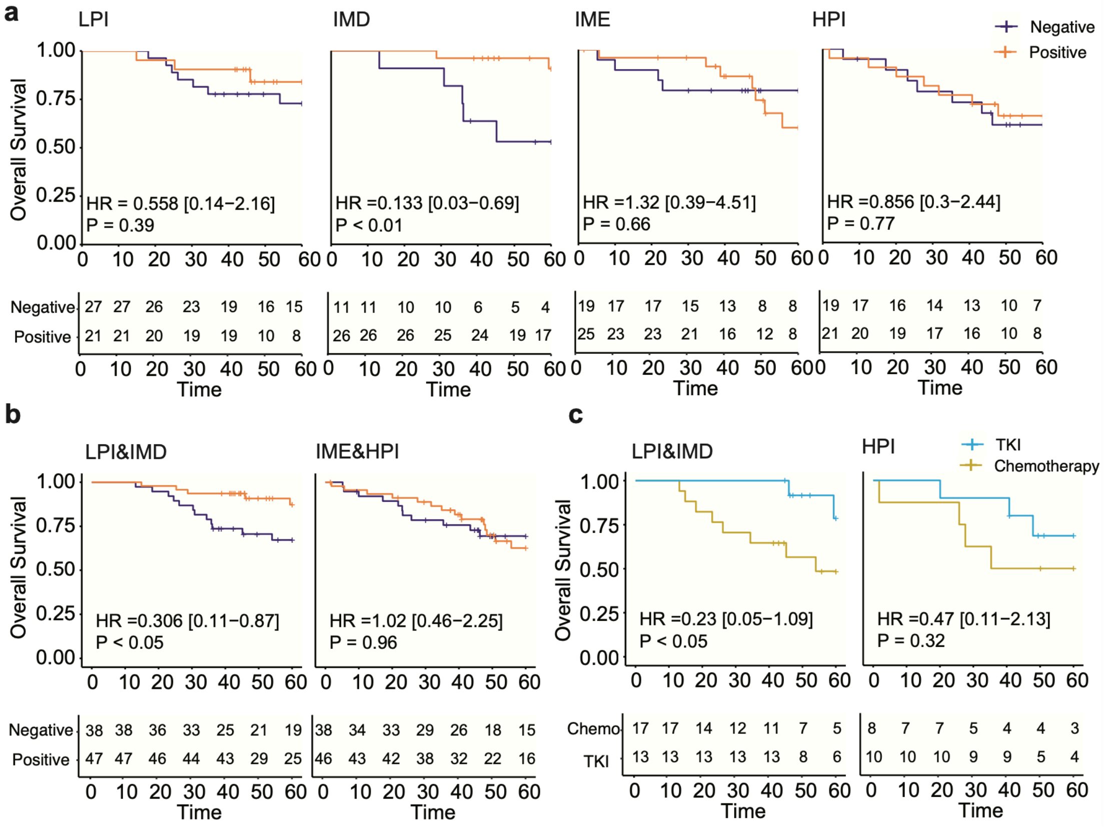

Fig. S27. Survival outcomes associated with TKI therapy of the four subtypes in OncoSG cohort. (**a**) The overall survival (OS) of patients classified as positive and negative for NCCN hotspots in LPI, IMD, IME, and HPI subtypes. (**b**) The OS of patients classified as positive and negative for NCCN hotspots in LPI&IMD and IME&HPI subtypes. (**c**) The comparison of OS between patients undergoing TKI therapy and chemotherapy in LPI&IMD and HPI subtypes

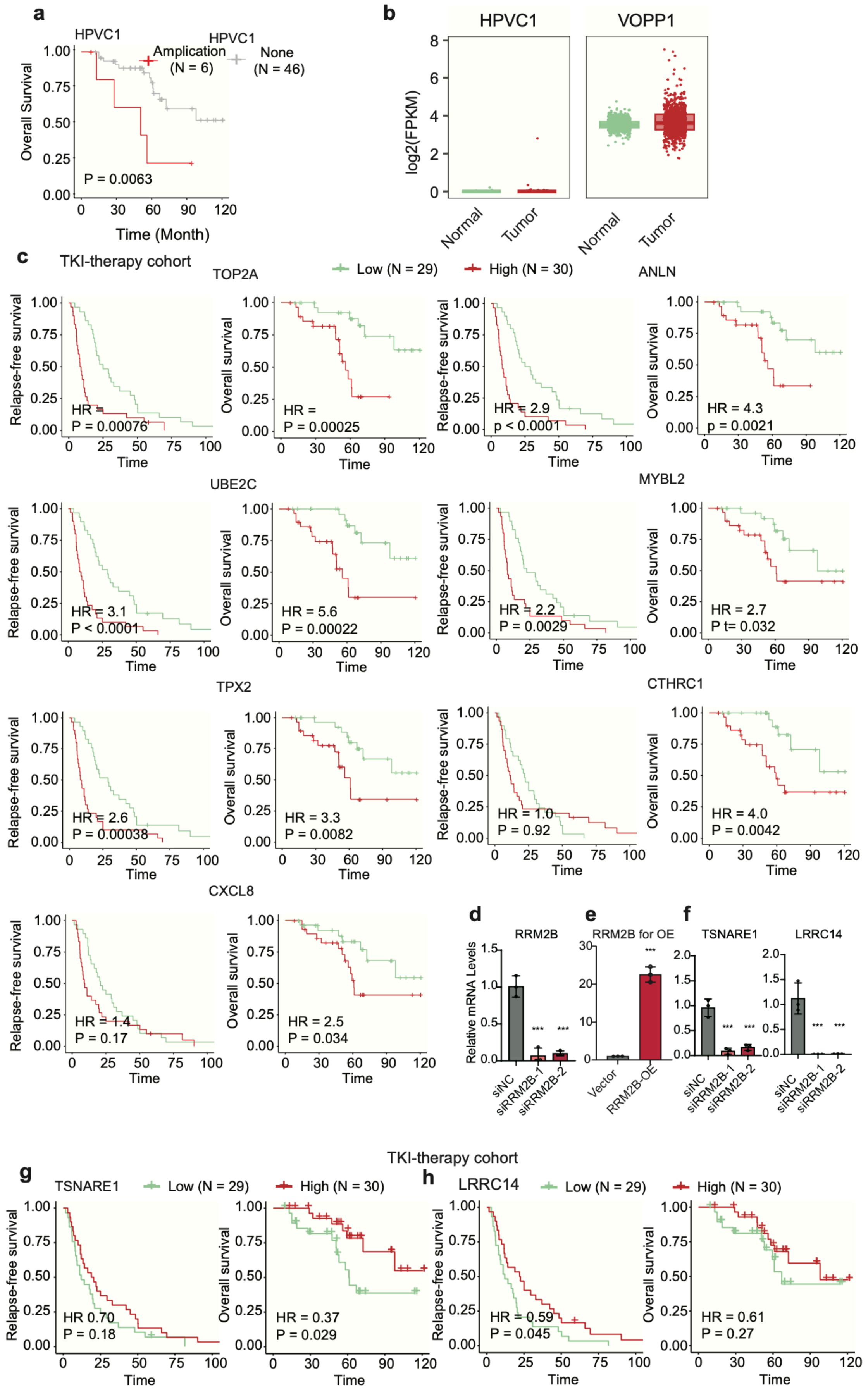

a
HPVC1
Amplication (N = 6)
HPVC1 None (N = 46)
Overall Survival
P = 0.0063
Time (Month)
b
HPVC1
VOPP1
log2(FPKM)
Normal
Tumor
c
TKI-therapy cohort
Low (N = 29)
High (N = 30)
TOP2A
ANLN
UBE2C
MYBL2
TPX2
CTHRC1
CXCL8
Relapse-free survival
Overall survival
Time
HR =
P = 0.00076
P = 0.00025
HR = 2.9
p < 0.0001
HR = 4.3
p = 0.0021
HR = 3.1
P < 0.0001
HR = 5.6
P = 0.00022
HR = 2.2
P = 0.0029
HR = 2.7
P t= 0.032
HR = 2.6
P = 0.00038
HR = 3.3
P = 0.0082
HR = 1.0
P = 0.92
HR = 4.0
P = 0.0042
HR = 1.4
P = 0.17
HR = 2.5
P = 0.034
d
RRM2B
Relative mRNA Levels
siNC
siRRM2B-1
siRRM2B-2
e
RRM2B for OE
Vector
RRM2B-OE
f
TSNARE1
LRRC14
TKI-therapy cohort
g
TSNARE1
Low (N = 29)
High (N = 30)
HR 0.70
P = 0.18
HR = 0.37
P = 0.029
h
LRRC14
Low (N = 29)
High (N = 30)
HR = 0.59
P = 0.045
HR = 0.61
P = 0.27

Fig. S28. Molecular events associated with response and resistance to TKI therapy. (**a**) Overall survival was assessed for patients with HPVC1 gain co-occurring with hotspots-positive in HPI subtypes, compared to those without genetic variants, among individuals who underwent TKI therapy in the LC-1000 cohort. (**b**) Expression distribution of HPVC1 and VOPP1. (**c**) Comparison of OS and RFS between the high and low expression groups based on the median values of VOPP1 up-regulated genes including TOP2A, UBEC2, ANLN, TPX2, MYBL2, CXCL8, CTHRC1 in TKI-therapy patients. (**d**) Relative mRNA expression levels of RRM2B in PC9 cells under negative control (siNC) and RRM2B silencing (siRRM2B) conditions. (**e**) Relative mRNA expression levels of RRM2B in PC9-OR cells under control (Vector) and RRM2B-overexpression (RRM2B) conditions. (**f**) Relative mRNA expression levels of RRM2B-regulated genes, including TSNARE1 and LRRC14, in PC9 under control (siNC) and RRM2B silencing (siRRM2B) conditions. Comparison of OS and RFS between the high and low expression groups based on the median values of TSNARE1 (**g**) and LRRC14 (**h**) in TKI-therapy patients.

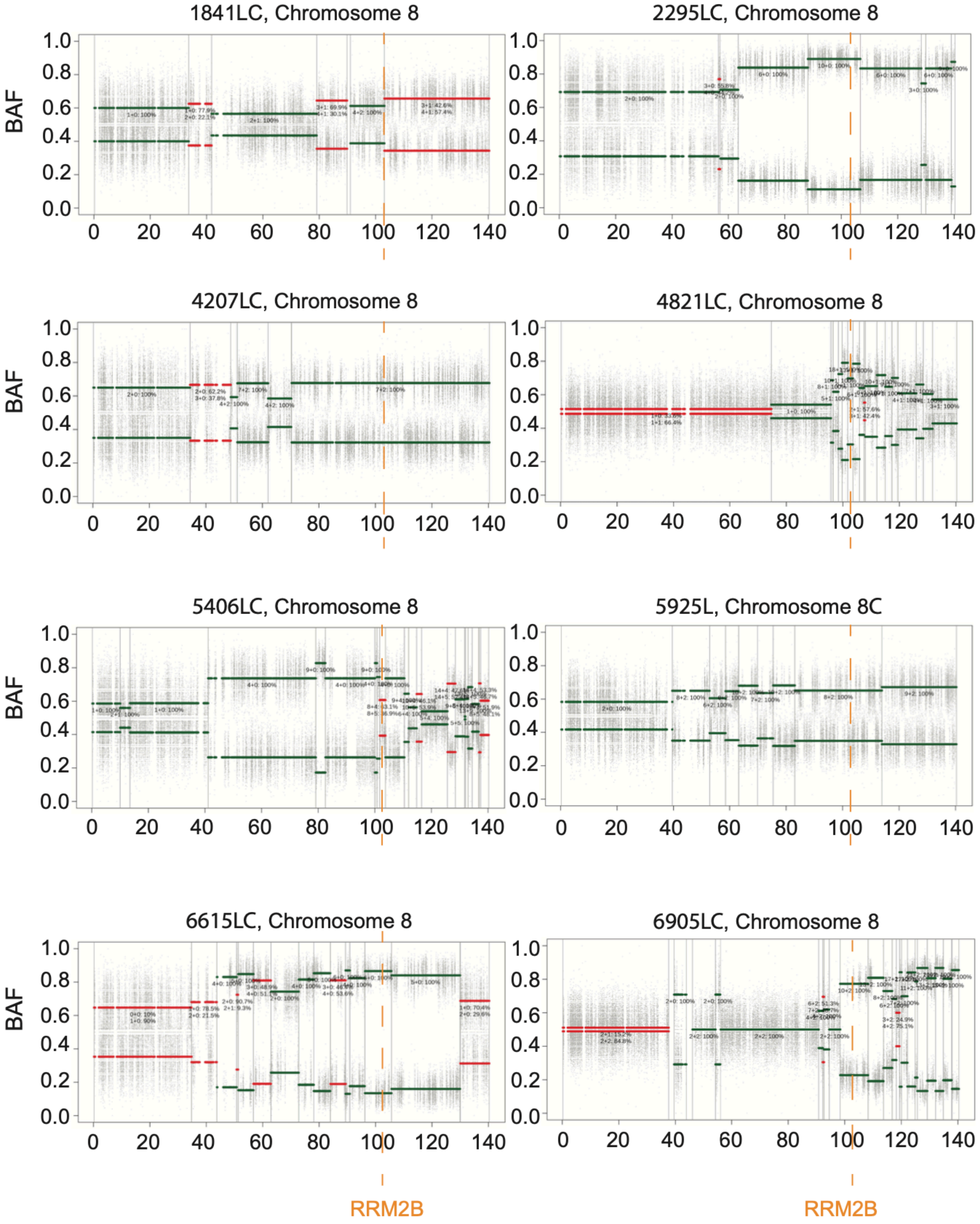

Fig. S29. The copy number profile of B allele frequency (BAF) throughout chromosome 8 (the chromosomal location of RRM2B) for representative RRM2B-amplified samples. Subclonal marked in red, clonal marked in dark green, and the RRM2B locus position marked in orange.

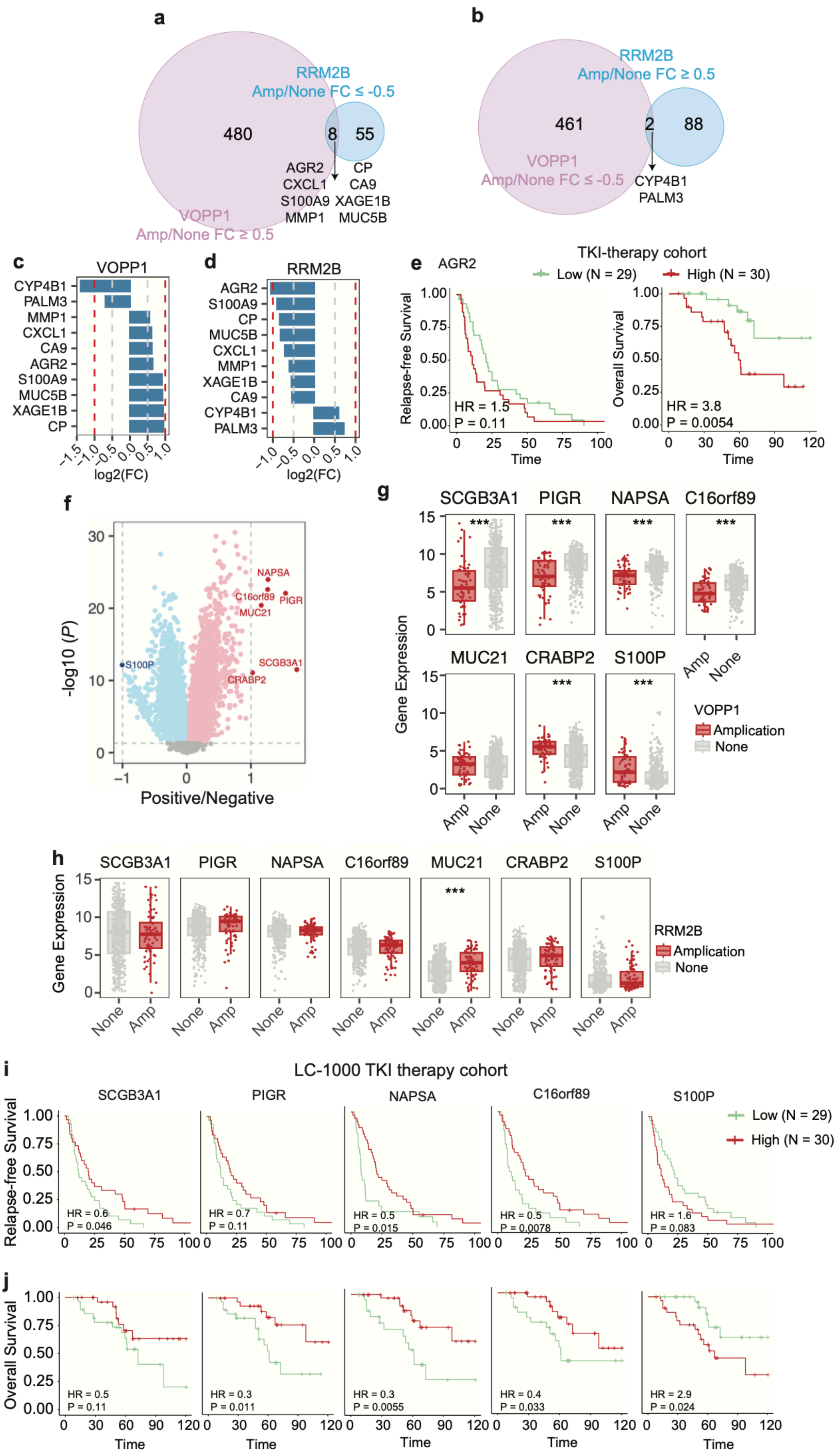
a
RRM2B
Amp/None FC ≤ -0.5
480
8
55
AGR2
CXCL1
S100A9
MMP1
CP
CA9
XAGE1B
MUC5B
VOPP1
Amp/None FC ≥ 0.5
b
RRM2B
Amp/None FC ≥ 0.5
461
2
88
VOPP1
Amp/None FC ≤ -0.5
CYP4B1
PALM3
c
VOPP1
log2(FC)
d
RRM2B
log2(FC)
e
AGR2
TKI-therapy cohort
Low (N = 29)
High (N = 30)
Relapse-free Survival
HR = 1.5
P = 0.11
Overall Survival
HR = 3.8
P = 0.0054
Time
f
-log10 (P)
NAPSA
C16orf89
PIGR
MUC21
S100P
SCGB3A1
CRABP2
Positive/Negative
g
SCGB3A1
PIGR
NAPSA
C16orf89
MUC21
CRABP2
S100P
Gene Expression
VOPP1
Amplication
None
h
SCGB3A1
PIGR
NAPSA
C16orf89
MUC21
CRABP2
S100P
Gene Expression
RRM2B
Amplication
None
i
LC-1000 TKI therapy cohort
SCGB3A1
PIGR
NAPSA
C16orf89
S100P
Low (N = 29)
High (N = 30)
Relapse-free Survival
HR = 0.6
P = 0.046
HR = 0.7
P = 0.11
HR = 0.5
P = 0.015
HR = 0.5
P = 0.0078
HR = 1.6
P = 0.083
j
Overall Survival
HR = 0.5
P = 0.11
HR = 0.3
P = 0.011
HR = 0.3
P = 0.0055
HR = 0.4
P = 0.033
HR = 2.9
P = 0.024
Time

Fig. S30. RRM2B and VOPP1 amplification-regulated genes. (**a**) A venn plot illustrates genes that are significantly up-regulated in the presence of VOPP1 amplification and down-regulated in the presence of RRM2B amplification. (**b**) A venn plot illustrates genes that are significantly up-regulated in the presence of RRM2B amplification and down-regulated in the presence of VOPP1 amplification. The bar plot illustrates the fold change of genes from distributions and between VOPP1 (**c**) or RRM2B (**d**) amplification and the non-variant group. (**e**) Comparison of OS and RFS between the high and low expression groups based on the median values of AGR2 in TKI-therapy patients. (**f**) The volcano plot illustrates the distribution of fold change values and *P*-value for DEGs between NCCN hotspot-positive and negative groups. (**g**) Expression distribution of seven DEGs (SCGB3A1, PIGR, NAPSA, C16orf89, MUC21, CRABP2 and S100P) between the VOPP1 gain and none variant group. (**h**) Expression distribution of seven DEGs (SCGB3A1, PIGR, NAPSA, C16orf89, MUC21, CRABP2 and S100P) between the RRM2B gain and none variant group. Comparison of OS (**i**) and RFS (**j**) between the high and low expression groups based on the median values of five DEGs in TKI-therapy patients.

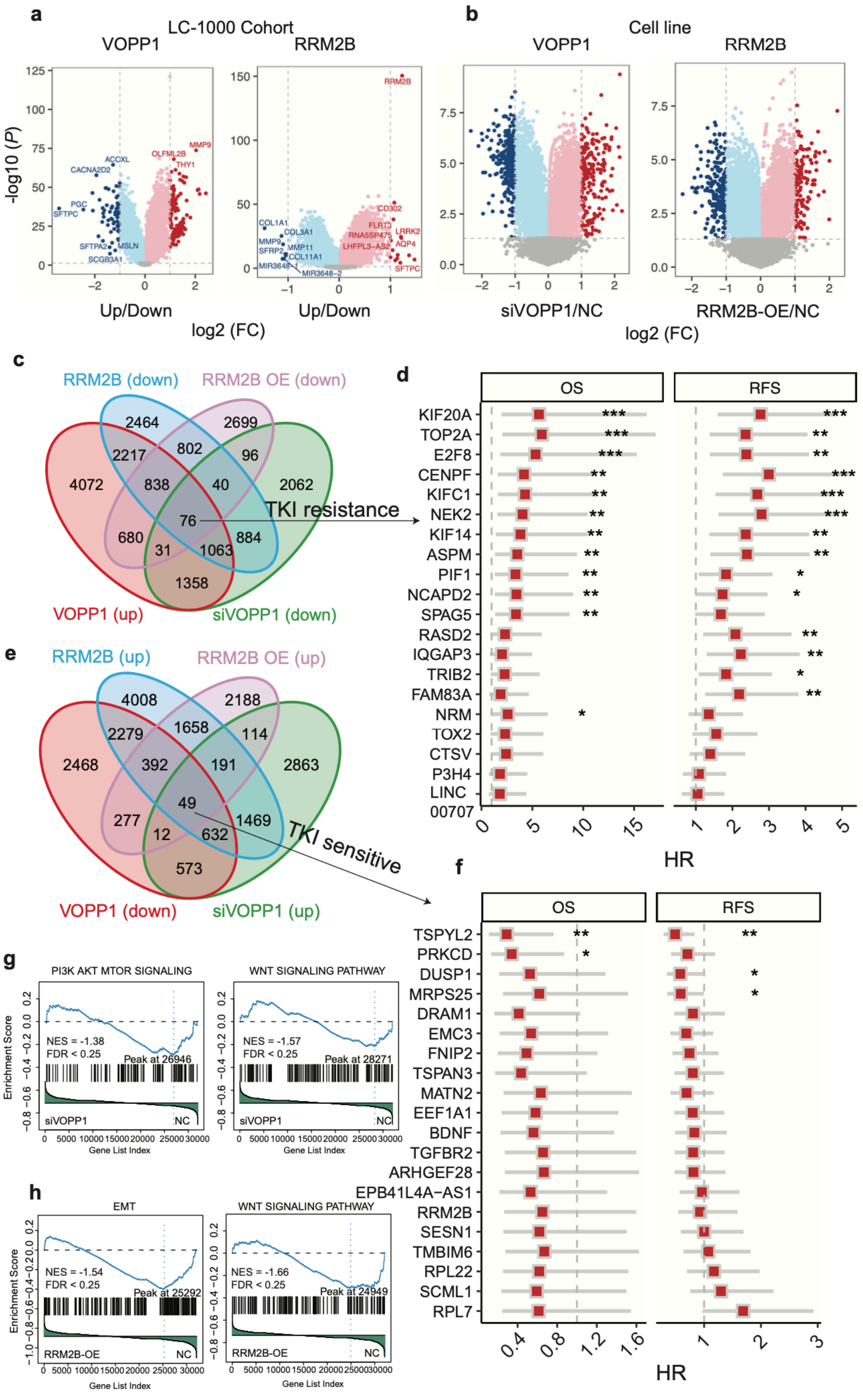

a
LC-1000 Cohort
VOPP1
RRM2B
-log10 (P)
Up/Down
log2 (FC)
b
Cell line
VOPP1
RRM2B
siVOPP1/NC
RRM2B-OE/NC
log2 (FC)
c
RRM2B (down)
RRM2B OE (down)
VOPP1 (up)
siVOPP1 (down)
TKI resistance
d
OS
RFS
HR
e
RRM2B (up)
RRM2B OE (up)
VOPP1 (down)
siVOPP1 (up)
TKI sensitive
f
OS
RFS
HR
g
PI3K AKT MTOR SIGNALING
WNT SIGNALING PATHWAY
NES = -1.38
FDR < 0.25
Peak at 26946
NES = -1.57
FDR < 0.25
Peak at 28271
Enrichment Score
Gene List Index
h
EMT
WNT SIGNALING PATHWAY
NES = -1.54
FDR < 0.25
Peak at 25292
NES = -1.66
FDR < 0.25
Peak at 24949

Fig. S31. Potential downstream regulatory genes and pathways of RRM2B and VOPP1 associated with TKI treatment. (**a**) The volcano plot illustrates the distribution of P-values and Fold Changes for differentially expressed genes of VOPP1 (left) and RRM2B (right), divided into up-regulated and down-regulated groups based on the median value in LC-1000 cohort. (**b**) The volcano plot illustrates the distribution of P-values and Fold Changes for differentially expressed genes of siVOPP1 vs NC (left) and RRM2B-OE vs NC (right) in cell line. (**c**) The Venn diagram illustrates the intersectional relationships among four gene sets: TKI-potentially upregulated VOPP1 (tissue), potentially downregulated RRM2B (tissue), RRM2B-OE-induced downregulation (cell line), and siVOPP1-induced downregulation (cell line). (**d**) Univariate Cox analysis of potential TKI resistance genes (four-group intersection in Panel C) reveals the top 20 genes with the highest HR in the TKI-treated cohort, presenting their recurrence-free survival (RFS) and overall survival (OS) outcomes. (**e**) The Venn diagram illustrates the intersectional relationships among four gene sets: TKI-potentially upregulated RRM2B (tissue), potentially downregulated VOPP1 (tissue), RRM2B-OE-induced upregulation (cell line), and siVOPP1-induced upregulation (cell line). (**f**) Univariate Cox analysis of potential TKI-sensitive genes (four-group intersection in Panel E) identified the top 20 genes with negative HR in the TKI-treated cohort, presenting their RFS and OS outcomes. (**g**) GSEA analysis of TKI resistance-related pathways (PIK3CA-AKT-mTOR and WNT signaling) between siVOPP1 and control groups. (**h**) GSEA analysis of TKI sensitive-related pathways (EMT and WNT signaling) between RRM2B-OE and control groups.

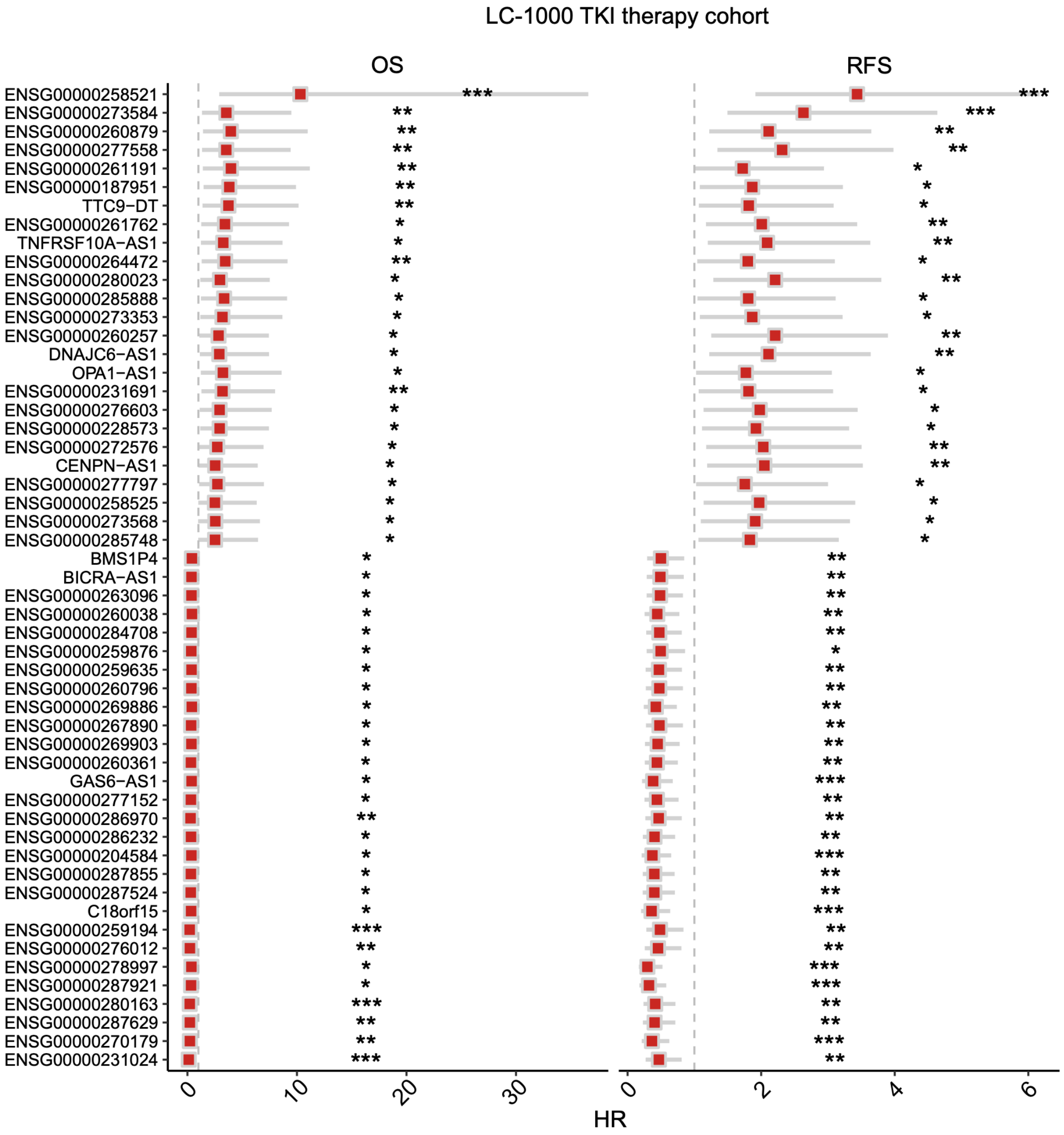

Fig. S32. The univariate Cox analysis in the LC1000 cohort revealed TKI therapy-related non-coding RNAs.

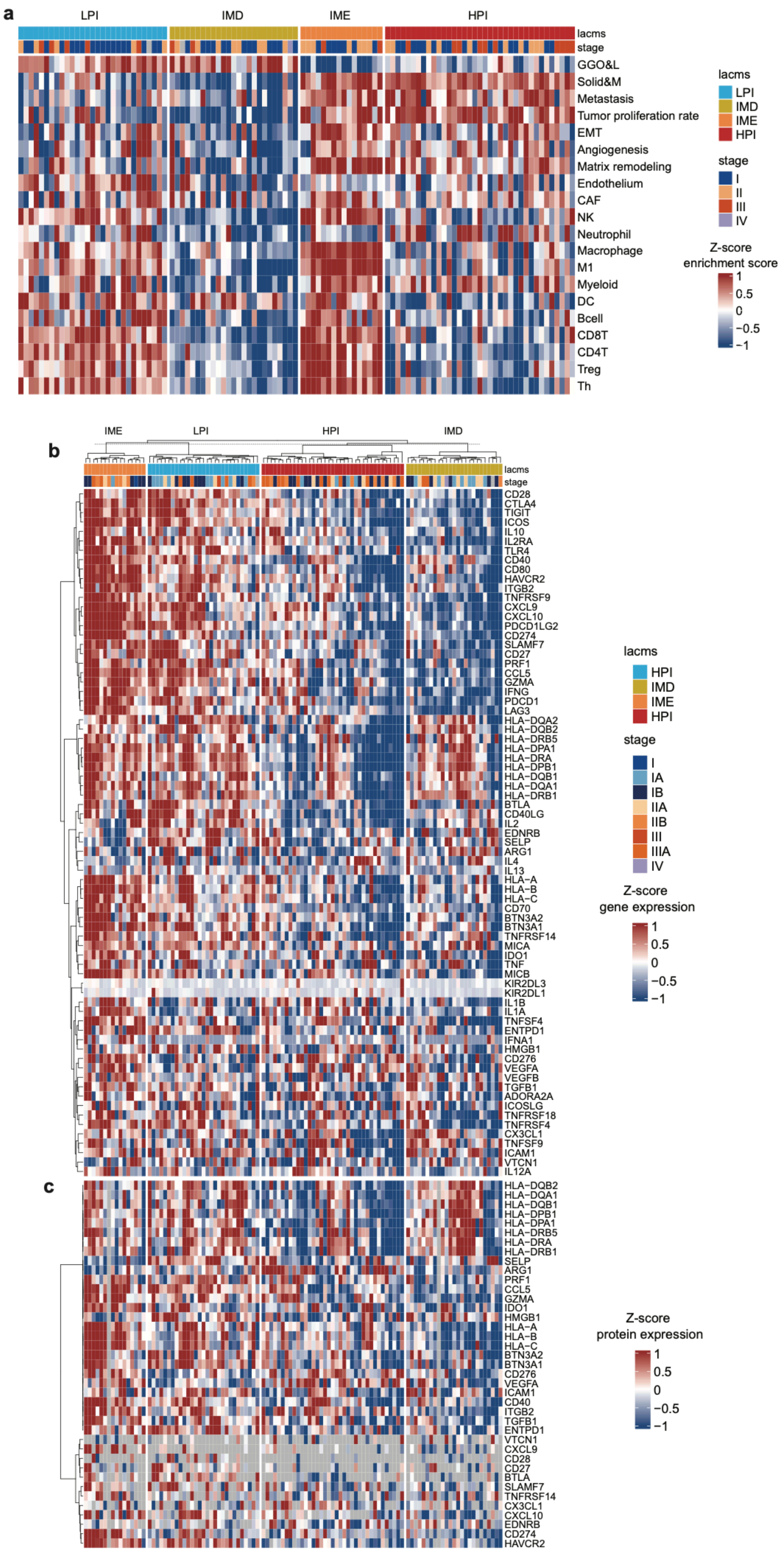
a
LPI
IMD
IME
HPI
lacms
stage
GGO&L
Solid&M
Metastasis
Tumor proliferation rate
EMT
Angiogenesis
Matrix remodeling
Endothelium
CAF
NK
Neutrophil
Macrophage
M1
Myeloid
DC
Bcell
CD8T
CD4T
Treg
Th
lacms
LPI
IMD
IME
HPI
stage
I
II
III
IV
Z-score
enrichment score
1
0.5
0
−0.5
−1
b
IME
LPI
HPI
IMD
lacms
stage
CD28
CTLA4
TIGIT
ICOS
IL10
IL2RA
TLR4
CD40
CD80
HAVCR2
ITGB2
TNFRSF9
CXCL9
CXCL10
PDCD1LG2
CD274
SLAMF7
CD27
PRF1
CCL5
GZMA
IFNG
PDCD1
LAG3
HLA−DQA2
HLA−DQB2
HLA−DRB5
HLA−DPA1
HLA−DRA
HLA−DPB1
HLA−DQB1
HLA−DQA1
HLA−DRB1
BTLA
CD40LG
IL2
EDNRB
SELP
ARG1
IL4
IL13
HLA−A
HLA−B
HLA−C
CD70
BTN3A2
BTN3A1
TNFRSF14
MICA
IDO1
TNF
MICB
KIR2DL3
KIR2DL1
IL1B
IL1A
TNFSF4
ENTPD1
IFNA1
HMGB1
CD276
VEGFA
VEGFB
TGFB1
ADORA2A
ICOSLG
TNFRSF18
TNFRSF4
CX3CL1
TNFSF9
ICAM1
VTCN1
IL12A
lacms
HPI
IMD
IME
HPI
stage
I
IA
IB
IIA
IIB
III
IIIA
IV
Z-score
gene expression
1
0.5
0
−0.5
−1
c
HLA−DQB2
HLA−DQA1
HLA−DQB1
HLA−DPB1
HLA−DPA1
HLA−DRB5
HLA−DRA
HLA−DRB1
SELP
ARG1
PRF1
CCL5
GZMA
IDO1
HMGB1
HLA−A
HLA−B
HLA−C
BTN3A2
BTN3A1
CD276
VEGFA
ICAM1
CD40
ITGB2
TGFB1
ENTPD1
VTCN1
CXCL9
CD28
CD27
BTLA
SLAMF7
TNFRSF14
CX3CL1
CXCL10
EDNRB
CD274
HAVCR2
Z-score
protein expression
1
0.5
0
−0.5
−1

Fig. S33. Distribution of immune checkpoint expression at the gene and protein levels within the four subtypes. (**a**) Heatmap displaying the scores of 20 radio-pathology and tumor microenvironment (TME) gene expression signatures for the four subtypes in the CPTAC cohort. Heatmap illustrating the distribution of immune checkpoints at both the gene (**b**) and protein (**c**) levels.

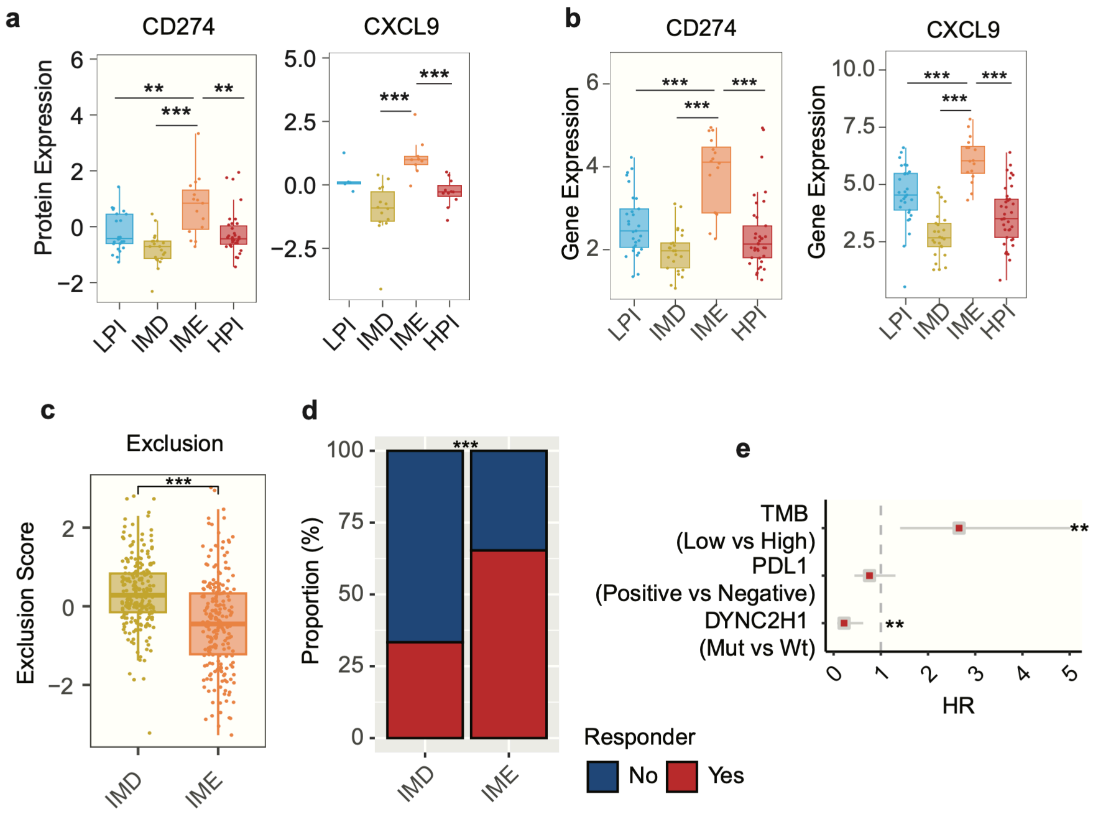

Fig. S34. IMD and IME Immune Responses. Boxplot illustrating the distribution of immune checkpoint genes CD274 and CXCL9 both at the protein (**a**) gene (**b**) levels, within the four subtypes in the CPTAC cohort. (**c**) Boxplot illustrates the exclusion score evaluated by TIDE software between IMD and IME subtypes. (**d**) Bar graph depicts the proportion of responder and non-responder predicted by TIDE software between IMD and IME subtypes. (**e**) The multivariate Cox analysis of DYNC2H1 mutation in PFS.
